# Biochemical Competition Makes Fatty-Acid β-Oxidation Vulnerable to Substrate Overload

**DOI:** 10.1371/journal.pcbi.1003186

**Published:** 2013-08-15

**Authors:** Karen van Eunen, Sereh M. J. Simons, Albert Gerding, Aycha Bleeker, Gijs den Besten, Catharina M. L. Touw, Sander M. Houten, Bert K. Groen, Klaas Krab, Dirk-Jan Reijngoud, Barbara M. Bakker

**Affiliations:** 1 Department of Pediatrics, Center for Liver, Digestive and Metabolic Diseases, University of Groningen, University Medical Center Groningen, Groningen, The Netherlands; 2 Netherlands Consortium for Systems Biology, Amsterdam, The Netherlands; 3 Department of Laboratory Medicine, Center for Liver, Digestive and Metabolic Diseases, University of Groningen, University Medical Center Groningen, Groningen, The Netherlands; 4 Laboratory Genetic Metabolic Diseases, Departments of Clinical Chemistry and Pediatrics, Emma Children's Hospital, Academic Medical Center, University of Amsterdam, Amsterdam, The Netherlands; 5 Department of Molecular Cell Physiology, Netherlands Institute for Systems Biology, VU University Amsterdam, Amsterdam, The Netherlands; University of Virginia, United States of America

## Abstract

Fatty-acid metabolism plays a key role in acquired and inborn metabolic diseases. To obtain insight into the network dynamics of fatty-acid β-oxidation, we constructed a detailed computational model of the pathway and subjected it to a fat overload condition. The model contains reversible and saturable enzyme-kinetic equations and experimentally determined parameters for rat-liver enzymes. It was validated by adding palmitoyl CoA or palmitoyl carnitine to isolated rat-liver mitochondria: without refitting of measured parameters, the model correctly predicted the β-oxidation flux as well as the time profiles of most acyl-carnitine concentrations. Subsequently, we simulated the condition of obesity by increasing the palmitoyl-CoA concentration. At a high concentration of palmitoyl CoA the β-oxidation became overloaded: the flux dropped and metabolites accumulated. This behavior originated from the competition between acyl CoAs of different chain lengths for a set of acyl-CoA dehydrogenases with overlapping substrate specificity. This effectively induced competitive feedforward inhibition and thereby led to accumulation of CoA-ester intermediates and depletion of free CoA (CoASH). The mitochondrial [NAD^+^]/[NADH] ratio modulated the sensitivity to substrate overload, revealing a tight interplay between regulation of β-oxidation and mitochondrial respiration.

## Author Summary

Lipid metabolism plays an important role in the development of metabolic syndrome, a major risk factor for cardiovascular disease and diabetes. Furthermore, inborn errors in lipid oxidation cause rare, but severe diseases in children. To obtain more insight into the response of lipid oxidation to dietary and medical interventions, we constructed a computational model. The model correctly simulated the rate of lipid oxidation and the time courses of most acyl carnitines. The latter are used as diagnostic markers in blood. Subsequently, we subjected the model to an increased supply of lipids, as often happens in obese people. We discovered that the lipid-oxidation machinery easily becomes overloaded, very much like a highway during rush hours: the more cars enter the road, the slower they proceed and the more they clog the road. Analogously, an overload of lipids slowed down the lipid oxidation and led to an accumulation of intermediate metabolites in the pathway. Potential protection mechanisms of cells consist of restricted entry of lipids into the oxidation pathway or efficient downstream processing of reaction products. In future research we will use the model to test dietary or medical interventions *in silico* and thereby guide the development of new treatment and prevention strategies.

## Introduction

Pathophysiological mechanisms underlying acquired and inborn metabolic diseases, such as type-2 diabetes and deficiencies in the fatty-acid oxidation, are largely elusive. Although we know many important molecular factors, yet the complexity of the metabolic and regulatory network hampers elucidating the relation between the primary disease factors and their systemic effects [1], [2]. Moreover, the experimental accessibility of large parts of the metabolic networks is limited. Computational kinetic models yield insight into the dynamics of metabolic networks and make predictions about the parts that are experimentally inaccessible.

Fatty-acid (FA) β-oxidation is a prime example of a pathway involved in many diseases, but for which it is difficult to acquire a complete and quantitative view on the relation between metabolite concentrations and fluxes. Insulin resistance, one of the hallmarks of metabolic syndrome, is strongly associated with elevated levels of free FAs [3]. It has been argued that an imbalance between cellular FA uptake and oxidation leads to accumulation of FAs and other lipid molecules in the cytosol, which in turn causes insulin resistance [4], [5]. Others showed that a functioning acyl-CoA uptake into mitochondria is needed to develop insulin resistance, leading to the hypothesis that intermediates of FA β-oxidation are part of the problem [6]. Since the acyl-CoA intermediates are difficult to measure, conclusions are often based on acyl-carnitine levels in the blood [7], [8], which are interpreted as a reflection of acyl-CoA concentrations in the mitochondria. Similar limitations hamper the understanding of systemic effects of enzyme deficiencies in the FA β-oxidation and their impact on global energy and glucose regulation [9], [10]. Clearly, a more direct view on the dynamics of β-oxidation intermediates is urgently needed.

**Figure 1:**
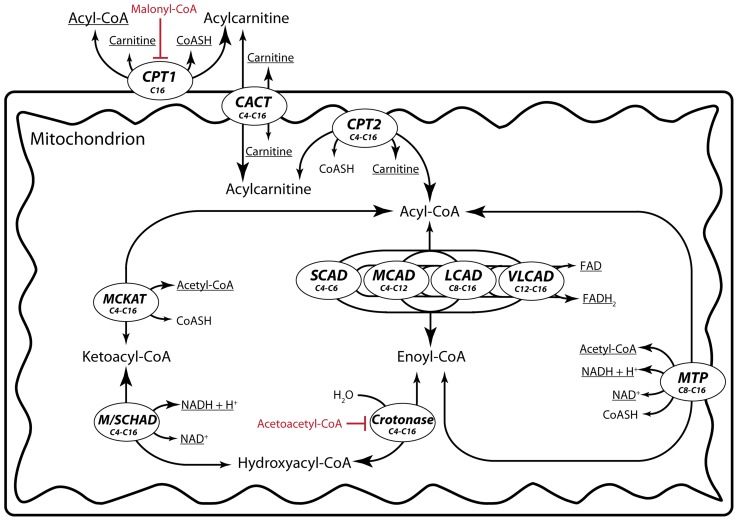
Schematic overview of FA β-oxidation as it is modeled in this study. Enzymes are depicted in italics bold face, metabolites in regular and allosteric inhibitors in red. CPT1: carnitine-palmitoyl transferase 1 (E.C. 2.3.1.21), CACT: carnitine-acyl-carnitine translocase, CPT2: carnitine-palmitoyl transferase 2 (E.C. 2.3.1.21), VLCAD: very-long-chain acyl-CoA dehydrogenase (E.C. 1.3.99.3), LCAD: long-chain acyl-CoA dehydrogenase (E.C. 1.3.99.3), MCAD: medium-chain acyl-CoA dehydrogenase (E.C. 1.3.99.3), SCAD short-chain acyl-CoA dehydrogenase (E.C.1.3.8.1 for butanoyl CoA (C4) (was previously E.C. 1.3.99.2) and E.C. 1.3.99.3 for hexanoyl CoA (C6)), MTP: mitochondrial trifunctional protein (E.C. 4.2.1.17, E.C. 1.1.1.211 and E.C. 2.3.1.16), M/SCHAD: medium/short-chain hydroxyacyl-CoA dehydrogenase (E.C. 1.1.1.35), MCKAT: medium-chain ketoacyl-CoA thiolase (E.C. 2.3.1.16). The chain-length specificity as included in the model is indicated for each enzyme (e.g. C6–C12). The boundary metabolites which are kept at fixed concentrations are underlined. The direction of the flux is from the small to large arrow heads.

A careful look at the basic biochemistry of the FA β-oxidation reveals complex interactions, of which the implications have never been investigated (Figure 1). First, it is a cyclic pathway. In each cycle the acyl-CoA substrate is shortened by two carbon atoms and the product is a substrate for the next cycle. Second, the shortened acyl-CoA product competes with the substrate for a set of enzymes with overlapping chain-length specificity. The complete breakdown of palmitoyl CoA, an acyl CoA with 16 carbon atoms, to 8 molecules of acetyl CoA (C2) requires seven reaction cycles. There are, however, only four acyl-CoA dehydrogenases and two parallel sets of enzymes for the further conversion of enoyl CoA (Figure 1). This results in a competition in two ways: substrates of different chain lengths compete for common enzymes, while enzymes with overlapping specificity compete for common substrates. The competition between substrates generates a feedforward inhibition in the network: the more palmitoyl CoA molecules enters the pathway, the more enzyme molecules they occupy, which are then not available for shorter acyl CoAs downstream in the pathway. Third, the two parallel pathways for the breakdown of enoyl-CoA molecules are of a different nature. Enoyl CoAs are either converted by a sequence of three enzymes (crotonase, medium/short-chain hydroxyacyl-CoA dehydrogenase (M/SCHAD) and medium-chain ketoacyl-CoA thiolase (MCKAT)) or via the mitochondrial trifunctional protein (MTP), which catalyzes the entire sequence of reactions (Figure 1). At present it is unclear how these properties affect the pathway behavior.

In contrast to the myriad of models of carbohydrate metabolism, we know of only two kinetic models of FA β-oxidation and each of these ignores the above biochemical interactions. The first model [11] contains only a single cycle of β-oxidation and thereby lacks the competition between the pathway substrate and its downstream products. The second model does comprise the complete conversion of acyl CoAs to acetyl CoA, but the authors overlooked that this should lead to competition and modeled it as if distinct pools of each enzyme existed for each substrate [12].

In this study we present and validate a quantitative kinetic model of the mitochondrial FA β-oxidation, which explicitly includes molecular competition. This model reveals how the complex biochemical wiring of the network gives rise to non-intuitive or ‘emergent’ behavior [13]. We will show *i)* how the competition renders the pathway vulnerable to substrate overload; *ii)* how the robustness of the system can be modulated; and *iii)* what the role is of the experimentally inaccessible CoA esters in this process.

## Results

### Model construction

We constructed a model for mitochondrial FA β-oxidation with all enzyme reactions and transporters (Figure 1). The reactions correspond to the mitochondrial β-oxidation module in the updated human metabolic reconstruction [14], with the difference that we tailored them to rat liver. Thus, we added the long-chain acyl-CoA dehydrogenase (LCAD), which is involved in rodent β-oxidation [15]–[18]. The model is limited to the β-oxidation of saturated FAs containing an even number of carbon atoms. It starts from the transport of palmitoyl CoA across the mitochondrial inner membrane by the carnitine shuttle. The shuttle is reversible and also catalyzes the export of acyl-CoA esters of other chain lengths, which can be released as acyl carnitines in the extramitochondrial space. For simplicity we assumed that CPT1 activity is restricted to palmitoyl CoA (C16). Once inside the mitochondrial matrix, acyl-CoA molecules are oxidized to enoyl CoA by a set of chain-length-specific, FAD-dependent acyl-CoA dehydrogenases (SCAD, MCAD, LCAD and VLCAD in Figure 1) [15], [17]. The resulting enoyl CoAs undergo a sequential hydratase, dehydrogenase and thiolase reaction, leading to the formation of an NADH, an acetyl CoA and an acyl CoA which is two C-atoms shorter than the substrate of the cycle. This sequence of reactions is either catalyzed by the MTP (the right-hand branch in Figure 1) [19] or by three different enzymes (the left hand ‘crotonase’ branch in Figure 1) [20]–[25]. We assume that MTP does not release the hydroxyacyl-CoA and ketoacyl-CoA intermediates in the matrix, but channels them from one active site to another. In contrast, the intermediates in the ‘crotonase’ branch can diffuse freely through the matrix. There is overlap between the substrates that can be converted by the two pathways. However, MTP has a preference for longer chain lengths, while C4 and C6 substrates are uniquely converted by the crotonase branch.

The reaction scheme in Figure 1 is translated into a set of 45 ODEs describing how the time derivatives of the concentrations of the variable metabolites depend on the enzyme rates. For instance:
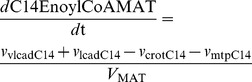
(1)denotes that the rate of change of the enoyl-CoA concentration of chain length C14 in the mitochondrial matrix equals the rate *v* of formation by VLCAD and LCAD minus the rate of consumption by crotonase and MTP. V_MAT_ denotes the volume of the mitochondrial matrix. In equation 1 each reaction rate is specific for a certain combination of enzyme and substrate, since most enzymes catalyze multiple reactions. The competing substrates inhibit each other's conversion competitively. This leads for instance to the following set of rate equation for crotonase:
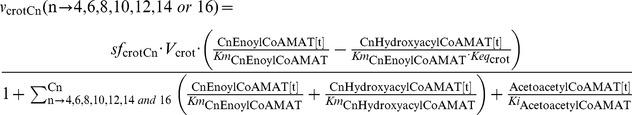
(2)


In the above ODE (equation 1) n = 14 for conversion of C14 and therefore the parameters for C14 are filled in, except in the Σ-term which sums over all substrates and products that are converted by crotonase and thus bind the enzyme reversibly. All of the reactions are modeled as reversible, mostly of Michaelis-Menten type as above, and most parameters were taken from the literature and mainly based on biochemical characterization of purified rat-liver enzymes. Some affinity and equilibrium constants were taken from rat-heart datasets. However, for enzymes of which only one isoenzyme exists, the affinity constants may be considered to be tissue-independent. Equilibrium constants do not depend at all on the enzyme that catalyzes the reaction. Three parameters, *V*_cpt1_, *V*_vlcad_ and *V*_lcad_, were estimated, since we could not find a reliable value in literature.

Three conserved moieties follow from the pathway stoichiometry: the total of FAD, NAD^+^ and CoA species. The model fulfills the criterion of microscopic reversibility, *i.e.* alternative reaction paths have the same overall equilibrium constant. For instance, the overall equilibrium constant of the MTP reaction equals the product of the equilibrium constants in the crotonase branch. We note that most equilibrium constants are not far from 1; hence the pathway lacks a strong thermodynamic driving force and depends on the delivery of substrate upstream and the further conversion of products downstream. A notable exception is the couple M/SCHAD – MCKAT, which has equilibrium constants of respectively 2.2·10^−4^ and 1.1·10^3^. This implies that M/SCHAD favors the reverse direction, which is compensated by the high equilibrium constant of MCKAT. MTP dampens this thermodynamic hurdle by keeping the intermediate ketoacyl CoA bound.

Based on the above principles, we constructed a model of 45 variable metabolite concentrations, 56 reactions and 234 parameters (for detailed description see Text S1), which predicts fluxes and metabolites in time and at steady state.

### Experimental validation of the model

The predicted model outcome was validated with experiments on isolated rat-liver mitochondria. We measured the oxygen-consumption flux and the acyl-carnitine concentrations (C4–C16) in time upon addition of palmitoyl CoA or palmitoyl carnitine. Mitochondria were incubated with an excess amount of malate. In this way CoASH could be regenerated from acetyl CoA by malate dehydrogenase and citrate synthase, allowing the β-oxidation to proceed. Each cycle of β-oxidation in the presence of excess malate gives rise to 1 FADH_2_ by acyl-CoA dehydrogenase and 2 NADH by enoyl-CoA dehydrogenase and malate dehydrogenase. The oxygen flux due to the β-oxidation *per se* was obtained by correcting the measured oxygen consumption for the oxygen consumption due to malate dehydrogenase. That malate to citrate was the main source of NADH beyond the β-oxidation was confirmed by measuring citrate, which indeed accumulated over time. Over the entire time course of 24 min, there was no significant gap between the carbon consumed as palmitoyl carnitine and the recovered products: carbon consumed = −16 • ([*acyl carnitine_C16_*]_t24_ - [*acyl carnitine_C16_*]_t0_) = 328±89 µM (SD) and carbon produced  = 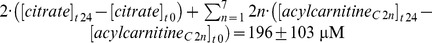 (SD); paired t-test p>0.05. Note that we did not include the carnitine moiety in the balance as it plays only a catalytic role. The same holds for the four carbons in citrate that are derived from malate. α-Ketoglutarate was not detectable. The uncertainty of the carbon balance leaves, however, room for other intermediates to contribute.

We determined the time course of palmitoyl carnitine and used this as an input function for the substrate concentration in the model. We assumed that the time course was similar for palmitoyl CoA when the latter was given as substrate (see Text S1).

**Figure 2:**
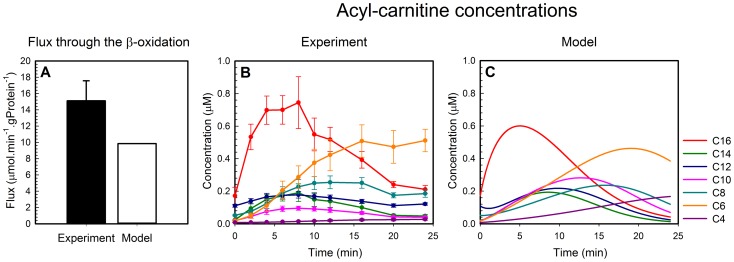
Comparison between the model simulations and the experiment with palmitoyl CoA as substrate. At time point zero the substrate palmitoyl CoA was added to uncoupled mitochondria in the presence of an excess amount of l-carnitine and malic acid. Samples for acyl carnitine analysis were taken at different time points. Error bars on the experimental data represent SEM (n = 4 for the flux data, n = 8 for the acyl-carnitine concentrations). Panel A: Comparison of the experimental and modeled flux through the FA β-oxidation. The reported experimental flux is 2/3 of the oxygen consumption flux averaged from 1.5 to 8 min. The modeled flux equals the production fluxes of NADH plus FADH_2_ divided by 2 (one O_2_ oxidizes two NADH or FADH_2_), averaged over the same time interval. Panel B: experimental acyl-carnitine concentrations in total samples, *i.e.* including intra- and extramitochondrial metabolites. Panel C: acyl-carnitine concentrations simulated by the computer model, representing the weighted average over the matrix and extramitochondrial concentrations to allow direct comparison to the experiments.

Within the experimental error the experimental and modeled flux corresponded well on both substrates (Figure 2A and Figure S1A). With palmitoyl carnitine, but not with palmitoyl CoA, a sharp initial overshoot was observed in the oxygen consumption flux, which was reproduced qualitatively by the model (Figure S1D–G). For the experiment with palmitoyl CoA as substrate, the dynamics of palmitoyl carnitine and the downstream intermediates myristoyl, octanoyl and hexanoyl carnitine (C14, C8 and C6) were quantitatively reproduced by the model. The remaining acyl carnitines (C10–C12 and C4) showed a *qualitative* correspondence between the experiment and model (Figure 2 and Figure S2A): the dynamics of the acyl carnitines were in all cases in the same direction, but the concentrations and/or timescale differed. For the experiment with palmitoyl carnitine as substrate, a *qualitative* correspondence was found, but the timescale and also concentrations differed (Figure S1B–C and S2B). We emphasize that the model parameters were not fitted to the experiments hence the correspondence between model and experiment is remarkably good.

### Perturbation of the system: Mimicking obesity

**Figure 3:**
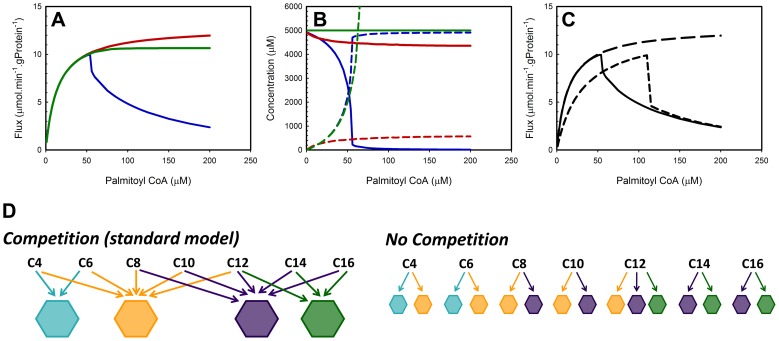
Steady-state fluxes and concentration of the intermediate metabolite at various concentrations of the substrate palmitoyl CoA. Panels A and C: Steady-state flux through the FA β-oxidation. Panel B: CoASH concentration (solid lines) and total CoA esters (dashed lines) corresponding to the simulations in A. Panel D: Graphical representation of the differences between the computational models with and without competition. Blue lines in A and B: standard model; red lines in A and B: model without competition; green lines in A and B: model with fixed CoASH concentration (see text). Solid line in C: standard model (malonyl-CoA concentration 0 µM, mitochondrial NAD/NADH ratio 15); short-dashed line in C: standard model but with increased concentration of malonyl CoA of 10 µM; long-dashed line in C: standard model but with increased mitochondrial NAD^+^/NADH ratio of 40. Different colors in panel D represent the four acyl-CoA dehydrogenases; cyan: SCAD, yellow: MCAD, purple: LCAD and green: VLCAD.

FA levels in plasma from obese people are often elevated [3], [8]. This means a constantly high availability of FAs for the β-oxidation. We have simulated this by calculating the steady-state flux and metabolite concentrations as a function of the concentration of palmitoyl CoA, the substrate in the model (Figure 3). The flux increased with an increasing concentration of palmitoyl CoA, but above 50 µM the flux decreased steeply, yet smoothly (Figure 3A, standard model). This was preceded by an accumulation of CoA esters (Figure 3B and Figure S3E) and consequently a decrease of CoASH (Figure 3B, standard model).

Most often a metabolic flux reaches a maximum at saturating substrate concentration, hence we considered the steep decline at high palmitoyl CoA an emergent, non-intuitive property of the β-oxidation. To prove that this property resulted from the competition between metabolites for a common set of enzymes, we adapted the model such that a percentage of each enzyme was dedicated to a particular chain length (‘No competition’ in Figure 3). This percentage was attributed such that at 25 µM of palmitoyl CoA the flux distribution among parallel enzymes was similar in both models (Figure S4). Indeed, in the model without competition the flux increased until saturation (Figure 3A), while the accumulation of CoA esters and the depletion of CoASH were prevented (Figure S3F and 3B).

*A priori* either the accumulation of the CoA esters or the depletion of CoASH could cause the decline of flux in the standard model. To distinguish between these possibilities we fixed the CoASH concentration in the standard model and left the CoA esters free to accumulate (Figure 3B), *i.e.* as if an external supply of CoASH had broken the moiety conservation. In this scenario the CoA esters accumulated extremely (Figure 3B and Figure S3G), but the flux nevertheless increased until saturation (Figure 3A, fixed CoASH). Accordingly, the drop in flux observed in the standard model is a result of CoASH depletion rather than of the high CoA-ester concentration.

Sensitivity analysis revealed that the influx of FAs into the FA β-oxidation by CPT1 and the consumption of the products of the enzyme M/SCHAD (NADH and ketoacyl CoAs in the mitochondrial matrix) had the largest effect on the CoASH concentration (Table S1). To explore the role of CPT1, we decreased its activity by adding the endogenous CPT1 inhibitor malonyl CoA or by lowering its *V_max_*. At low concentrations of palmitoyl CoA the flux was lower than in the standard model, but it rose to the same maximum and the drop in flux occurred at a higher palmitoyl-CoA concentration (Figure 3A and S3A). Accordingly, the accumulation of CoA esters and the depletion of CoASH still occurred, but at higher concentrations of palmitoyl CoA (Figure 3B and S3B, S3H–I). The influence of M/SCHAD is due to its very small equilibrium constant (2 · 10^−4^ [11], [26]) implying that the reaction can only work in the direction of the β-oxidation when its product concentrations are extremely low as compared to its substrate concentrations. To shift the M/SCHAD reaction in the forward direction, we increased the NAD/NADH ratio in the mitochondrial matrix from 15 to 40 without altering the sum of NAD and NADH. This prevented the flux decline at higher palmitoyl-CoA concentrations (Figure 3C and S3C). Accordingly, the concentrations of the CoA esters stayed low (Figure S3J) and a high CoASH concentration was maintained (Figure S3D).

## Discussion

In this paper we present the first dynamic model of the FA β-oxidation that appreciates the complex biochemical interactions in the network. Notably, we included the extensive competition in the system, as well as the qualitatively different pathways for conversion of enoyl CoAs. The parameters were based on biochemical analysis of individual enzymes and not fitted to obtain the desired metabolite and flux profiles. In this light, the correspondence between model predictions and experimental observations (Figure 2) was remarkably good. This allowed us to further explore the properties of the pathway.

We found that the unique pathway structure makes the FA β-oxidation vulnerable to substrate overload: at high palmitoyl-CoA concentrations the shorter CoA esters accumulate to outcompete the palmitoyl CoA. Above a critical palmitoyl-CoA concentration this results in depletion of CoASH and a steep decline in flux. This is an example of an ‘emergent’ property: it would not have occurred in a linear pathway without competition. Hence, it could not be predicted from the properties of the individual enzymes, but resulted from the wiring of the entire network. It is tempting to speculate that this overload phenotype is at the basis of various diseases and may be one of the mechanisms of lipotoxicity. We emphasize, however, that we modeled the FA β-oxidation in isolation. In reality, surrounding pathways may protect the pathway from overload. In addition, the model revealed two possible protection mechanisms at the pathway boundaries. In the following we will discuss these protective mechanisms as well the possible role of fatty-acid overload in insulin resistance.

First, according to the model the flux decline could be prevented by decreasing the activity of CPT1. Accordingly, increased concentrations of the CPT1 inhibitor malonyl CoA as well as a decreased catalytic capacity of CPT1 have been observed experimentally [27]–[32]. The most convincing data are for malonyl CoA, which is increased in skeletal muscle of obese humans and rodents [27], [28], [31], [32] as well as in liver tissue from obese mice [27]. Instead of being a *cause* of obesity via decreased FA oxidation and increased synthesis, malonyl CoA may rather confer *protection* against overload of the β-oxidation.

A second mechanism to prevent overload was to keep the products of the thermodynamically unfavorable M/SCHAD reaction low. In the parallel MTP pathway the *long-chain* hydroxyacyl-CoA (LCHAD) dehydrogenase is linked to the preceding long-chain enoyl-CoA hydratase and the following long-chain ketoacyl-CoA thiolase at the inner mitochondrial membrane [19], [23], [33]. Since the intermediate CoA esters are not detectable, it has been proposed that they are directly channeled from one active site to another [19], [23], [33]–[37]. The overall equilibrium constant of the lumped MTP reaction is 0.7, dampening the thermodynamic hurdle at LCHAD. Channeling has not been described for the short-chain intermediates (C4 and C6) and therefore we modeled their conversion by a sequence of enzymes (the ‘crotonase branch’) including the M/SCHAD reaction. The impact of the thermodynamic hurdle at M/SCHAD is demonstrated by the fact that the long-chain intermediates have a strong preference for the MTP branch, even though they could in principle be converted by the crotonase branch (Figure S4). Only C4 and C6 substrates, which are not recognized by MTP, take the crotonase route. Due to the low equilibrium constant of M/SCHAD (*K_eq_* = 2×10^−4^), the C6 and C4 intermediates accumulate in the model as well as in the experiment (Figure 2). A high mitochondrial NAD^+^/NADH ratio shifts the M/SCHAD equilibrium in the forward direction, prevents accumulation of the short-chain intermediates and eventually protects against the overload phenotype (Figure 3). This provides a functional explanation for the co-existence of M/SCHAD and Complex I in a respiratory supercomplex [38], [39]: locally, the NADH produced by M/SCHAD may be kept low by channeling it directly to complex I. In agreement with this hypothesis, the respiration-linked β-oxidation rate in gently-disrupted mitochondria (assuming an intact complex between M/SCHAD and Complex I) was much higher than in completely-disrupted mitochondria [40].

Besides the protection mechanisms found in the model, alternative mechanisms might be provided by surrounding pathways. Limited formation of palmitoyl CoA, either by inhibition of the synthetase reaction or due to a low cytosolic CoA concentration, would be very effective. Little is known however, about regulation of palmitoyl CoA synthesis. Another option is upregulation of the pathways that consume the product acetyl CoA, such as the Krebs cycle or the formation of ketone bodies. Indeed, the liver can produce high amounts of ketone bodies when confronted with a high fat load [41]. According to the model, a decreased acetyl-CoA concentration (K1acesink in the parameter list) should increase the concentration of CoASH (Table S1), although not as strongly as a decrease of the mitochondrial NADH concentration.

So far, direct experimental evidence for the overload phenotype is lacking. It is not unlikely, however, that it is at the basis of the well-known association of high FA levels to insulin resistance. Chronic exposure of muscle to elevated lipid levels results in an increased expression of FA β-oxidation genes, but this is not accompanied by an upregulation of downstream metabolic pathways, such as the TCA cycle and electron transport chain. In line with our model, it has been reported that this results in incomplete oxidation of fatty acids and accumulation of acyl carnitines and ketone bodies (reviewed in [41]). The fact that CPT1 is required to confer insulin resistance, suggests that the accumulated intermediate metabolites of the FA β-oxidation and/or ketone bodies may be involved in insulin resistance [6]. It is unclear, however, if the CoA esters or the carnitine esters are responsible for this effect, or both. Administration of l-carnitine, which is the ‘scavenger’ of CoA esters, sometimes restores glucose tolerance, in rats as well as humans [7], [42]. It is tempting to speculate that carnitine protects by trans-esterification of CoA esters to carnitine esters, which liberates CoASH and prevents accumulation of intermediates. *In vivo* it is not clear, however, if carnitine prevents overload, since it plays a dual role: in the mitochondrial matrix it scavenges intermediate CoA esters, but in the cytosol it drives the uptake of acyl CoAs into the mitochondria.

Since glucose and FA oxidation share the mitochondrial cofactors NAD^+^/NADH and CoASH, it may be expected that the presence of glucose will make the β-oxidation even more susceptible to overload. *Vice versa* a severe drop in CoASH will also compromise glucose oxidation via the TCA cycle. To further understand the interplay between glucose and FA metabolism and the quantitative role of various protective pathways, it will be of key interest to link the new model with (partially) existing models of glucose metabolism, TCA cycle, respiration, ketone-body synthesis and FA synthesis [43]–[46]. This should be the next step in elucidating the mechanisms behind acquired and inborn diseases of glucose and FA metabolism.

## Materials and Methods

### Ethics statement

Experimental procedures were approved by the Ethics Committees for Animal Experiments of the University of Groningen.

### Computational methods

The computational model was built and analyzed in Mathematica Wolfram. It consists of a set of Ordinary Differential Equations (ODEs). Time simulations were done with the algorithm NDSolve. Steady states were calculated by setting all time derivatives to zero and solving the resulting set of non-linear equations with the algorithm FindRoot. FindRoot is a root-finding algorithm combining damped Newton's method, the secant method and Brent's method. The solutions fulfilled the criterion that all time derivatives of metabolite concentrations approached zero (<10^−11^). We have no indications for alternative steady-state solutions, since different initial conditions led to identical steady states. As an input for the steady-state root-finding algorithm we used the endpoint of a time simulation. A Mathematica script and the corresponding pdf is added to this paper (Protocol S1 (steady state) and Protocol S2 (time simulation), as well as a detailed model description in Text S1). The models will become publically available at JWS Online Cellular Systems Modeling (jjj.bio.vu.nl).

### Flux and metabolite concentrations

Mitochondria were isolated from liver tissue of adult female Wistar rats (250–300 gram) according to Mildaziene [47]. The oxygen consumption rate of uncoupled mitochondria was measured with either palmitoyl CoA or palmitoyl carnitine as substrate, in the presence of ADP at 37°C in a stirred, two-channel high-resolution Oroboros oxygraph-2 k (Oroboros, Innsbruck, Austria). All measurements were done in 2 ml of MiR05 medium [48] to which 0.5 mg/ml mitochondrial protein, 0.2 µM FCCP (uncoupler), 2 mM malic acid, 500 µM l-carnitine, 1 mM ADP and 25 µM of either palmitoyl CoA or palmitoyl carnitine were added.

In parallel, 15–20 ml of the same reaction mixture was analyzed in a stirred, open vessel from which samples were taken in time for acyl-carnitine concentrations and TCA-cycle intermediates. Samples of 1.5 ml each were quenched by adding HCl to a final concentration of 0.4 M.

### Mass spectrometry analysis of acyl carnitines and TCA cycle intermediates

The determination of the acyl-carnitine concentrations was done according to Derks *et al.* [49]. The TCA-cycle intermediates are measured according to [50].

## Supporting Information

Figure S1 **Comparison between the model results and experimental data.** **Panel A–C:** model compared to the data of the experiment with palmitoyl carnitine as substrate. Error bars on the experimental data represent SEM (n = 4 for the flux data, n = 8 for the acyl carnitine concentrations). The reported experimental flux is 2/3 of the oxygen consumption flux averaged from 1.5 to 8 min. The modeled flux equals the production fluxes of NADH plus FADH_2_ divided by 2 (one O_2_ oxidizes 2 NADH or FADH_2_), averaged over the same time interval. **Panel D–G:** Comparison of the flux dynamics over time between model and experiment with either palmitoyl-CoA or palmitoyl carnitine as substrate for isolated rat-liver mitochondria. Dashed lines in the graphs from the experiment represent SEM (n = 4).
(PDF)

Figure S2 **Comparison of the acylcarnitine concentrations between model and experiment.** **Panel A:** palmitoyl CoA as substrate. Experimental values are depicted at the x-axis and the model values at the y-axis. Experimental concentration is the total concentration of intra and extramitochondrial acyl carnitine. Error bars indicate the SEM of the experimental data (n = 8). The black line represents a perfect match between the model and experimental values. **Panel B:** palmitoylcarnitine as substrate. Exeperimental values are depicted at the x-axis and the model values at the y-axis. Experimental concentration is the total concentration of intra and extramitochondrial acyl carnitine. Error bars indicate the SEM of the experimental data (n = 8). The black line represents a perfect match between the model and experimental values.
(PDF)

Figure S3 **Steady-state flux through the FA β-oxidation (A and C), CoASH concentration (B and D) and concentrations of the CoA esters (E–J) from the various model calculations.** **Panel A–D:** Steady-state flux through the FA β-oxidation (A and C) and CoASH concentration (B and D) at various concentrations of the substrate palmitoyl CoA. In the standard model the malonyl CoA concentration was 0 µM and the mitochondrial NAD/NADH ratio was 15. **Panel E–J:** For each CoA ester the concentrations of the specific CoA esters with different chain length were summed up. Panel E corresponds to Figure 3A and B (blue lines) in the main text; Panel F corresponds to Figure 3A and B (red lines) in the main text; Panel G corresponds to Figure 3A and B (green lines) in the main text; Panel H corresponds to Figure 3C (short-dashed lines) in the main text and Figure S3A and B (long-dashed lines); Panel I corresponds to Figure S3A and B (short-dashed lines); Panel J corresponds to Figure 3C (long-dashed lines) in the main text and Figure S3C and D (short-dashed lines).
(PDF)

Figure S4 **Flux distribution through the individual enzymes for the standard model (black bars) and the model without competition (white bars) at a palmitoyl-CoA concentration of 25 µM.** This figure corresponds to Figure 3A and B (blue lines) in the main text.
(PDF)

Protocol S1 **Steady-state model of the FA β-oxidation.**
(PDF)

Protocol S2 **Time-simulation model of the FA β-oxidation.**
(PDF)

Table S1
**Sensitivity analysis of the standard model.** Response coefficients of the concentration of CoASH towards the model parameters *p*. The response coefficient 
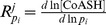
 was approximated by increasing the parameter of interest by 1%. Response coefficients are ranked from the highest to the lowest absolute value (positive coefficients in the left-hand column; negative coefficients in the right-hand column). Response coefficients were calculated for the steady-state standard model with 25 µM palmitoyl CoA as substrate.(PDF)

Text S1 **Model description.**
(PDF)
