# Supplementary material for: Biochemical Competition Makes Fatty-Acid β-Oxidation Vulnerable to Substrate Overload
Source: PLoS Comput Biol. 2013 Aug 15;9(8):e1003186. doi: 10.1371/journal.pcbi.1003186 (PMC3744394; doi:10.1371/journal.pcbi.1003186)

Figure S1: Comparison between the model results and experimental data

Panel A-C: model compared to the data of the experiment with palmitoyl carnitine as substrate. Error bars on the experimental data represent SEM (n=4 for the flux data, n=8 for the acyl carnitine concentrations). The reported experimental flux is 2/3 of the oxygen consumption flux averaged from 1.5 to 8 min. The modeled flux equals the production fluxes of NADH plus $FADH_2$ divided by 2 (one O_2 oxidizes 2 NADH or $FADH_2$), averaged over the same time interval.

Panel D-G: Comparison of the flux dynamics over time between model and experiment with either palmitoyl-CoA or palmitoyl carnitine as substrate for isolated rat-liver mitochondria. Dashed lines in the graphs from the experiment represent SEM (n=4).

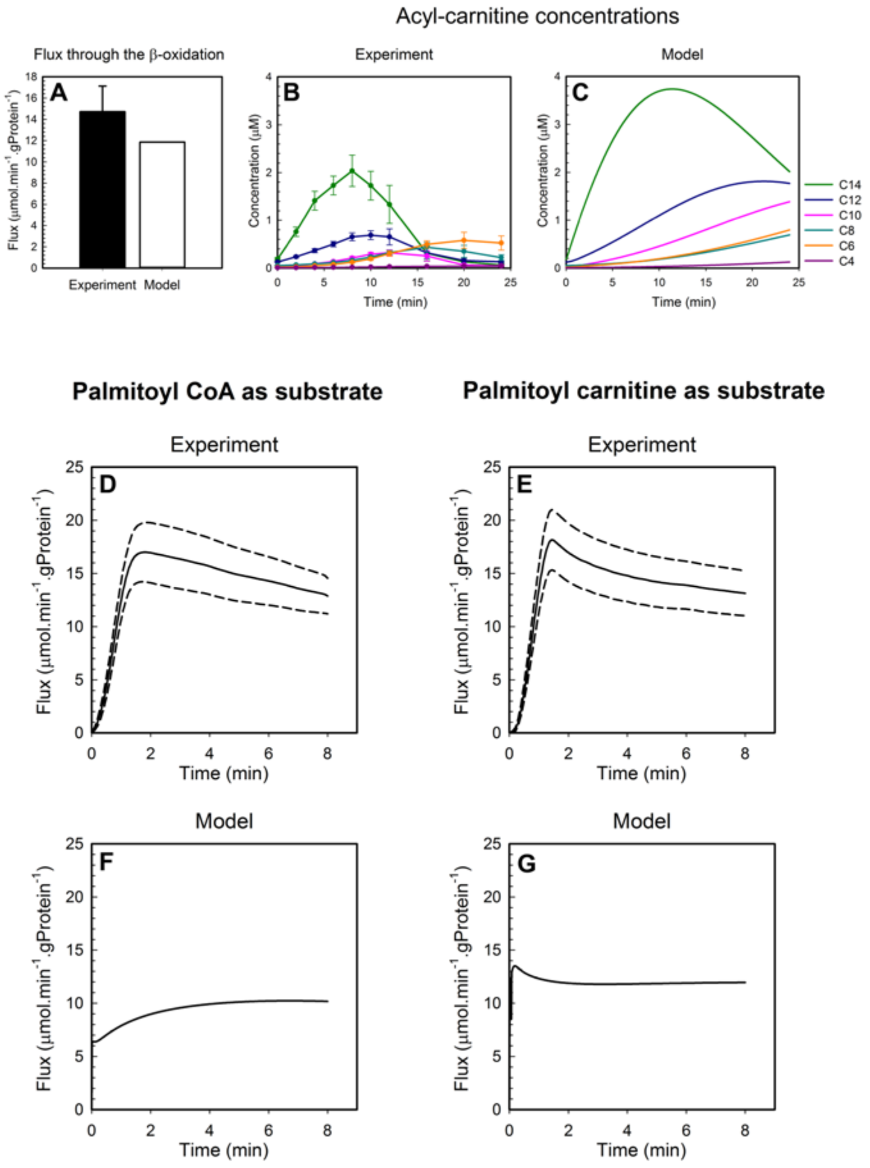

Supplement: Figure S1 — Comparison between the model results and experimental data. Panel A–C: model compared to the data of the experiment with palmitoyl carnitine as substrate. Error bars on the experimental data represent SEM (n = 4 for the flux data, n = 8 for the acyl carnitine concentrations). The reported experimental flux is 2/3 of the oxygen consumption flux averaged from 1.5 to 8 min. The modeled flux equals the production fluxes of NADH plus FADH2 divided by 2 (one O2 oxidizes 2 NADH or FADH2), averaged over the same time interval. Panel D–G: Comparison of the flux dynamics over time between model and experiment with either palmitoyl-CoA or palmitoyl carnitine as substrate for isolated rat-liver mitochondria. Dashed lines in the graphs from the experiment represent SEM (n = 4). (PDF) [file pcbi.1003186.s001.pdf]
