# Supplementary material for: Biochemical Competition Makes Fatty-Acid β-Oxidation Vulnerable to Substrate Overload
Source: PLoS Comput Biol. 2013 Aug 15;9(8):e1003186. doi: 10.1371/journal.pcbi.1003186 (PMC3744394; doi:10.1371/journal.pcbi.1003186)

Figure S2: Comparison of the acylcarnitine concentrations between model and experiment

Panel A: palmitoyl CoA as substrate. Experimental values are depicted at the x-axis and the model values at the y-axis. Experimental concentration is the total concentration of intra and extramitochondrial acyl carnitine. Error bars indicate the SEM of the experimental data (n=8). The black line represents a perfect match between the model and experimental values.

Panel B: palmitoylcarnitine as substrate. Exeperimental values are depicted at the x-axis and the model values at the y-axis. Experimental concentration is the total concentration of intra and extramitochondrial acyl carnitine. Error bars indicate the SEM of the experimental data (n=8). The black line represents a perfect match between the model and experimental values.

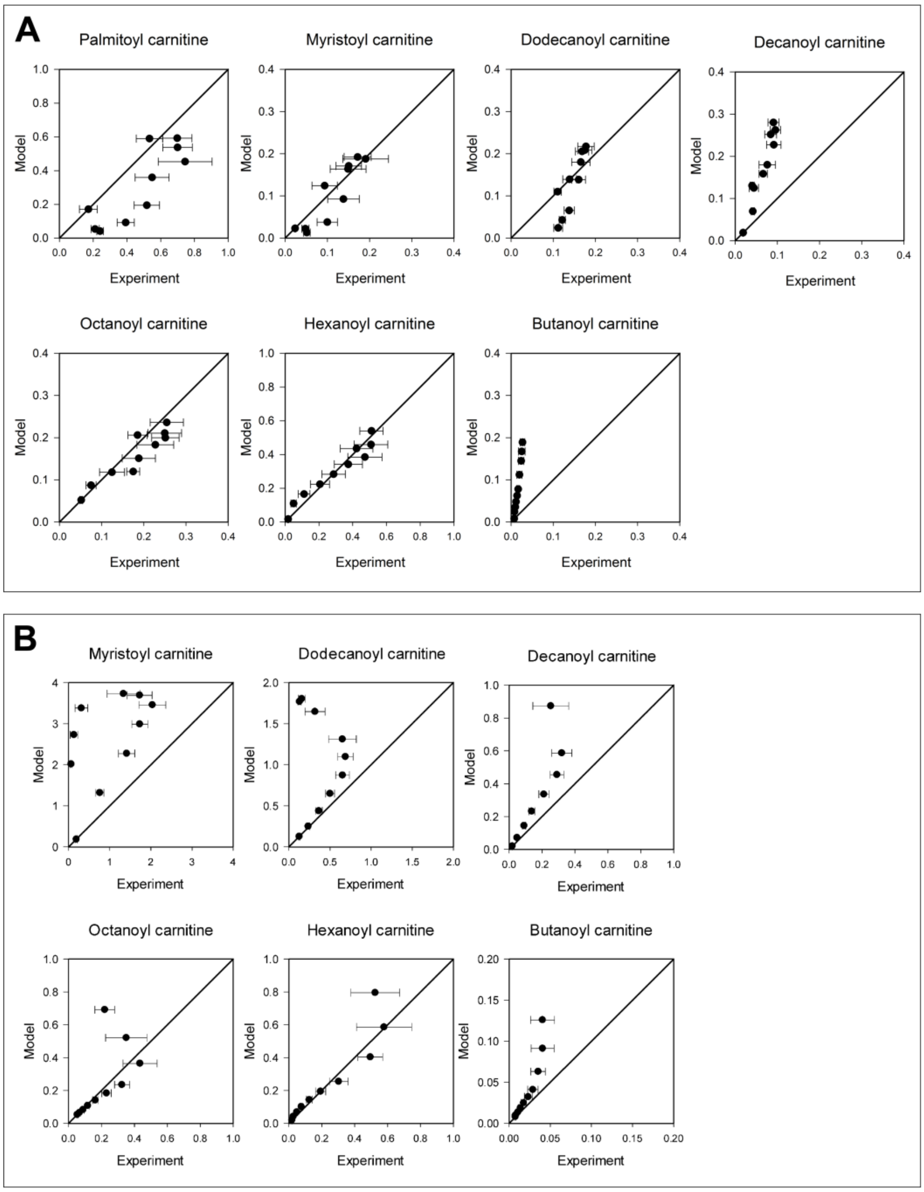

Supplement: Figure S2 — Comparison of the acylcarnitine concentrations between model and experiment. Panel A: palmitoyl CoA as substrate. Experimental values are depicted at the x-axis and the model values at the y-axis. Experimental concentration is the total concentration of intra and extramitochondrial acyl carnitine. Error bars indicate the SEM of the experimental data (n = 8). The black line represents a perfect match between the model and experimental values. Panel B: palmitoylcarnitine as substrate. Exeperimental values are depicted at the x-axis and the model values at the y-axis. Experimental concentration is the total concentration of intra and extramitochondrial acyl carnitine. Error bars indicate the SEM of the experimental data (n = 8). The black line represents a perfect match between the model and experimental values. (PDF) [file pcbi.1003186.s002.pdf]
