# Supplementary material for: Biochemical Competition Makes Fatty-Acid β-Oxidation Vulnerable to Substrate Overload
Source: PLoS Comput Biol. 2013 Aug 15;9(8):e1003186. doi: 10.1371/journal.pcbi.1003186 (PMC3744394; doi:10.1371/journal.pcbi.1003186)

Figure S3. Steady-state flux through the FA β-oxidation (A and C), CoASH concentration (B and D) and concentrations of the CoA esters (E-J) from the various model calculations.

Panel A-D: Steady-state flux through the FA β-oxidation (A and C) and CoASH concentration (B and D) at various concentrations of the substrate palmitoyl CoA. In the standard model the malonyl CoA concentration was 0 μM and the mitochondrial NAD/NADH ratio was 15.

Panel E-J: For each CoA ester the concentrations of the specific CoA esters with different chain length were summed up. Panel E corresponds to Figure 3A and B (blue lines) in the main text; Panel F corresponds to Figure 3A and B (red lines) in the main text; Panel G corresponds to Figure 3A and B (green lines) in the main text; Panel H corresponds to Figure 3C (short-dashed lines) in the main text and Figure S3A and B (long-dashed lines); Panel I corresponds to Figure S3A and B (short-dashed lines); Panel J corresponds to Figure 3C (long-dashed lines) in the main text and Figure S3C and D (short-dashed lines).

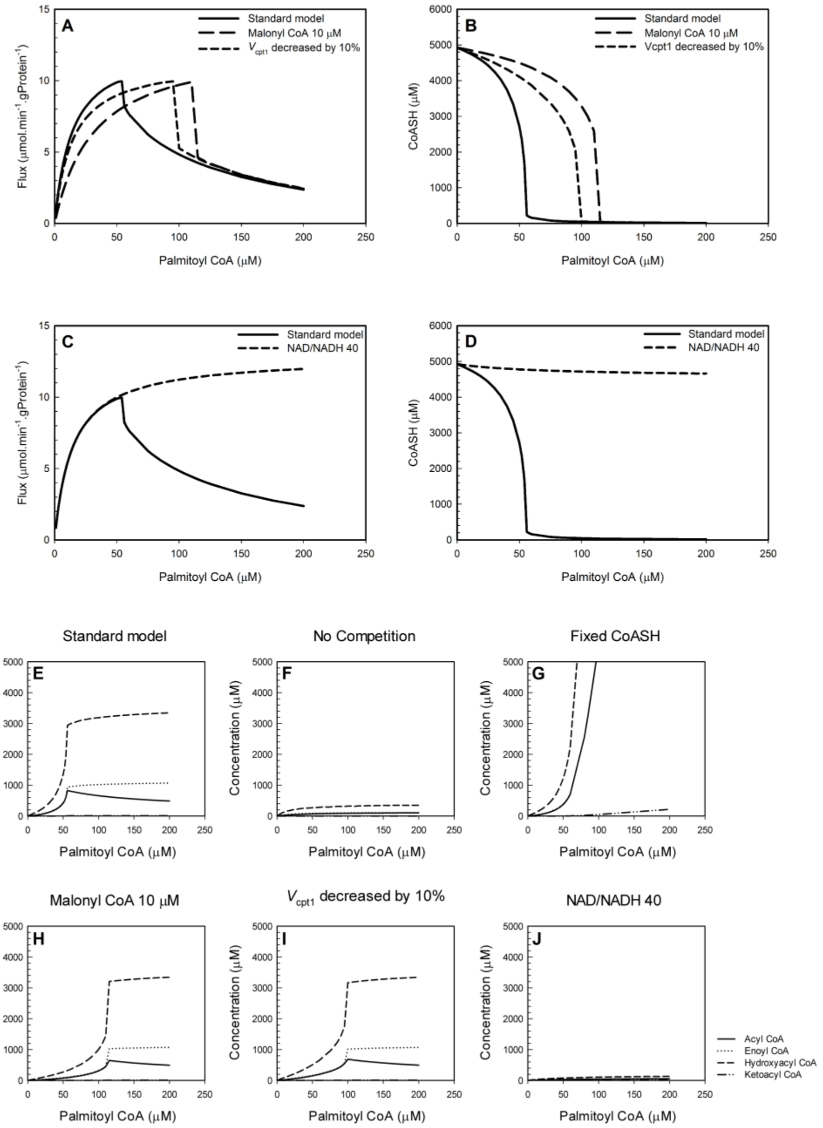

Supplement: Figure S3 — Steady-state flux through the FA β-oxidation (A and C), CoASH concentration (B and D) and concentrations of the CoA esters (E–J) from the various model calculations. Panel A–D: Steady-state flux through the FA β-oxidation (A and C) and CoASH concentration (B and D) at various concentrations of the substrate palmitoyl CoA. In the standard model the malonyl CoA concentration was 0 µM and the mitochondrial NAD/NADH ratio was 15. Panel E–J: For each CoA ester the concentrations of the specific CoA esters with different chain length were summed up. Panel E corresponds to Figure 3A and B (blue lines) in the main text; Panel F corresponds to Figure 3A and B (red lines) in the main text; Panel G corresponds to Figure 3A and B (green lines) in the main text; Panel H corresponds to Figure 3C (short-dashed lines) in the main text and Figure S3A and B (long-dashed lines); Panel I corresponds to Figure S3A and B (short-dashed lines); Panel J corresponds to Figure 3C (long-dashed lines) in the main text and Figure S3C and D (short-dashed lines). (PDF) [file pcbi.1003186.s003.pdf]
