# Supplementary material for: Biochemical Competition Makes Fatty-Acid β-Oxidation Vulnerable to Substrate Overload
Source: PLoS Comput Biol. 2013 Aug 15;9(8):e1003186. doi: 10.1371/journal.pcbi.1003186 (PMC3744394; doi:10.1371/journal.pcbi.1003186)

Figure S4: Flux distribution through the individual enzymes for the standard model (black bars) and the model without competition (white bars) at a palmitoyl-CoA concentration of 25 µM. This figure corresponds to Figure 3A and B (blue lines) in the main text.

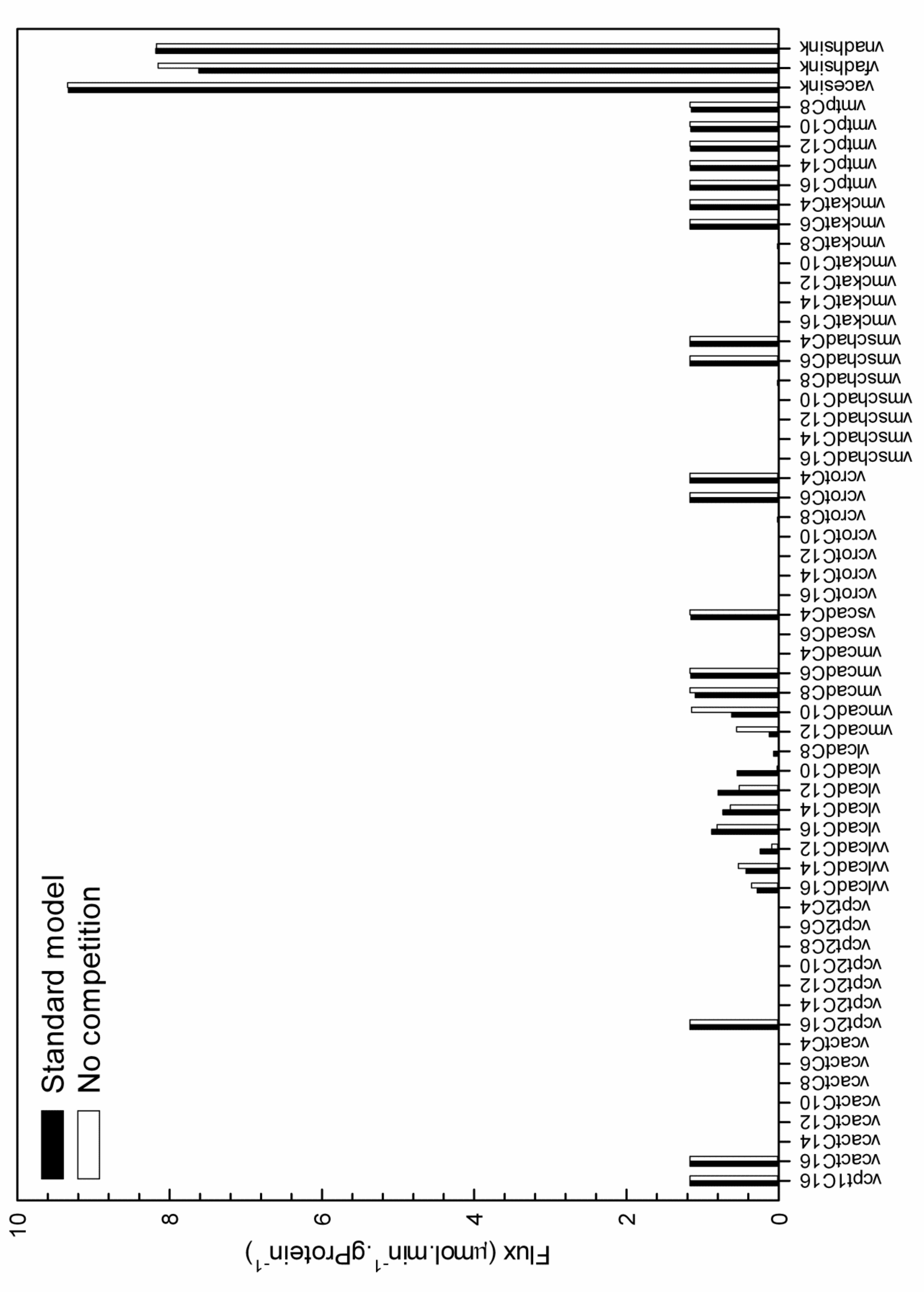

Supplement: Figure S4 — Flux distribution through the individual enzymes for the standard model (black bars) and the model without competition (white bars) at a palmitoyl-CoA concentration of 25 µM. This figure corresponds to Figure 3A and B (blue lines) in the main text. (PDF) [file pcbi.1003186.s004.pdf]
