# Supplementary material for: Biochemical Competition Makes Fatty-Acid β-Oxidation Vulnerable to Substrate Overload
Source: PLoS Comput Biol. 2013 Aug 15;9(8):e1003186. doi: 10.1371/journal.pcbi.1003186 (PMC3744394; doi:10.1371/journal.pcbi.1003186)
Supplement: Protocol S1 — Steady-state model of the FA β-oxidation. (PDF) [file pcbi.1003186.s005.pdf]

Protocol S1: Steady-state model of the FA β-oxidation.

Kinetic model

Definitions of the various functions

$$\mathrm{CPT1}[\mathrm{sf\_}, \mathrm{V\_}, \mathrm{Kms1\_}, \mathrm{Kms2\_}, \mathrm{Kmp1\_}, \mathrm{Kmp2\_}, \mathrm{Ki1\_}, \mathrm{Keq\_}, \mathrm{S1\_}, \mathrm{S2\_}, \mathrm{P1\_}, \mathrm{P2\_}, \mathrm{I1\_}, \mathrm{n\_}] := \frac{\mathrm{sf} * \mathrm{V} * \left(\frac{\mathrm{S1} * \mathrm{S2}}{\mathrm{Kms1} * \mathrm{Kms2}} - \frac{\mathrm{P1} * \mathrm{P2}}{\mathrm{Kms1} * \mathrm{Kms2} * \mathrm{Keq}}\right)}{\left(1 + \frac{\mathrm{S1}}{\mathrm{Kms1}} + \frac{\mathrm{P1}}{\mathrm{Kmp1}} + \left(\frac{\mathrm{I1}}{\mathrm{Ki1}}\right)^{\mathrm{n}}\right) * \left(1 + \frac{\mathrm{S2}}{\mathrm{Kms2}} + \frac{\mathrm{P2}}{\mathrm{Kmp2}}\right)}$$

$$\mathrm{CACT}[\mathrm{Vf\_}, \mathrm{Vr\_}, \mathrm{Kms1\_}, \mathrm{Kms2\_}, \mathrm{Kmp1\_}, \mathrm{Kmp2\_}, \mathrm{Kis1\_}, \mathrm{Kip2\_}, \mathrm{Keq\_}, \mathrm{S1\_}, \mathrm{S2\_}, \mathrm{P1\_}, \mathrm{P2\_}] := \left(\mathrm{Vf} * \left(\mathrm{S1} * \mathrm{S2} - \frac{\mathrm{P1} * \mathrm{P2}}{\mathrm{Keq}}\right)\right) \Big/ \left(\mathrm{S1} * \mathrm{S2} + \mathrm{Kms2} * \mathrm{S1} + \mathrm{Kms1} * \mathrm{S2} * \left(1 + \frac{\mathrm{P2}}{\mathrm{Kip2}}\right) + \frac{\mathrm{Vf}}{\mathrm{Vr} * \mathrm{Keq}} * \left(\mathrm{Kmp2} * \mathrm{P1} * \left(1 + \frac{\mathrm{S1}}{\mathrm{Kis1}}\right) + \mathrm{P2} * (\mathrm{Kmp1} + \mathrm{P1})\right)\right)$$

$$\mathrm{CPT2}[\mathrm{sf\_}, \mathrm{V\_}, \mathrm{Kms1\_}, \mathrm{Kms2\_}, \mathrm{Kms3\_}, \mathrm{Kms4\_}, \mathrm{Kms5\_}, \mathrm{Kms6\_}, \mathrm{Kms7\_}, \mathrm{Kms8\_}, \mathrm{Kmp1\_}, \mathrm{Kmp2\_}, \mathrm{Kmp3\_}, \mathrm{Kmp4\_}, \mathrm{Kmp5\_}, \mathrm{Kmp6\_}, \mathrm{Kmp7\_}, \mathrm{Kmp8\_}, \mathrm{Keq\_}, \mathrm{S1\_}, \mathrm{S2\_}, \mathrm{S3\_}, \mathrm{S4\_}, \mathrm{S5\_}, \mathrm{S6\_}, \mathrm{S7\_}, \mathrm{S8\_}, \mathrm{P1\_}, \mathrm{P2\_}, \mathrm{P3\_}, \mathrm{P4\_}, \mathrm{P5\_}, \mathrm{P6\_}, \mathrm{P7\_}, \mathrm{P8\_}] := \left(\mathrm{sf} * \mathrm{V} * \left(\frac{\mathrm{S1} * \mathrm{S8}}{\mathrm{Kms1} * \mathrm{Kms8}} - \frac{\mathrm{P1} * \mathrm{P8}}{\mathrm{Kms1} * \mathrm{Kms8} * \mathrm{Keq}}\right)\right) \Big/ \left(\left(1 + \frac{\mathrm{S1}}{\mathrm{Kms1}} + \frac{\mathrm{P1}}{\mathrm{Kmp1}} + \frac{\mathrm{S2}}{\mathrm{Kms2}} + \frac{\mathrm{P2}}{\mathrm{Kmp2}} + \frac{\mathrm{S3}}{\mathrm{Kms3}} + \frac{\mathrm{P3}}{\mathrm{Kmp3}} + \frac{\mathrm{S4}}{\mathrm{Kms4}} + \frac{\mathrm{P4}}{\mathrm{Kmp4}} + \frac{\mathrm{S5}}{\mathrm{Kms5}} + \frac{\mathrm{P5}}{\mathrm{Kmp5}} + \frac{\mathrm{S6}}{\mathrm{Kms6}} + \frac{\mathrm{P6}}{\mathrm{Kmp6}} + \frac{\mathrm{S7}}{\mathrm{Kms7}} + \frac{\mathrm{P7}}{\mathrm{Kmp7}}\right) * \left(1 + \frac{\mathrm{S8}}{\mathrm{Kms8}} + \frac{\mathrm{P8}}{\mathrm{Kmp8}}\right)\right)$$

$$\mathrm{VLCAD}[\mathrm{sf\_}, \mathrm{V\_}, \mathrm{Kms1\_}, \mathrm{Kms2\_}, \mathrm{Kms3\_}, \mathrm{Kms4\_}, \mathrm{Kmp1\_}, \mathrm{Kmp2\_}, \mathrm{Kmp3\_}, \mathrm{Kmp4\_}, \mathrm{Keq\_}, \mathrm{S1\_}, \mathrm{S2\_}, \mathrm{S3\_}, \mathrm{S4\_}, \mathrm{P1\_}, \mathrm{P2\_}, \mathrm{P3\_}, \mathrm{P4\_}] := \frac{\mathrm{sf} * \mathrm{V} * \left(\frac{\mathrm{S1} * (\mathrm{S4} - \mathrm{P4})}{\mathrm{Kms1} * \mathrm{Kms4}} - \frac{\mathrm{P1} * \mathrm{P4}}{\mathrm{Kms1} * \mathrm{Kms4} * \mathrm{Keq}}\right)}{\left(1 + \frac{\mathrm{S1}}{\mathrm{Kms1}} + \frac{\mathrm{P1}}{\mathrm{Kmp1}} + \frac{\mathrm{S2}}{\mathrm{Kms2}} + \frac{\mathrm{P2}}{\mathrm{Kmp2}} + \frac{\mathrm{S3}}{\mathrm{Kms3}} + \frac{\mathrm{P3}}{\mathrm{Kmp3}}\right) * \left(1 + \frac{(\mathrm{S4} - \mathrm{P4})}{\mathrm{Kms4}} + \frac{\mathrm{P4}}{\mathrm{Kmp4}}\right)}$$

$$\mathrm{LCAD}[\mathrm{sf\_}, \mathrm{V\_}, \mathrm{Kms1\_}, \mathrm{Kms2\_}, \mathrm{Kms3\_}, \mathrm{Kms4\_}, \mathrm{Kms5\_}, \mathrm{Kms6\_}, \mathrm{Kmp1\_}, \mathrm{Kmp2\_}, \mathrm{Kmp3\_}, \mathrm{Kmp4\_}, \mathrm{Kmp5\_}, \mathrm{Kmp6\_}, \mathrm{Keq\_}, \mathrm{S1\_}, \mathrm{S2\_}, \mathrm{S3\_}, \mathrm{S4\_}, \mathrm{S5\_}, \mathrm{S6\_}, \mathrm{P1\_}, \mathrm{P2\_}, \mathrm{P3\_}, \mathrm{P4\_}, \mathrm{P5\_}, \mathrm{P6\_}] := \left(\mathrm{sf} * \mathrm{V} * \left(\frac{\mathrm{S1} * (\mathrm{S6} - \mathrm{P6})}{\mathrm{Kms1} * \mathrm{Kms6}} - \frac{\mathrm{P1} * \mathrm{P6}}{\mathrm{Kms1} * \mathrm{Kms6} * \mathrm{Keq}}\right)\right) \Big/ \left(\left(1 + \frac{\mathrm{S1}}{\mathrm{Kms1}} + \frac{\mathrm{P1}}{\mathrm{Kmp1}} + \frac{\mathrm{S2}}{\mathrm{Kms2}} + \frac{\mathrm{P2}}{\mathrm{Kmp2}} + \frac{\mathrm{S3}}{\mathrm{Kms3}} + \frac{\mathrm{P3}}{\mathrm{Kmp3}} + \frac{\mathrm{S4}}{\mathrm{Kms4}} + \frac{\mathrm{P4}}{\mathrm{Kmp4}} + \frac{\mathrm{S5}}{\mathrm{Kms5}} + \frac{\mathrm{P5}}{\mathrm{Kmp5}}\right) * \left(1 + \frac{(\mathrm{S6} - \mathrm{P6})}{\mathrm{Kms6}} + \frac{\mathrm{P6}}{\mathrm{Kmp6}}\right)\right)$$

$$\text{MCAD}[\text{sf\_}, \text{V\_}, \text{Kms1\_}, \text{Kms2\_}, \text{Kms3\_}, \text{Kms4\_}, \text{Kms5\_}, \text{Kms6\_}, \text{Kmp1\_}, \text{Kmp2\_}, \text{Kmp3\_}, \text{Kmp4\_}, \text{Kmp5\_}, \text{Kmp6\_}, \text{Keq\_}, \text{S1\_}, \text{S2\_}, \text{S3\_}, \text{S4\_}, \text{S5\_}, \text{S6\_}, \text{P1\_}, \text{P2\_}, \text{P3\_}, \text{P4\_}, \text{P5\_}, \text{P6\_}] := \left(\text{sf} * \text{V} * \left(\frac{\text{S1} * (\text{S6} - \text{P6})}{\text{Kms1} * \text{Kms6}} - \frac{\text{P1} * \text{P6}}{\text{Kms1} * \text{Kms6} * \text{Keq}}\right)\right) \Big/ \left(\left(1 + \frac{\text{S1}}{\text{Kms1}} + \frac{\text{P1}}{\text{Kmp1}} + \frac{\text{S2}}{\text{Kms2}} + \frac{\text{P2}}{\text{Kmp2}} + \frac{\text{S3}}{\text{Kms3}} + \frac{\text{P3}}{\text{Kmp3}} + \frac{\text{S4}}{\text{Kms4}} + \frac{\text{P4}}{\text{Kmp4}} + \frac{\text{S5}}{\text{Kms5}} + \frac{\text{P5}}{\text{Kmp5}}\right) * \left(1 + \frac{(\text{S6} - \text{P6})}{\text{Kms6}} + \frac{\text{P6}}{\text{Kmp6}}\right)\right)$$

$$\text{SCAD}[\text{sf\_}, \text{V\_}, \text{Kms1\_}, \text{Kms2\_}, \text{Kms3\_}, \text{Kmp1\_}, \text{Kmp2\_}, \text{Kmp3\_}, \text{Keq\_}, \text{S1\_}, \text{S2\_}, \text{S3\_}, \text{P1\_}, \text{P2\_}, \text{P3\_}] := \frac{\text{sf} * \text{V} * \left(\frac{\text{S1}*(\text{S3}-\text{P3})}{\text{Kms1}*\text{Kms3}} - \frac{\text{P1}*\text{P3}}{\text{Kms1}*\text{Kms3}*\text{Keq}}\right)}{\left(1 + \frac{\text{S1}}{\text{Kms1}} + \frac{\text{P1}}{\text{Kmp1}} + \frac{\text{S2}}{\text{Kms2}} + \frac{\text{P2}}{\text{Kmp2}}\right) * \left(1 + \frac{(\text{S3}-\text{P3})}{\text{Kms3}} + \frac{\text{P3}}{\text{Kmp3}}\right)}$$

$$\text{CROT}[\text{sf\_}, \text{V\_}, \text{Kms1\_}, \text{Kms2\_}, \text{Kms3\_}, \text{Kms4\_}, \text{Kms5\_}, \text{Kms6\_}, \text{Kms7\_}, \text{Kmp1\_}, \text{Kmp2\_}, \text{Kmp3\_}, \text{Kmp4\_}, \text{Kmp5\_}, \text{Kmp6\_}, \text{Kmp7\_}, \text{Ki1\_}, \text{Keq\_}, \text{S1\_}, \text{S2\_}, \text{S3\_}, \text{S4\_}, \text{S5\_}, \text{S6\_}, \text{S7\_}, \text{P1\_}, \text{P2\_}, \text{P3\_}, \text{P4\_}, \text{P5\_}, \text{P6\_}, \text{P7\_}, \text{I1\_}] := \left(\text{sf} * \text{V} * \left(\frac{\text{S1}}{\text{Kms1}} - \frac{\text{P1}}{\text{Kms1} * \text{Keq}}\right)\right) \Big/ \left(1 + \frac{\text{S1}}{\text{Kms1}} + \frac{\text{P1}}{\text{Kmp1}} + \frac{\text{S2}}{\text{Kms2}} + \frac{\text{P2}}{\text{Kmp2}} + \frac{\text{S3}}{\text{Kms3}} + \frac{\text{P3}}{\text{Kmp3}} + \frac{\text{S4}}{\text{Kms4}} + \frac{\text{P4}}{\text{Kmp4}} + \frac{\text{S5}}{\text{Kms5}} + \frac{\text{P5}}{\text{Kmp5}} + \frac{\text{S6}}{\text{Kms6}} + \frac{\text{P6}}{\text{Kmp6}} + \frac{\text{S7}}{\text{Kms7}} + \frac{\text{P7}}{\text{Kmp7}} + \frac{\text{I1}}{\text{Ki1}}\right)$$

$$\text{MSCHAD}[\text{sf\_}, \text{V\_}, \text{Kms1\_}, \text{Kms2\_}, \text{Kms3\_}, \text{Kms4\_}, \text{Kms5\_}, \text{Kms6\_}, \text{Kms7\_}, \text{Kms8\_}, \text{Kmp1\_}, \text{Kmp2\_}, \text{Kmp3\_}, \text{Kmp4\_}, \text{Kmp5\_}, \text{Kmp6\_}, \text{Kmp7\_}, \text{Kmp8\_}, \text{Keq\_}, \text{S1\_}, \text{S2\_}, \text{S3\_}, \text{S4\_}, \text{S5\_}, \text{S6\_}, \text{S7\_}, \text{S8\_}, \text{P1\_}, \text{P2\_}, \text{P3\_}, \text{P4\_}, \text{P5\_}, \text{P6\_}, \text{P7\_}, \text{P8\_}] := \left(\text{sf} * \text{V} * \left(\frac{\text{S1} * (\text{S8} - \text{P8})}{\text{Kms1} * \text{Kms8}} - \frac{\text{P1} * \text{P8}}{\text{Kms1} * \text{Kms8} * \text{Keq}}\right)\right) \Big/ \left(\left(1 + \frac{\text{S1}}{\text{Kms1}} + \frac{\text{P1}}{\text{Kmp1}} + \frac{\text{S2}}{\text{Kms2}} + \frac{\text{P2}}{\text{Kmp2}} + \frac{\text{S3}}{\text{Kms3}} + \frac{\text{P3}}{\text{Kmp3}} + \frac{\text{S4}}{\text{Kms4}} + \frac{\text{P4}}{\text{Kmp4}} + \frac{\text{S5}}{\text{Kms5}} + \frac{\text{P5}}{\text{Kmp5}} + \frac{\text{S6}}{\text{Kms6}} + \frac{\text{P6}}{\text{Kmp6}} + \frac{\text{S7}}{\text{Kms7}} + \frac{\text{P7}}{\text{Kmp7}}\right) * \left(1 + \frac{(\text{S8} - \text{P8})}{\text{Kms8}} + \frac{\text{P8}}{\text{Kmp8}}\right)\right)$$

$$\text{MCKATA}[\text{sf\_}, \text{V\_}, \text{Kms1\_}, \text{Kms2\_}, \text{Kms3\_}, \text{Kms4\_}, \text{Kms5\_}, \text{Kms6\_}, \text{Kms7\_}, \text{Kms8\_}, \text{Kmp1\_}, \text{Kmp2\_}, \text{Kmp3\_}, \text{Kmp4\_}, \text{Kmp5\_}, \text{Kmp6\_}, \text{Kmp7\_}, \text{Kmp8\_}, \text{Keq\_}, \text{S1\_}, \text{S2\_}, \text{S3\_}, \text{S4\_}, \text{S5\_}, \text{S6\_}, \text{S7\_}, \text{S8\_}, \text{P1\_}, \text{P2\_}, \text{P3\_}, \text{P4\_}, \text{P5\_}, \text{P6\_}, \text{P7\_}, \text{P8\_}] := \left(\text{sf} * \text{V} * \left(\frac{\text{S1} * \text{S8}}{\text{Kms1} * \text{Kms8}} - \frac{\text{P1} * \text{P8}}{\text{Kms1} * \text{Kms8} * \text{Keq}}\right)\right) \Big/ \left(\left(1 + \frac{\text{S1}}{\text{Kms1}} + \frac{\text{P1}}{\text{Kmp1}} + \frac{\text{S2}}{\text{Kms2}} + \frac{\text{P2}}{\text{Kmp2}} + \frac{\text{S3}}{\text{Kms3}} + \frac{\text{P3}}{\text{Kmp3}} + \frac{\text{S4}}{\text{Kms4}} + \frac{\text{P4}}{\text{Kmp4}} + \frac{\text{S5}}{\text{Kms5}} + \frac{\text{P5}}{\text{Kmp5}} + \frac{\text{S6}}{\text{Kms6}} + \frac{\text{P6}}{\text{Kmp6}} + \frac{\text{S7}}{\text{Kms7}} + \frac{\text{P7}}{\text{Kmp7}} + \frac{\text{P8}}{\text{Kmp8}}\right) * \left(1 + \frac{\text{S8}}{\text{Kms8}} + \frac{\text{P8}}{\text{Kmp8}}\right)\right)$$

```
MCKATB[sf_, V_, Kms1_, Kms2_, Kms3_, Kms4_, Kms5_, Kms6_, Kms7_, Kms8_,
  Kmp1_, Kmp2_, Kmp3_, Kmp4_, Kmp5_, Kmp6_, Kmp7_, Kmp8_, Keq_, S1_, S2_,
  S3_, S4_, S5_, S6_, S7_, S8_, P1_, P2_, P3_, P4_, P5_, P6_, P7_, P8_] :=
```

$$\left(sf * V * \left(\frac{S1 * S8}{Kms1 * Kms8} - \frac{P8 * P8}{Kms1 * Kms8 * Keq}\right)\right) \Big/ \left(\left(1 + \frac{S1}{Kms1} + \frac{P1}{Kmp1} + \frac{S2}{Kms2} + \frac{P2}{Kmp2} + \frac{S3}{Kms3} + \frac{P3}{Kmp3} + \frac{S4}{Kms4} + \frac{P4}{Kmp4} + \frac{S5}{Kms5} + \frac{P5}{Kmp5} + \frac{S6}{Kms6} + \frac{P6}{Kmp6} + \frac{S7}{Kms7} + \frac{P7}{Kmp7} + \frac{P8}{Kmp8}\right) * \left(1 + \frac{S8}{Kms8} + \frac{P8}{Kmp8}\right)\right)$$

```
MTP[sf_, V_, Kms1_, Kms2_, Kms3_, Kms4_, Kms5_, Kms7_, Kms8_, Kmp1_,
  Kmp2_, Kmp3_, Kmp4_, Kmp5_, Kmp6_, Kmp7_, Kmp8_, Ki1_, Keq_, S1_, S2_,
  S3_, S4_, S5_, S7_, S8_, P1_, P2_, P3_, P4_, P5_, P6_, P7_, P8_, I1_] :=
```

$$\left(sf * V * \left(\frac{S1 * (S7 - P7) * S8}{Kms1 * Kms7 * Kms8} - \frac{P1 * P7 * P8}{Kms1 * Kms7 * Kms8 * Keq}\right)\right) \Big/ \left(\left(1 + \frac{S1}{Kms1} + \frac{P1}{Kmp1} + \frac{S2}{Kms2} + \frac{P2}{Kmp2} + \frac{S3}{Kms3} + \frac{P3}{Kmp3} + \frac{S4}{Kms4} + \frac{P4}{Kmp4} + \frac{S5}{Kms5} + \frac{P5}{Kmp5} + \frac{P6}{Kmp6} + \frac{I1}{Ki1}\right) * \left(1 + \frac{(S7 - P7)}{Kms7} + \frac{P7}{Kmp7}\right) * \left(1 + \frac{S8}{Kms8} + \frac{P8}{Kmp8}\right)\right)$$

```
RES[Ks_, S_, K1_] := Ks * (S - K1)
```

Define the differential equations

Odes = {

$$C16AcylCarCYT'[t] == \frac{vcpt1C16 - vcactC16}{VCYT},$$

$$C16AcylCarMAT'[t] == \frac{vcactC16 - vcpt2C16}{VMAT},$$

$$C16AcylCoAMAT'[t] == \frac{vcpt2C16 - vvlcadC16 - vlcadC16}{VMAT},$$

$$C16EnoylCoAMAT'[t] == \frac{vvlcadC16 + vlcadC16 - vcrotC16 - vmtpC16}{VMAT},$$

$$C16HydroxyacylCoAMAT'[t] == \frac{vcrotC16 - vmschadC16}{VMAT},$$

$$C16KetoacylCoAMAT'[t] == \frac{vmschadC16 - vmckatC16}{VMAT},$$

$$C14AcylCarCYT'[t] == \frac{-vcactC14}{VCYT},$$

$$C14AcylCarMAT'[t] == \frac{vcactC14 - vcpt2C14}{VMAT},$$

$$C14AcylCoAMAT'[t] == \frac{vcpt2C14 + vmtpC16 + vmckatC16 - vvlcadC14 - vlcadC14}{VMAT},$$

$$C14EnoylCoAMAT'[t] == \frac{vvlcadC14 + vlcadC14 - vcrotC14 - vmtpC14}{VMAT},$$

$$C14HydroxyacylCoAMAT'[t] == \frac{vcrotC14 - vmschadC14}{VMAT},$$

$$C14KetoacylCoAMAT'[t] == \frac{vmschadC14 - vmckatC14}{VMAT},$$

$$\text{C12AcylCarCYT}'[t] == \frac{-\text{vcactC12}}{\text{VCYT}},$$

$$\text{C12AcylCarMAT}'[t] == \frac{\text{vcactC12} - \text{vcpt2C12}}{\text{VMAT}},$$

$$\text{C12AcylCoAMAT}'[t] == \frac{1}{\text{VMAT}} (\text{vcpt2C12} + \text{vmtpC14} + \text{vmckatC14} - \text{vvlcadC12} - \text{vlcadC12} - \text{vmcadC12}),$$

$$\text{C12EnoylCoAMAT}'[t] == \frac{\text{vvlcadC12} + \text{vlcadC12} + \text{vmcadC12} - \text{vcrotC12} - \text{vmtpC12}}{\text{VMAT}},$$

$$\text{C12HydroxyacylCoAMAT}'[t] == \frac{\text{vcrotC12} - \text{vmschadC12}}{\text{VMAT}},$$

$$\text{C12KetoacylCoAMAT}'[t] == \frac{\text{vmschadC12} - \text{vmckatC12}}{\text{VMAT}},$$

$$\text{C10AcylCarCYT}'[t] == \frac{-\text{vcactC10}}{\text{VCYT}},$$

$$\text{C10AcylCarMAT}'[t] == \frac{\text{vcactC10} - \text{vcpt2C10}}{\text{VMAT}},$$

$$\text{C10AcylCoAMAT}'[t] == \frac{\text{vcpt2C10} + \text{vmtpC12} + \text{vmckatC12} - \text{vlcadC10} - \text{vmcadC10}}{\text{VMAT}},$$

$$\text{C10EnoylCoAMAT}'[t] == \frac{\text{vlcadC10} + \text{vmcadC10} - \text{vcrotC10} - \text{vmtpC10}}{\text{VMAT}},$$

$$\text{C10HydroxyacylCoAMAT}'[t] == \frac{\text{vcrotC10} - \text{vmschadC10}}{\text{VMAT}},$$

$$\text{C10KetoacylCoAMAT}'[t] == \frac{\text{vmschadC10} - \text{vmckatC10}}{\text{VMAT}},$$

$$\text{C8AcylCarCYT}'[t] == \frac{-\text{vcactC8}}{\text{VCYT}},$$

$$\text{C8AcylCarMAT}'[t] == \frac{\text{vcactC8} - \text{vcpt2C8}}{\text{VMAT}},$$

$$\text{C8AcylCoAMAT}'[t] == \frac{\text{vcpt2C8} + \text{vmtpC10} + \text{vmckatC10} - \text{vlcadC8} - \text{vmcadC8}}{\text{VMAT}},$$

$$\text{C8EnoylCoAMAT}'[t] == \frac{\text{vlcadC8} + \text{vmcadC8} - \text{vcrotC8} - \text{vmtpC8}}{\text{VMAT}},$$

$$\text{C8HydroxyacylCoAMAT}'[t] == \frac{\text{vcrotC8} - \text{vmschadC8}}{\text{VMAT}},$$

$$\text{C8KetoacylCoAMAT}'[t] == \frac{\text{vmschadC8} - \text{vmckatC8}}{\text{VMAT}},$$

$$\text{C6AcylCarCYT}'[t] == \frac{-\text{vcactC6}}{\text{VCYT}},$$

$$\text{C6AcylCarMAT}'[t] == \frac{\text{vcactC6} - \text{vcpt2C6}}{\text{VMAT}},$$

$$\text{C6AcylCoAMAT}'[t] == \frac{\text{vcpt2C6} + \text{vmtpC8} + \text{vmckatC8} - \text{vmcadC6} - \text{vscadC6}}{\text{VMAT}},$$

$$\text{C6EnoylCoAMAT}'[t] == \frac{\text{vmcadC6} + \text{vscadC6} - \text{vcrotC6}}{\text{VMAT}},$$

$$\text{C6HydroxyacylCoAMAT}'[t] == \frac{\text{vcrotC6} - \text{vmschadC6}}{\text{VMAT}},$$

$$\text{C6KetoacylCoAMAT}'[t] == \frac{\text{vmschadC6} - \text{vmckatC6}}{\text{VMAT}},$$

```
C4AcylCarCYT'[t] == -vcactC4 / VCYT,
C4AcylCarMAT'[t] == (vcactC4 - vcpt2C4) / VMAT,
C4AcylCoAMAT'[t] == (vcpt2C4 + vmckatC6 - vmcadC4 - vscadC4) / VMAT,
C4EnoylCoAMAT'[t] == (vmcadC4 + vscadC4 - vcrotC4) / VMAT,
C4HydroxyacylCoAMAT'[t] == (vcrotC4 - vmschadC4) / VMAT,
C4AcetoacylCoAMAT'[t] == (vmschadC4 - vmckatC4) / VMAT,
AcetylCoAMAT'[t] ==
  1/VMAT (vmtpC16 + vmckatC16 + vmtpC14 + vmckatC14 + vmtpC12 + vmckatC12 + vmtpC10 +
     vmckatC10 + vmtpC8 + vmckatC8 + vmckatC6 + 2 * vmckatC4 - vacesink),
FADHMAT'[t] == 1/VMAT (vvlcadC16 + vvlcadC14 + vvlcadC12 + vlcadC16 +
     vlcadC14 + vlcadC12 + vlcadC10 + vlcadC8 + vmcadC12 + vmcadC10 +
     vmcadC8 + vmcadC6 + vmcadC4 + vscadC6 + vscadC4 - vfadhsink),
NADHMAT'[t] == 1/VMAT (vmtpC16 + vmtpC14 + vmtpC12 + vmtpC10 + vmtpC8 +
     vmschadC16 + vmschadC14 + vmschadC12 + vmschadC10 +
     vmschadC8 + vmschadC6 + vmschadC4 - vnadhsink)};

RateEqs = {vcpt1C16 → CPT1[sfcpt1C16, Vcpt1, Kmcpt1C16AcylCoACYT, Kmcpt1CarCYT,
     Kmcpt1C16AcylCarCYT, Kmcpt1CoACYT, Kicpt1MalCoACYT, Keqcpt1,
     C16AcylCoACYT, CarCYT, C16AcylCarCYT[t], CoACYT, MalCoACYT, ncpt1],
   vcactC16 → CACT[Vfcact, Vrcact, KmcactC16AcylCarCYT, KmcactCarMAT,
     KmcactC16AcylCarMAT, KmcactCarCYT, KicactC16AcylCarCYT, KicactCarCYT,
     Keqcact, C16AcylCarCYT[t], CarMAT, C16AcylCarMAT[t], CarCYT],
   vcactC14 → CACT[Vfcact, Vrcact, KmcactC14AcylCarCYT, KmcactCarMAT,
     KmcactC14AcylCarMAT, KmcactCarCYT, KicactC14AcylCarCYT, KicactCarCYT,
     Keqcact, C14AcylCarCYT[t], CarMAT, C14AcylCarMAT[t], CarCYT],
   vcactC12 → CACT[Vfcact, Vrcact, KmcactC12AcylCarCYT, KmcactCarMAT,
     KmcactC12AcylCarMAT, KmcactCarCYT, KicactC12AcylCarCYT, KicactCarCYT,
     Keqcact, C12AcylCarCYT[t], CarMAT, C12AcylCarMAT[t], CarCYT],
   vcactC10 → CACT[Vfcact, Vrcact, KmcactC10AcylCarCYT, KmcactCarMAT,
     KmcactC10AcylCarMAT, KmcactCarCYT, KicactC10AcylCarCYT, KicactCarCYT,
     Keqcact, C10AcylCarCYT[t], CarMAT, C10AcylCarMAT[t], CarCYT],
   vcactC8 → CACT[Vfcact, Vrcact, KmcactC8AcylCarCYT, KmcactCarMAT,
     KmcactC8AcylCarMAT, KmcactCarCYT, KicactC8AcylCarCYT, KicactCarCYT,
     Keqcact, C8AcylCarCYT[t], CarMAT, C8AcylCarMAT[t], CarCYT],
   vcactC6 → CACT[Vfcact, Vrcact, KmcactC6AcylCarCYT, KmcactCarMAT,
     KmcactC6AcylCarMAT, KmcactCarCYT, KicactC6AcylCarCYT, KicactCarCYT,
     Keqcact, C6AcylCarCYT[t], CarMAT, C6AcylCarMAT[t], CarCYT],
   vcactC4 → CACT[Vfcact, Vrcact, KmcactC4AcylCarCYT, KmcactCarMAT,
     KmcactC4AcylCarMAT, KmcactCarCYT, KicactC4AcylCarCYT, KicactCarCYT,
     Keqcact, C4AcylCarCYT[t], CarMAT, C4AcylCarMAT[t], CarCYT],
   vcpt2C16 → CPT2[sfcpt2C16, Vcpt2, Kmcpt2C16AcylCarMAT, Kmcpt2C14AcylCarMAT,
     Kmcpt2C12AcylCarMAT, Kmcpt2C10AcylCarMAT, Kmcpt2C8AcylCarMAT,
```

```
   Kmcpt2C6AcylCarMAT, Kmcpt2C4AcylCarMAT, Kmcpt2CoAMAT,
   Kmcpt2C16AcylCoAMAT, Kmcpt2C14AcylCoAMAT, Kmcpt2C12AcylCoAMAT,
   Kmcpt2C10AcylCoAMAT, Kmcpt2C8AcylCoAMAT, Kmcpt2C6AcylCoAMAT,
   Kmcpt2C4AcylCoAMAT, Kmcpt2CarMAT, Keqcpt2, C16AcylCarMAT[t],
   C14AcylCarMAT[t], C12AcylCarMAT[t], C10AcylCarMAT[t], C8AcylCarMAT[t],
   C6AcylCarMAT[t], C4AcylCarMAT[t], CoAMAT, C16AcylCoAMAT[t],
   C14AcylCoAMAT[t], C12AcylCoAMAT[t], C10AcylCoAMAT[t],
   C8AcylCoAMAT[t], C6AcylCoAMAT[t], C4AcylCoAMAT[t], CarMAT],
vcpt2C14 → CPT2[sfcpt2C14, Vcpt2, Kmcpt2C14AcylCarMAT, Kmcpt2C16AcylCarMAT,
   Kmcpt2C12AcylCarMAT, Kmcpt2C10AcylCarMAT, Kmcpt2C8AcylCarMAT,
   Kmcpt2C6AcylCarMAT, Kmcpt2C4AcylCoAMAT, Kmcpt2CoAMAT,
   Kmcpt2C14AcylCoAMAT, Kmcpt2C16AcylCoAMAT, Kmcpt2C12AcylCoAMAT,
   Kmcpt2C10AcylCoAMAT, Kmcpt2C8AcylCoAMAT, Kmcpt2C6AcylCoAMAT,
   Kmcpt2C4AcylCoAMAT, Kmcpt2CarMAT, Keqcpt2, C14AcylCarMAT[t],
   C16AcylCarMAT[t], C12AcylCarMAT[t], C10AcylCarMAT[t], C8AcylCarMAT[t],
   C6AcylCarMAT[t], C4AcylCarMAT[t], CoAMAT, C14AcylCoAMAT[t],
   C16AcylCoAMAT[t], C12AcylCoAMAT[t], C10AcylCoAMAT[t],
   C8AcylCoAMAT[t], C6AcylCoAMAT[t], C4AcylCoAMAT[t], CarMAT],
vcpt2C12 → CPT2[sfcpt2C12, Vcpt2, Kmcpt2C12AcylCarMAT, Kmcpt2C16AcylCarMAT,
   Kmcpt2C14AcylCarMAT, Kmcpt2C10AcylCarMAT, Kmcpt2C8AcylCarMAT,
   Kmcpt2C6AcylCarMAT, Kmcpt2C4AcylCarMAT, Kmcpt2CoAMAT,
   Kmcpt2C12AcylCoAMAT, Kmcpt2C16AcylCoAMAT, Kmcpt2C14AcylCoAMAT,
   Kmcpt2C10AcylCoAMAT, Kmcpt2C8AcylCoAMAT, Kmcpt2C6AcylCoAMAT,
   Kmcpt2C4AcylCoAMAT, Kmcpt2CarMAT, Keqcpt2, C12AcylCarMAT[t],
   C16AcylCarMAT[t], C14AcylCarMAT[t], C10AcylCarMAT[t], C8AcylCarMAT[t],
   C6AcylCarMAT[t], C4AcylCarMAT[t], CoAMAT, C12AcylCoAMAT[t],
   C16AcylCoAMAT[t], C14AcylCoAMAT[t], C10AcylCoAMAT[t],
   C8AcylCoAMAT[t], C6AcylCoAMAT[t], C4AcylCoAMAT[t], CarMAT],
vcpt2C10 → CPT2[sfcpt2C10, Vcpt2, Kmcpt2C10AcylCarMAT, Kmcpt2C16AcylCarMAT,
   Kmcpt2C14AcylCarMAT, Kmcpt2C12AcylCarMAT, Kmcpt2C8AcylCarMAT,
   Kmcpt2C6AcylCarMAT, Kmcpt2C4AcylCarMAT, Kmcpt2CoAMAT,
   Kmcpt2C10AcylCoAMAT, Kmcpt2C16AcylCoAMAT, Kmcpt2C14AcylCoAMAT,
   Kmcpt2C12AcylCoAMAT, Kmcpt2C8AcylCoAMAT, Kmcpt2C6AcylCoAMAT,
   Kmcpt2C4AcylCoAMAT, Kmcpt2CarMAT, Keqcpt2, C10AcylCarMAT[t],
   C16AcylCarMAT[t], C14AcylCarMAT[t], C12AcylCarMAT[t], C8AcylCarMAT[t],
   C6AcylCarMAT[t], C4AcylCarMAT[t], CoAMAT, C10AcylCoAMAT[t],
   C16AcylCoAMAT[t], C14AcylCoAMAT[t], C12AcylCoAMAT[t],
   C8AcylCoAMAT[t], C6AcylCoAMAT[t], C4AcylCoAMAT[t], CarMAT],
vcpt2C8 → CPT2[sfcpt2C8, Vcpt2, Kmcpt2C8AcylCarMAT, Kmcpt2C16AcylCarMAT,
   Kmcpt2C14AcylCarMAT, Kmcpt2C12AcylCarMAT, Kmcpt2C10AcylCarMAT,
   Kmcpt2C6AcylCarMAT, Kmcpt2C4AcylCarMAT, Kmcpt2CoAMAT,
   Kmcpt2C8AcylCoAMAT, Kmcpt2C16AcylCoAMAT, Kmcpt2C14AcylCoAMAT,
   Kmcpt2C12AcylCoAMAT, Kmcpt2C10AcylCoAMAT, Kmcpt2C6AcylCoAMAT,
   Kmcpt2C4AcylCoAMAT, Kmcpt2CarMAT, Keqcpt2, C8AcylCarMAT[t],
   C16AcylCarMAT[t], C14AcylCarMAT[t], C12AcylCarMAT[t],
   C10AcylCarMAT[t], C6AcylCarMAT[t], C4AcylCarMAT[t], CoAMAT,
   C8AcylCoAMAT[t], C16AcylCoAMAT[t], C14AcylCoAMAT[t], C12AcylCoAMAT[t],
   C10AcylCoAMAT[t], C6AcylCoAMAT[t], C4AcylCoAMAT[t], CarMAT],
vcpt2C6 → CPT2[sfcpt2C6, Vcpt2, Kmcpt2C6AcylCarMAT, Kmcpt2C16AcylCarMAT,
   Kmcpt2C14AcylCarMAT, Kmcpt2C12AcylCarMAT, Kmcpt2C10AcylCarMAT,
   Kmcpt2C8AcylCarMAT, Kmcpt2C4AcylCarMAT, Kmcpt2CoAMAT,
   Kmcpt2C6AcylCoAMAT, Kmcpt2C16AcylCoAMAT, Kmcpt2C14AcylCoAMAT,
   Kmcpt2C12AcylCoAMAT, Kmcpt2C10AcylCoAMAT, Kmcpt2C8AcylCoAMAT,
```

```
    Kmcpt2C4AcylCoAMAT, Kmcpt2CarMAT, Keqcpt2, C6AcylCarMAT[t],
    C16AcylCarMAT[t], C14AcylCarMAT[t], C12AcylCarMAT[t],
    C10AcylCarMAT[t], C8AcylCarMAT[t], C4AcylCarMAT[t], CoAMAT,
    C6AcylCoAMAT[t], C16AcylCoAMAT[t], C14AcylCoAMAT[t], C12AcylCoAMAT[t],
    C10AcylCoAMAT[t], C8AcylCoAMAT[t], C4AcylCoAMAT[t], CarMAT],
  vcpt2C4 → CPT2[sfcpt2C4, Vcpt2, Kmcpt2C4AcylCarMAT, Kmcpt2C16AcylCarMAT,
    Kmcpt2C14AcylCarMAT, Kmcpt2C12AcylCarMAT, Kmcpt2C10AcylCarMAT,
    Kmcpt2C8AcylCarMAT, Kmcpt2C6AcylCarMAT, Kmcpt2CoAMAT,
    Kmcpt2C4AcylCoAMAT, Kmcpt2C16AcylCoAMAT, Kmcpt2C14AcylCoAMAT,
    Kmcpt2C12AcylCoAMAT, Kmcpt2C10AcylCoAMAT, Kmcpt2C8AcylCoAMAT,
    Kmcpt2C6AcylCoAMAT, Kmcpt2CarMAT, Keqcpt2, C4AcylCarMAT[t],
    C16AcylCarMAT[t], C14AcylCarMAT[t], C12AcylCarMAT[t],
    C10AcylCarMAT[t], C8AcylCarMAT[t], C6AcylCarMAT[t], CoAMAT,
    C4AcylCoAMAT[t], C16AcylCoAMAT[t], C14AcylCoAMAT[t], C12AcylCoAMAT[t],
    C10AcylCoAMAT[t], C8AcylCoAMAT[t], C6AcylCoAMAT[t], CarMAT],
  vvlcadC16 → VLCAD[sfvlcadC16, Vvlcad, KmvlcadC16AcylCoAMAT,
    KmvlcadC14AcylCoAMAT, KmvlcadC12AcylCoAMAT, KmvlcadFAD,
    KmvlcadC16EnoylCoAMAT, KmvlcadC14EnoylCoAMAT,
    KmvlcadC12EnoylCoAMAT, KmvlcadFADH, Keqvlcad, C16AcylCoAMAT[t],
    C14AcylCoAMAT[t], C12AcylCoAMAT[t], FADtMAT, C16EnoylCoAMAT[t],
    C14EnoylCoAMAT[t], C12EnoylCoAMAT[t], FADHMAT[t]],
  vvlcadC14 → VLCAD[sfvlcadC14, Vvlcad, KmvlcadC14AcylCoAMAT,
    KmvlcadC16AcylCoAMAT, KmvlcadC12AcylCoAMAT, KmvlcadFAD,
    KmvlcadC14EnoylCoAMAT, KmvlcadC16EnoylCoAMAT,
    KmvlcadC12EnoylCoAMAT, KmvlcadFADH, Keqvlcad, C14AcylCoAMAT[t],
    C16AcylCoAMAT[t], C12AcylCoAMAT[t], FADtMAT, C14EnoylCoAMAT[t],
    C16EnoylCoAMAT[t], C12EnoylCoAMAT[t], FADHMAT[t]],
  vvlcadC12 → VLCAD[sfvlcadC12, Vvlcad, KmvlcadC12AcylCoAMAT,
    KmvlcadC16AcylCoAMAT, KmvlcadC14AcylCoAMAT, KmvlcadFAD,
    KmvlcadC12EnoylCoAMAT, KmvlcadC16EnoylCoAMAT,
    KmvlcadC14EnoylCoAMAT, KmvlcadFADH, Keqvlcad, C12AcylCoAMAT[t],
    C16AcylCoAMAT[t], C14AcylCoAMAT[t], FADtMAT, C12EnoylCoAMAT[t],
    C16EnoylCoAMAT[t], C14EnoylCoAMAT[t], FADHMAT[t]],
  vlcadC16 → LCAD[sflcadC16, Vlcad, KmlcadC16AcylCoAMAT, KmlcadC14AcylCoAMAT,
    KmlcadC12AcylCoAMAT, KmlcadC10AcylCoAMAT, KmlcadC8AcylCoAMAT, KmlcadFAD,
    KmlcadC16EnoylCoAMAT, KmlcadC14EnoylCoAMAT, KmlcadC12EnoylCoAMAT,
    KmlcadC10EnoylCoAMAT, KmlcadC8EnoylCoAMAT, KmlcadFADH, Keqlcad,
    C16AcylCoAMAT[t], C14AcylCoAMAT[t], C12AcylCoAMAT[t], C10AcylCoAMAT[t],
    C8AcylCoAMAT[t], FADtMAT, C16EnoylCoAMAT[t], C14EnoylCoAMAT[t],
    C12EnoylCoAMAT[t], C10EnoylCoAMAT[t], C8EnoylCoAMAT[t], FADHMAT[t]],
  vlcadC14 → LCAD[sflcadC14, Vlcad, KmlcadC14AcylCoAMAT, KmlcadC16AcylCoAMAT,
    KmlcadC12AcylCoAMAT, KmlcadC10AcylCoAMAT, KmlcadC8AcylCoAMAT, KmlcadFAD,
    KmlcadC14EnoylCoAMAT, KmlcadC16EnoylCoAMAT, KmlcadC12EnoylCoAMAT,
    KmlcadC10EnoylCoAMAT, KmlcadC8EnoylCoAMAT, KmlcadFADH, Keqlcad,
    C14AcylCoAMAT[t], C16AcylCoAMAT[t], C12AcylCoAMAT[t], C10AcylCoAMAT[t],
    C8AcylCoAMAT[t], FADtMAT, C14EnoylCoAMAT[t], C16EnoylCoAMAT[t],
    C12EnoylCoAMAT[t], C10EnoylCoAMAT[t], C8EnoylCoAMAT[t], FADHMAT[t]],
  vlcadC12 → LCAD[sflcadC12, Vlcad, KmlcadC12AcylCoAMAT, KmlcadC16AcylCoAMAT,
    KmlcadC14AcylCoAMAT, KmlcadC10AcylCoAMAT, KmlcadC8AcylCoAMAT, KmlcadFAD,
    KmlcadC12EnoylCoAMAT, KmlcadC16EnoylCoAMAT, KmlcadC14EnoylCoAMAT,
    KmlcadC10EnoylCoAMAT, KmlcadC8EnoylCoAMAT, KmlcadFADH, Keqlcad,
    C12AcylCoAMAT[t], C16AcylCoAMAT[t], C14AcylCoAMAT[t], C10AcylCoAMAT[t],
    C8AcylCoAMAT[t], FADtMAT, C14EnoylCoAMAT[t], C16EnoylCoAMAT[t],
```

```
    C14EnoylCoAMAT[t], C10EnoylCoAMAT[t], C8EnoylCoAMAT[t], FADHMAT[t]],
  vlcadC10 → LCAD[sflcadC10, Vlcad, KmlcadC10AcylCoAMAT, KmlcadC16AcylCoAMAT,
    KmlcadC14AcylCoAMAT, KmlcadC12AcylCoAMAT, KmlcadC8AcylCoAMAT, KmlcadFAD,
    KmlcadC10EnoylCoAMAT, KmlcadC16EnoylCoAMAT, KmlcadC14EnoylCoAMAT,
    KmlcadC12EnoylCoAMAT, KmlcadC8EnoylCoAMAT, KmlcadFADH, Keqlcad,
    C10AcylCoAMAT[t], C16AcylCoAMAT[t], C14AcylCoAMAT[t], C12AcylCoAMAT[t],
    C8AcylCoAMAT[t], FADtMAT, C10EnoylCoAMAT[t], C16EnoylCoAMAT[t],
    C14EnoylCoAMAT[t], C12EnoylCoAMAT[t], C8EnoylCoAMAT[t], FADHMAT[t]],
  vlcadC8 → LCAD[sflcadC8, Vlcad, KmlcadC8AcylCoAMAT, KmlcadC16AcylCoAMAT,
    KmlcadC14AcylCoAMAT, KmlcadC12AcylCoAMAT, KmlcadC10AcylCoAMAT, KmlcadFAD,
    KmlcadC8EnoylCoAMAT, KmlcadC16EnoylCoAMAT, KmlcadC14EnoylCoAMAT,
    KmlcadC12EnoylCoAMAT, KmlcadC10EnoylCoAMAT, KmlcadFADH, Keqlcad,
    C8AcylCoAMAT[t], C16AcylCoAMAT[t], C14AcylCoAMAT[t], C12AcylCoAMAT[t],
    C10AcylCoAMAT[t], FADtMAT, C8EnoylCoAMAT[t], C16EnoylCoAMAT[t],
    C14EnoylCoAMAT[t], C12EnoylCoAMAT[t], C10EnoylCoAMAT[t], FADHMAT[t]],
  vmcadC12 → MCAD[sfmcadC12, Vmcad, KmmcadC12AcylCoAMAT, KmmcadC10AcylCoAMAT,
    KmmcadC8AcylCoAMAT, KmmcadC6AcylCoAMAT, KmmcadC4AcylCoAMAT, KmmcadFAD,
    KmmcadC12EnoylCoAMAT, KmmcadC10EnoylCoAMAT, KmmcadC8EnoylCoAMAT,
    KmmcadC6EnoylCoAMAT, KmmcadC4EnoylCoAMAT, KmmcadFADH, Keqmcad,
    C12AcylCoAMAT[t], C10AcylCoAMAT[t], C8AcylCoAMAT[t], C6AcylCoAMAT[t],
    C4AcylCoAMAT[t], FADtMAT, C12EnoylCoAMAT[t], C10EnoylCoAMAT[t],
    C8EnoylCoAMAT[t], C6EnoylCoAMAT[t], C4EnoylCoAMAT[t], FADHMAT[t]],
  vmcadC10 → MCAD[sfmcadC10, Vmcad, KmmcadC10AcylCoAMAT, KmmcadC12AcylCoAMAT,
    KmmcadC8AcylCoAMAT, KmmcadC6AcylCoAMAT, KmmcadC4AcylCoAMAT, KmmcadFAD,
    KmmcadC10EnoylCoAMAT, KmmcadC12EnoylCoAMAT, KmmcadC8EnoylCoAMAT,
    KmmcadC6EnoylCoAMAT, KmmcadC4EnoylCoAMAT, KmmcadFADH, Keqmcad,
    C10AcylCoAMAT[t], C12AcylCoAMAT[t], C8AcylCoAMAT[t], C6AcylCoAMAT[t],
    C4AcylCoAMAT[t], FADtMAT, C10EnoylCoAMAT[t], C12EnoylCoAMAT[t],
    C8EnoylCoAMAT[t], C6EnoylCoAMAT[t], C4EnoylCoAMAT[t], FADHMAT[t]],
  vmcadC8 → MCAD[sfmcadC8, Vmcad, KmmcadC8AcylCoAMAT, KmmcadC12AcylCoAMAT,
    KmmcadC10AcylCoAMAT, KmmcadC6AcylCoAMAT, KmmcadC4AcylCoAMAT, KmmcadFAD,
    KmmcadC8EnoylCoAMAT, KmmcadC12EnoylCoAMAT, KmmcadC10EnoylCoAMAT,
    KmmcadC6EnoylCoAMAT, KmmcadC4EnoylCoAMAT, KmmcadFADH, Keqmcad,
    C8AcylCoAMAT[t], C12AcylCoAMAT[t], C10AcylCoAMAT[t], C6AcylCoAMAT[t],
    C4AcylCoAMAT[t], FADtMAT, C8EnoylCoAMAT[t], C12EnoylCoAMAT[t],
    C10EnoylCoAMAT[t], C6EnoylCoAMAT[t], C4EnoylCoAMAT[t], FADHMAT[t]],
  vmcadC6 → MCAD[sfmcadC6, Vmcad, KmmcadC6AcylCoAMAT, KmmcadC12AcylCoAMAT,
    KmmcadC10AcylCoAMAT, KmmcadC8AcylCoAMAT, KmmcadC4AcylCoAMAT, KmmcadFAD,
    KmmcadC6EnoylCoAMAT, KmmcadC12EnoylCoAMAT, KmmcadC10EnoylCoAMAT,
    KmmcadC8EnoylCoAMAT, KmmcadC4EnoylCoAMAT, KmmcadFADH, Keqmcad,
    C6AcylCoAMAT[t], C12AcylCoAMAT[t], C10AcylCoAMAT[t], C8AcylCoAMAT[t],
    C4AcylCoAMAT[t], FADtMAT, C6EnoylCoAMAT[t], C12EnoylCoAMAT[t],
    C10EnoylCoAMAT[t], C8EnoylCoAMAT[t], C4EnoylCoAMAT[t], FADHMAT[t]],
  vmcadC4 → MCAD[sfmcadC4, Vmcad, KmmcadC4AcylCoAMAT, KmmcadC12AcylCoAMAT,
    KmmcadC10AcylCoAMAT, KmmcadC8AcylCoAMAT, KmmcadC6AcylCoAMAT, KmmcadFAD,
    KmmcadC4EnoylCoAMAT, KmmcadC12EnoylCoAMAT, KmmcadC10EnoylCoAMAT,
    KmmcadC8EnoylCoAMAT, KmmcadC6EnoylCoAMAT, KmmcadFADH, Keqmcad,
    C4AcylCoAMAT[t], C12AcylCoAMAT[t], C10AcylCoAMAT[t], C8AcylCoAMAT[t],
    C6AcylCoAMAT[t], FADtMAT, C4EnoylCoAMAT[t], C12EnoylCoAMAT[t],
    C10EnoylCoAMAT[t], C8EnoylCoAMAT[t], C6EnoylCoAMAT[t], FADHMAT[t]],
  vscadC6 → SCAD[sfscadC6, Vscad, KmscadC6AcylCoAMAT, KmscadC4AcylCoAMAT,
    KmscadFAD, KmscadC6EnoylCoAMAT, KmscadC4EnoylCoAMAT,
    KmscadFADH, Keqscad, C6AcylCoAMAT[t], C4AcylCoAMAT[t],
```

```
    FADtMAT, C6EnoylCoAMAT[t], C4EnoylCoAMAT[t], FADHMAT[t]],
  vscadC4 → SCAD[sfscadC4, Vscad, KmscadC4AcylCoAMAT, KmscadC6AcylCoAMAT,
    KmscadFAD, KmscadC4EnoylCoAMAT, KmscadC6EnoylCoAMAT,
    KmscadFADH, Keqscad, C4AcylCoAMAT[t], C6AcylCoAMAT[t],
    FADtMAT, C4EnoylCoAMAT[t], C6EnoylCoAMAT[t], FADHMAT[t]],
  vcrotC16 → CROT[sfcrotC16, Vcrot, KmcrotC16EnoylCoAMAT, KmcrotC14EnoylCoAMAT,
    KmcrotC12EnoylCoAMAT, KmcrotC10EnoylCoAMAT, KmcrotC8EnoylCoAMAT,
    KmcrotC6EnoylCoAMAT, KmcrotC4EnoylCoAMAT, KmcrotC16HydroxyacylCoAMAT,
    KmcrotC14HydroxyacylCoAMAT, KmcrotC12HydroxyacylCoAMAT,
    KmcrotC10HydroxyacylCoAMAT, KmcrotC8HydroxyacylCoAMAT,
    KmcrotC6HydroxyacylCoAMAT, KmcrotC4HydroxyacylCoAMAT,
    KicrotC4AcetoacylCoA, Keqcrot, C16EnoylCoAMAT[t], C14EnoylCoAMAT[t],
    C12EnoylCoAMAT[t], C10EnoylCoAMAT[t], C8EnoylCoAMAT[t], C6EnoylCoAMAT[t],
    C4EnoylCoAMAT[t], C16HydroxyacylCoAMAT[t], C14HydroxyacylCoAMAT[t],
    C12HydroxyacylCoAMAT[t], C10HydroxyacylCoAMAT[t], C8HydroxyacylCoAMAT[t],
    C6HydroxyacylCoAMAT[t], C4HydroxyacylCoAMAT[t], C4AcetoacylCoAMAT[t]],
  vcrotC14 → CROT[sfcrotC14, Vcrot, KmcrotC14EnoylCoAMAT, KmcrotC16EnoylCoAMAT,
    KmcrotC12EnoylCoAMAT, KmcrotC10EnoylCoAMAT, KmcrotC8EnoylCoAMAT,
    KmcrotC6EnoylCoAMAT, KmcrotC4EnoylCoAMAT, KmcrotC14HydroxyacylCoAMAT,
    KmcrotC16HydroxyacylCoAMAT, KmcrotC12HydroxyacylCoAMAT,
    KmcrotC10HydroxyacylCoAMAT, KmcrotC8HydroxyacylCoAMAT,
    KmcrotC6HydroxyacylCoAMAT, KmcrotC4HydroxyacylCoAMAT,
    KicrotC4AcetoacylCoA, Keqcrot, C14EnoylCoAMAT[t], C16EnoylCoAMAT[t],
    C12EnoylCoAMAT[t], C10EnoylCoAMAT[t], C8EnoylCoAMAT[t], C6EnoylCoAMAT[t],
    C4EnoylCoAMAT[t], C14HydroxyacylCoAMAT[t], C16HydroxyacylCoAMAT[t],
    C12HydroxyacylCoAMAT[t], C10HydroxyacylCoAMAT[t], C8HydroxyacylCoAMAT[t],
    C6HydroxyacylCoAMAT[t], C4HydroxyacylCoAMAT[t], C4AcetoacylCoAMAT[t]],
  vcrotC12 → CROT[sfcrotC12, Vcrot, KmcrotC12EnoylCoAMAT, KmcrotC16EnoylCoAMAT,
    KmcrotC14EnoylCoAMAT, KmcrotC10EnoylCoAMAT, KmcrotC8EnoylCoAMAT,
    KmcrotC6EnoylCoAMAT, KmcrotC4EnoylCoAMAT, KmcrotC12HydroxyacylCoAMAT,
    KmcrotC16HydroxyacylCoAMAT, KmcrotC14HydroxyacylCoAMAT,
    KmcrotC10HydroxyacylCoAMAT, KmcrotC8HydroxyacylCoAMAT,
    KmcrotC6HydroxyacylCoAMAT, KmcrotC4HydroxyacylCoAMAT,
    KicrotC4AcetoacylCoA, Keqcrot, C12EnoylCoAMAT[t], C16EnoylCoAMAT[t],
    C14EnoylCoAMAT[t], C10EnoylCoAMAT[t], C8EnoylCoAMAT[t], C6EnoylCoAMAT[t],
    C4EnoylCoAMAT[t], C12HydroxyacylCoAMAT[t], C16HydroxyacylCoAMAT[t],
    C14HydroxyacylCoAMAT[t], C10HydroxyacylCoAMAT[t], C8HydroxyacylCoAMAT[t],
    C6HydroxyacylCoAMAT[t], C4HydroxyacylCoAMAT[t], C4AcetoacylCoAMAT[t]],
  vcrotC10 → CROT[sfcrotC10, Vcrot, KmcrotC10EnoylCoAMAT, KmcrotC16EnoylCoAMAT,
    KmcrotC14EnoylCoAMAT, KmcrotC12EnoylCoAMAT, KmcrotC8EnoylCoAMAT,
    KmcrotC6EnoylCoAMAT, KmcrotC4EnoylCoAMAT, KmcrotC10HydroxyacylCoAMAT,
    KmcrotC16HydroxyacylCoAMAT, KmcrotC14HydroxyacylCoAMAT,
    KmcrotC12HydroxyacylCoAMAT, KmcrotC8HydroxyacylCoAMAT,
    KmcrotC6HydroxyacylCoAMAT, KmcrotC4HydroxyacylCoAMAT,
    KicrotC4AcetoacylCoA, Keqcrot, C10EnoylCoAMAT[t], C16EnoylCoAMAT[t],
    C14EnoylCoAMAT[t], C12EnoylCoAMAT[t], C8EnoylCoAMAT[t], C6EnoylCoAMAT[t],
    C4EnoylCoAMAT[t], C10HydroxyacylCoAMAT[t], C16HydroxyacylCoAMAT[t],
    C14HydroxyacylCoAMAT[t], C12HydroxyacylCoAMAT[t], C8HydroxyacylCoAMAT[t],
    C6HydroxyacylCoAMAT[t], C4HydroxyacylCoAMAT[t], C4AcetoacylCoAMAT[t]],
  vcrotC8 → CROT[sfcrotC8, Vcrot, KmcrotC8EnoylCoAMAT, KmcrotC16EnoylCoAMAT,
    KmcrotC14EnoylCoAMAT, KmcrotC12EnoylCoAMAT, KmcrotC10EnoylCoAMAT,
    KmcrotC6EnoylCoAMAT, KmcrotC4EnoylCoAMAT, KmcrotC8HydroxyacylCoAMAT,
    KmcrotC16HydroxyacylCoAMAT, KmcrotC14HydroxyacylCoAMAT,
```

```
    KmcrotC12HydroxyacylCoAMAT, KmcrotC10HydroxyacylCoAMAT,
    KmcrotC6HydroxyacylCoAMAT, KmcrotC4HydroxyacylCoAMAT,
    KicrotC4AcetoacylCoA, Keqcrot, C8EnoylCoAMAT[t], C16EnoylCoAMAT[t],
    C14EnoylCoAMAT[t], C12EnoylCoAMAT[t], C10EnoylCoAMAT[t], C6EnoylCoAMAT[t],
    C4EnoylCoAMAT[t], C8HydroxyacylCoAMAT[t], C16HydroxyacylCoAMAT[t],
    C14HydroxyacylCoAMAT[t], C12HydroxyacylCoAMAT[t], C10HydroxyacylCoAMAT[t],
    C6HydroxyacylCoAMAT[t], C4HydroxyacylCoAMAT[t], C4AcetoacylCoAMAT[t]],
  vcrotC6 → CROT[sfcrotC6, Vcrot, KmcrotC6EnoylCoAMAT, KmcrotC16EnoylCoAMAT,
    KmcrotC14EnoylCoAMAT, KmcrotC12EnoylCoAMAT, KmcrotC10EnoylCoAMAT,
    KmcrotC8EnoylCoAMAT, KmcrotC4EnoylCoAMAT, KmcrotC6HydroxyacylCoAMAT,
    KmcrotC16HydroxyacylCoAMAT, KmcrotC14HydroxyacylCoAMAT,
    KmcrotC12HydroxyacylCoAMAT, KmcrotC10HydroxyacylCoAMAT,
    KmcrotC8HydroxyacylCoAMAT, KmcrotC4HydroxyacylCoAMAT,
    KicrotC4AcetoacylCoA, Keqcrot, C6EnoylCoAMAT[t], C16EnoylCoAMAT[t],
    C14EnoylCoAMAT[t], C12EnoylCoAMAT[t], C10EnoylCoAMAT[t], C8EnoylCoAMAT[t],
    C4EnoylCoAMAT[t], C6HydroxyacylCoAMAT[t], C16HydroxyacylCoAMAT[t],
    C14HydroxyacylCoAMAT[t], C12HydroxyacylCoAMAT[t], C10HydroxyacylCoAMAT[t],
    C8HydroxyacylCoAMAT[t], C4HydroxyacylCoAMAT[t], C4AcetoacylCoAMAT[t]],
  vcrotC4 → CROT[sfcrotC4, Vcrot, KmcrotC4EnoylCoAMAT, KmcrotC16EnoylCoAMAT,
    KmcrotC14EnoylCoAMAT, KmcrotC12EnoylCoAMAT, KmcrotC10EnoylCoAMAT,
    KmcrotC8EnoylCoAMAT, KmcrotC6EnoylCoAMAT, KmcrotC4HydroxyacylCoAMAT,
    KmcrotC16HydroxyacylCoAMAT, KmcrotC14HydroxyacylCoAMAT,
    KmcrotC12HydroxyacylCoAMAT, KmcrotC10HydroxyacylCoAMAT,
    KmcrotC8HydroxyacylCoAMAT, KmcrotC6HydroxyacylCoAMAT,
    KicrotC4AcetoacylCoA, Keqcrot, C4EnoylCoAMAT[t], C16EnoylCoAMAT[t],
    C14EnoylCoAMAT[t], C12EnoylCoAMAT[t], C10EnoylCoAMAT[t], C8EnoylCoAMAT[t],
    C6EnoylCoAMAT[t], C4HydroxyacylCoAMAT[t], C16HydroxyacylCoAMAT[t],
    C14HydroxyacylCoAMAT[t], C12HydroxyacylCoAMAT[t], C10HydroxyacylCoAMAT[t],
    C8HydroxyacylCoAMAT[t], C6HydroxyacylCoAMAT[t], C4AcetoacylCoAMAT[t]],
  vmschadC16 → MSCHAD[sfmschadC16, Vmschad, KmmschadC16HydroxyacylCoAMAT,
    KmmschadC14HydroxyacylCoAMAT, KmmschadC12HydroxyacylCoAMAT,
    KmmschadC10HydroxyacylCoAMAT, KmmschadC8HydroxyacylCoAMAT,
    KmmschadC6HydroxyacylCoAMAT, KmmschadC4HydroxyacylCoAMAT,
    KmmschadNADMAT, KmmschadC16KetoacylCoAMAT, KmmschadC14KetoacylCoAMAT,
    KmmschadC12KetoacylCoAMAT, KmmschadC10KetoacylCoAMAT,
    KmmschadC8KetoacylCoAMAT, KmmschadC6KetoacylCoAMAT,
    KmmschadC4AcetoacylCoAMAT, KmmschadNADHMAT, Keqmschad,
    C16HydroxyacylCoAMAT[t], C14HydroxyacylCoAMAT[t],
    C12HydroxyacylCoAMAT[t], C10HydroxyacylCoAMAT[t],
    C8HydroxyacylCoAMAT[t], C6HydroxyacylCoAMAT[t], C4HydroxyacylCoAMAT[t],
    NADtMAT, C16KetoacylCoAMAT[t], C14KetoacylCoAMAT[t],
    C12KetoacylCoAMAT[t], C10KetoacylCoAMAT[t], C8KetoacylCoAMAT[t],
    C6KetoacylCoAMAT[t], C4AcetoacylCoAMAT[t], NADHMAT[t]],
  vmschadC14 → MSCHAD[sfmschadC14, Vmschad, KmmschadC14HydroxyacylCoAMAT,
    KmmschadC16HydroxyacylCoAMAT, KmmschadC12HydroxyacylCoAMAT,
    KmmschadC10HydroxyacylCoAMAT, KmmschadC8HydroxyacylCoAMAT,
    KmmschadC6HydroxyacylCoAMAT, KmmschadC4HydroxyacylCoAMAT,
    KmmschadNADMAT, KmmschadC14KetoacylCoAMAT, KmmschadC16KetoacylCoAMAT,
    KmmschadC12KetoacylCoAMAT, KmmschadC10KetoacylCoAMAT,
    KmmschadC8KetoacylCoAMAT, KmmschadC6KetoacylCoAMAT,
    KmmschadC4AcetoacylCoAMAT, KmmschadNADHMAT, Keqmschad,
    C14HydroxyacylCoAMAT[t], C16HydroxyacylCoAMAT[t],
    C12HydroxyacylCoAMAT[t], C10HydroxyacylCoAMAT[t],
```

```
    C8HydroxyacylCoAMAT[t], C6HydroxyacylCoAMAT[t], C4HydroxyacylCoAMAT[t],
    NADtMAT, C14KetoacylCoAMAT[t], C16KetoacylCoAMAT[t],
    C12KetoacylCoAMAT[t], C10KetoacylCoAMAT[t], C8KetoacylCoAMAT[t],
    C6KetoacylCoAMAT[t], C4AcetoacylCoAMAT[t], NADHMAT[t]],
vmschadC12 → MSCHAD[sfmschadC12, Vmschad, KmmschadC12HydroxyacylCoAMAT,
    KmmschadC16HydroxyacylCoAMAT, KmmschadC14HydroxyacylCoAMAT,
    KmmschadC10HydroxyacylCoAMAT, KmmschadC8HydroxyacylCoAMAT,
    KmmschadC6HydroxyacylCoAMAT, KmmschadC4HydroxyacylCoAMAT,
    KmmschadNADMAT, KmmschadC12KetoacylCoAMAT, KmmschadC16KetoacylCoAMAT,
    KmmschadC14KetoacylCoAMAT, KmmschadC10KetoacylCoAMAT,
    KmmschadC8KetoacylCoAMAT, KmmschadC6KetoacylCoAMAT,
    KmmschadC4AcetoacylCoAMAT, KmmschadNADHMAT, Keqmschad,
    C12HydroxyacylCoAMAT[t], C16HydroxyacylCoAMAT[t],
    C14HydroxyacylCoAMAT[t], C10HydroxyacylCoAMAT[t],
    C8HydroxyacylCoAMAT[t], C6HydroxyacylCoAMAT[t], C4HydroxyacylCoAMAT[t],
    NADtMAT, C12KetoacylCoAMAT[t], C16KetoacylCoAMAT[t],
    C14KetoacylCoAMAT[t], C10KetoacylCoAMAT[t], C8KetoacylCoAMAT[t],
    C6KetoacylCoAMAT[t], C4AcetoacylCoAMAT[t], NADHMAT[t]],
vmschadC10 → MSCHAD[sfmschadC10, Vmschad, KmmschadC10HydroxyacylCoAMAT,
    KmmschadC16HydroxyacylCoAMAT, KmmschadC14HydroxyacylCoAMAT,
    KmmschadC12HydroxyacylCoAMAT, KmmschadC8HydroxyacylCoAMAT,
    KmmschadC6HydroxyacylCoAMAT, KmmschadC4HydroxyacylCoAMAT,
    KmmschadNADMAT, KmmschadC10KetoacylCoAMAT, KmmschadC16KetoacylCoAMAT,
    KmmschadC14KetoacylCoAMAT, KmmschadC12KetoacylCoAMAT,
    KmmschadC8KetoacylCoAMAT, KmmschadC6KetoacylCoAMAT,
    KmmschadC4AcetoacylCoAMAT, KmmschadNADHMAT, Keqmschad,
    C10HydroxyacylCoAMAT[t], C16HydroxyacylCoAMAT[t],
    C14HydroxyacylCoAMAT[t], C12HydroxyacylCoAMAT[t],
    C8HydroxyacylCoAMAT[t], C6HydroxyacylCoAMAT[t], C4HydroxyacylCoAMAT[t],
    NADtMAT, C10KetoacylCoAMAT[t], C16KetoacylCoAMAT[t],
    C14KetoacylCoAMAT[t], C12KetoacylCoAMAT[t], C8KetoacylCoAMAT[t],
    C6KetoacylCoAMAT[t], C4AcetoacylCoAMAT[t], NADHMAT[t]],
vmschadC8 → MSCHAD[sfmschadC8, Vmschad, KmmschadC8HydroxyacylCoAMAT,
    KmmschadC16HydroxyacylCoAMAT, KmmschadC14HydroxyacylCoAMAT,
    KmmschadC12HydroxyacylCoAMAT, KmmschadC10HydroxyacylCoAMAT,
    KmmschadC6HydroxyacylCoAMAT, KmmschadC4HydroxyacylCoAMAT,
    KmmschadNADMAT, KmmschadC8KetoacylCoAMAT, KmmschadC16KetoacylCoAMAT,
    KmmschadC14KetoacylCoAMAT, KmmschadC12KetoacylCoAMAT,
    KmmschadC10KetoacylCoAMAT, KmmschadC6KetoacylCoAMAT,
    KmmschadC4AcetoacylCoAMAT, KmmschadNADHMAT, Keqmschad,
    C8HydroxyacylCoAMAT[t], C16HydroxyacylCoAMAT[t],
    C14HydroxyacylCoAMAT[t], C12HydroxyacylCoAMAT[t],
    C10HydroxyacylCoAMAT[t], C6HydroxyacylCoAMAT[t], C4HydroxyacylCoAMAT[t],
    NADtMAT, C8KetoacylCoAMAT[t], C16KetoacylCoAMAT[t],
    C14KetoacylCoAMAT[t], C12KetoacylCoAMAT[t], C10KetoacylCoAMAT[t],
    C6KetoacylCoAMAT[t], C4AcetoacylCoAMAT[t], NADHMAT[t]],
vmschadC6 → MSCHAD[sfmschadC6, Vmschad, KmmschadC6HydroxyacylCoAMAT,
    KmmschadC16HydroxyacylCoAMAT, KmmschadC14HydroxyacylCoAMAT,
    KmmschadC12HydroxyacylCoAMAT, KmmschadC10HydroxyacylCoAMAT,
    KmmschadC8HydroxyacylCoAMAT, KmmschadC4HydroxyacylCoAMAT,
    KmmschadNADMAT, KmmschadC6KetoacylCoAMAT, KmmschadC16KetoacylCoAMAT,
    KmmschadC14KetoacylCoAMAT, KmmschadC12KetoacylCoAMAT,
    KmmschadC10KetoacylCoAMAT, KmmschadC8KetoacylCoAMAT,
```

```
    KmmschadC4AcetoacylCoAMAT, KmmschadNADHMAT, Keqmschad,
    C6HydroxyacylCoAMAT[t], C16HydroxyacylCoAMAT[t],
    C14HydroxyacylCoAMAT[t], C12HydroxyacylCoAMAT[t],
    C10HydroxyacylCoAMAT[t], C8HydroxyacylCoAMAT[t], C4HydroxyacylCoAMAT[t],
    NADtMAT, C6KetoacylCoAMAT[t], C16KetoacylCoAMAT[t],
    C14KetoacylCoAMAT[t], C12KetoacylCoAMAT[t], C10KetoacylCoAMAT[t],
    C8KetoacylCoAMAT[t], C4AcetoacylCoAMAT[t], NADHMAT[t]],
  vmschadC4 → MSCHAD[sfmschadC4, Vmschad, KmmschadC4HydroxyacylCoAMAT,
    KmmschadC16HydroxyacylCoAMAT, KmmschadC14HydroxyacylCoAMAT,
    KmmschadC12HydroxyacylCoAMAT, KmmschadC10HydroxyacylCoAMAT,
    KmmschadC8HydroxyacylCoAMAT, KmmschadC6HydroxyacylCoAMAT,
    KmmschadNADMAT, KmmschadC4AcetoacylCoAMAT, KmmschadC16KetoacylCoAMAT,
    KmmschadC14KetoacylCoAMAT, KmmschadC12KetoacylCoAMAT,
    KmmschadC10KetoacylCoAMAT, KmmschadC8KetoacylCoAMAT,
    KmmschadC6KetoacylCoAMAT, KmmschadNADHMAT, Keqmschad,
    C4HydroxyacylCoAMAT[t], C16HydroxyacylCoAMAT[t],
    C14HydroxyacylCoAMAT[t], C12HydroxyacylCoAMAT[t],
    C10HydroxyacylCoAMAT[t], C8HydroxyacylCoAMAT[t], C6HydroxyacylCoAMAT[t],
    NADtMAT, C4AcetoacylCoAMAT[t], C16KetoacylCoAMAT[t],
    C14KetoacylCoAMAT[t], C12KetoacylCoAMAT[t], C10KetoacylCoAMAT[t],
    C8KetoacylCoAMAT[t], C6KetoacylCoAMAT[t], NADHMAT[t]],
  vmckatC16 → MCKATA[sfmckatC16, Vmckat, KmmckatC16KetoacylCoAMAT,
    KmmckatC14KetoacylCoAMAT, KmmckatC12KetoacylCoAMAT,
    KmmckatC10KetoacylCoAMAT, KmmckatC8KetoacylCoAMAT,
    KmmckatC6KetoacylCoAMAT, KmmckatC4AcetoacylCoAMAT, KmmckatCoAMAT,
    KmmckatC14AcylCoAMAT, KmmckatC16AcylCoAMAT, KmmckatC12AcylCoAMAT,
    KmmckatC10AcylCoAMAT, KmmckatC8AcylCoAMAT, KmmckatC6AcylCoAMAT,
    KmmckatC4AcylCoAMAT, KmmckatAcetylCoAMAT, Keqmckat, C16KetoacylCoAMAT[t],
    C14KetoacylCoAMAT[t], C12KetoacylCoAMAT[t], C10KetoacylCoAMAT[t],
    C8KetoacylCoAMAT[t], C6KetoacylCoAMAT[t], C4AcetoacylCoAMAT[t], CoAMAT,
    C14AcylCoAMAT[t], C16AcylCoAMAT[t], C12AcylCoAMAT[t], C10AcylCoAMAT[t],
    C8AcylCoAMAT[t], C6AcylCoAMAT[t], C4AcylCoAMAT[t], AcetylCoAMAT[t]],
  vmckatC14 → MCKATA[sfmckatC14, Vmckat, KmmckatC14KetoacylCoAMAT,
    KmmckatC16KetoacylCoAMAT, KmmckatC12KetoacylCoAMAT,
    KmmckatC10KetoacylCoAMAT, KmmckatC8KetoacylCoAMAT,
    KmmckatC6KetoacylCoAMAT, KmmckatC4AcetoacylCoAMAT, KmmckatCoAMAT,
    KmmckatC12AcylCoAMAT, KmmckatC16AcylCoAMAT, KmmckatC14AcylCoAMAT,
    KmmckatC10AcylCoAMAT, KmmckatC8AcylCoAMAT, KmmckatC6AcylCoAMAT,
    KmmckatC4AcylCoAMAT, KmmckatAcetylCoAMAT, Keqmckat, C14KetoacylCoAMAT[t],
    C16KetoacylCoAMAT[t], C12KetoacylCoAMAT[t], C10KetoacylCoAMAT[t],
    C8KetoacylCoAMAT[t], C6KetoacylCoAMAT[t], C4AcetoacylCoAMAT[t], CoAMAT,
    C12AcylCoAMAT[t], C16AcylCoAMAT[t], C14AcylCoAMAT[t], C10AcylCoAMAT[t],
    C8AcylCoAMAT[t], C6AcylCoAMAT[t], C4AcylCoAMAT[t], AcetylCoAMAT[t]],
  vmckatC12 → MCKATA[sfmckatC12, Vmckat, KmmckatC12KetoacylCoAMAT,
    KmmckatC16KetoacylCoAMAT, KmmckatC14KetoacylCoAMAT,
    KmmckatC10KetoacylCoAMAT, KmmckatC8KetoacylCoAMAT,
    KmmckatC6KetoacylCoAMAT, KmmckatC4AcetoacylCoAMAT, KmmckatCoAMAT,
    KmmckatC10AcylCoAMAT, KmmckatC16AcylCoAMAT, KmmckatC14AcylCoAMAT,
    KmmckatC12AcylCoAMAT, KmmckatC8AcylCoAMAT, KmmckatC6AcylCoAMAT,
    KmmckatC4AcylCoAMAT, KmmckatAcetylCoAMAT, Keqmckat, C12KetoacylCoAMAT[t],
    C16KetoacylCoAMAT[t], C14KetoacylCoAMAT[t], C10KetoacylCoAMAT[t],
    C8KetoacylCoAMAT[t], C6KetoacylCoAMAT[t], C4AcetoacylCoAMAT[t], CoAMAT,
    C10AcylCoAMAT[t], C16AcylCoAMAT[t], C14AcylCoAMAT[t], C12AcylCoAMAT[t],
```

```
  C8AcylCoAMAT[t], C6AcylCoAMAT[t], C4AcylCoAMAT[t], AcetylCoAMAT[t]],
vmckatC10 → MCKATA[sfmckatC10, Vmckat, KmmckatC10KetoacylCoAMAT,
  KmmckatC16KetoacylCoAMAT, KmmckatC14KetoacylCoAMAT,
  KmmckatC12KetoacylCoAMAT, KmmckatC8KetoacylCoAMAT,
  KmmckatC6KetoacylCoAMAT, KmmckatC4AcetoacylCoAMAT, KmmckatCoAMAT,
  KmmckatC8AcylCoAMAT, KmmckatC16AcylCoAMAT, KmmckatC14AcylCoAMAT,
  KmmckatC12AcylCoAMAT, KmmckatC10AcylCoAMAT, KmmckatC6AcylCoAMAT,
  KmmckatC4AcylCoAMAT, KmmckatAcetylCoAMAT, Keqmckat, C10KetoacylCoAMAT[t],
  C16KetoacylCoAMAT[t], C14KetoacylCoAMAT[t], C12KetoacylCoAMAT[t],
  C8KetoacylCoAMAT[t], C6KetoacylCoAMAT[t], C4AcetoacylCoAMAT[t], CoAMAT,
  C8AcylCoAMAT[t], C16AcylCoAMAT[t], C14AcylCoAMAT[t], C12AcylCoAMAT[t],
  C10AcylCoAMAT[t], C6AcylCoAMAT[t], C4AcylCoAMAT[t], AcetylCoAMAT[t]],
vmckatC8 → MCKATA[sfmckatC8, Vmckat, KmmckatC8KetoacylCoAMAT,
  KmmckatC16KetoacylCoAMAT, KmmckatC14KetoacylCoAMAT,
  KmmckatC12KetoacylCoAMAT, KmmckatC10KetoacylCoAMAT,
  KmmckatC6KetoacylCoAMAT, KmmckatC4AcetoacylCoAMAT, KmmckatCoAMAT,
  KmmckatC6AcylCoAMAT, KmmckatC16AcylCoAMAT, KmmckatC14AcylCoAMAT,
  KmmckatC12AcylCoAMAT, KmmckatC10AcylCoAMAT, KmmckatC8AcylCoAMAT,
  KmmckatC4AcylCoAMAT, KmmckatAcetylCoAMAT, Keqmckat, C8KetoacylCoAMAT[t],
  C16KetoacylCoAMAT[t], C14KetoacylCoAMAT[t], C12KetoacylCoAMAT[t],
  C10KetoacylCoAMAT[t], C6KetoacylCoAMAT[t], C4AcetoacylCoAMAT[t], CoAMAT,
  C6AcylCoAMAT[t], C16AcylCoAMAT[t], C14AcylCoAMAT[t], C12AcylCoAMAT[t],
  C10AcylCoAMAT[t], C8AcylCoAMAT[t], C4AcylCoAMAT[t], AcetylCoAMAT[t]],
vmckatC6 → MCKATA[sfmckatC6, Vmckat, KmmckatC6KetoacylCoAMAT,
  KmmckatC16KetoacylCoAMAT, KmmckatC14KetoacylCoAMAT,
  KmmckatC12KetoacylCoAMAT, KmmckatC10KetoacylCoAMAT,
  KmmckatC8KetoacylCoAMAT, KmmckatC4AcetoacylCoAMAT, KmmckatCoAMAT,
  KmmckatC4AcylCoAMAT, KmmckatC16AcylCoAMAT, KmmckatC14AcylCoAMAT,
  KmmckatC12AcylCoAMAT, KmmckatC10AcylCoAMAT, KmmckatC8AcylCoAMAT,
  KmmckatC6AcylCoAMAT, KmmckatAcetylCoAMAT, Keqmckat, C6KetoacylCoAMAT[t],
  C16KetoacylCoAMAT[t], C14KetoacylCoAMAT[t], C12KetoacylCoAMAT[t],
  C10KetoacylCoAMAT[t], C8KetoacylCoAMAT[t], C4AcetoacylCoAMAT[t], CoAMAT,
  C4AcylCoAMAT[t], C16AcylCoAMAT[t], C14AcylCoAMAT[t], C12AcylCoAMAT[t],
  C10AcylCoAMAT[t], C8AcylCoAMAT[t], C6AcylCoAMAT[t], AcetylCoAMAT[t]],
vmckatC4 → MCKATB[sfmckatC4, Vmckat, KmmckatC4AcetoacylCoAMAT,
  KmmckatC16KetoacylCoAMAT, KmmckatC14KetoacylCoAMAT,
  KmmckatC12KetoacylCoAMAT, KmmckatC10KetoacylCoAMAT,
  KmmckatC8KetoacylCoAMAT, KmmckatC6KetoacylCoAMAT, KmmckatCoAMAT,
  KmmckatC4AcylCoAMAT, KmmckatC16AcylCoAMAT, KmmckatC14AcylCoAMAT,
  KmmckatC12AcylCoAMAT, KmmckatC10AcylCoAMAT, KmmckatC8AcylCoAMAT,
  KmmckatC6AcylCoAMAT, KmmckatAcetylCoAMAT, Keqmckat, C4AcetoacylCoAMAT[t],
  C16KetoacylCoAMAT[t], C14KetoacylCoAMAT[t], C12KetoacylCoAMAT[t],
  C10KetoacylCoAMAT[t], C8KetoacylCoAMAT[t], C6KetoacylCoAMAT[t], CoAMAT,
  C4AcylCoAMAT[t], C16AcylCoAMAT[t], C14AcylCoAMAT[t], C12AcylCoAMAT[t],
  C10AcylCoAMAT[t], C8AcylCoAMAT[t], C6AcylCoAMAT[t], AcetylCoAMAT[t]],
vmtpC16 → MTP[sfmtpC16, Vmtp, KmmtpC16EnoylCoAMAT, KmmtpC14EnoylCoAMAT,
  KmmtpC12EnoylCoAMAT, KmmtpC10EnoylCoAMAT, KmmtpC8EnoylCoAMAT,
  KmmtpNADMAT, KmmtpCoAMAT, KmmtpC14AcylCoAMAT, KmmtpC16AcylCoAMAT,
  KmmtpC12AcylCoAMAT, KmmtpC10AcylCoAMAT, KmmtpC8AcylCoAMAT,
  KmmtpC6AcylCoAMAT, KmmtpNADHMAT, KmmtpAcetylCoAMAT, KicrotC4AcetoacylCoA,
  Keqmtp, C16EnoylCoAMAT[t], C14EnoylCoAMAT[t], C12EnoylCoAMAT[t],
  C10EnoylCoAMAT[t], C8EnoylCoAMAT[t], NADtMAT, CoAMAT, C14AcylCoAMAT[t],
  C16AcylCoAMAT[t], C12AcylCoAMAT[t], C10AcylCoAMAT[t], C8AcylCoAMAT[t],
```

```
    C6AcylCoAMAT[t], NADHMAT[t], AcetylCoAMAT[t], C4AcetoacylCoAMAT[t]],
  vmtpC14 → MTP[sfmtpC14, Vmtp, KmmtpC14EnoylCoAMAT, KmmtpC16EnoylCoAMAT,
    KmmtpC12EnoylCoAMAT, KmmtpC10EnoylCoAMAT, KmmtpC8EnoylCoAMAT,
    KmmtpNADMAT, KmmtpCoAMAT, KmmtpC12AcylCoAMAT, KmmtpC16AcylCoAMAT,
    KmmtpC14AcylCoAMAT, KmmtpC10AcylCoAMAT, KmmtpC8AcylCoAMAT,
    KmmtpC6AcylCoAMAT, KmmtpNADHMAT, KmmtpAcetylCoAMAT, KicrotC4AcetoacylCoA,
    Keqmtp, C14EnoylCoAMAT[t], C16EnoylCoAMAT[t], C12EnoylCoAMAT[t],
    C10EnoylCoAMAT[t], C8EnoylCoAMAT[t], NADtMAT, CoAMAT, C12AcylCoAMAT[t],
    C16AcylCoAMAT[t], C14AcylCoAMAT[t], C10AcylCoAMAT[t], C8AcylCoAMAT[t],
    C6AcylCoAMAT[t], NADHMAT[t], AcetylCoAMAT[t], C4AcetoacylCoAMAT[t]],
  vmtpC12 → MTP[sfmtpC12, Vmtp, KmmtpC12EnoylCoAMAT, KmmtpC16EnoylCoAMAT,
    KmmtpC14EnoylCoAMAT, KmmtpC10EnoylCoAMAT, KmmtpC8EnoylCoAMAT,
    KmmtpNADMAT, KmmtpCoAMAT, KmmtpC10AcylCoAMAT, KmmtpC16AcylCoAMAT,
    KmmtpC14AcylCoAMAT, KmmtpC12AcylCoAMAT, KmmtpC8AcylCoAMAT,
    KmmtpC6AcylCoAMAT, KmmtpNADHMAT, KmmtpAcetylCoAMAT, KicrotC4AcetoacylCoA,
    Keqmtp, C12EnoylCoAMAT[t], C16EnoylCoAMAT[t], C14EnoylCoAMAT[t],
    C10EnoylCoAMAT[t], C8EnoylCoAMAT[t], NADtMAT, CoAMAT, C10AcylCoAMAT[t],
    C16AcylCoAMAT[t], C14AcylCoAMAT[t], C12AcylCoAMAT[t], C8AcylCoAMAT[t],
    C6AcylCoAMAT[t], NADHMAT[t], AcetylCoAMAT[t], C4AcetoacylCoAMAT[t]],
  vmtpC10 → MTP[sfmtpC10, Vmtp, KmmtpC10EnoylCoAMAT, KmmtpC16EnoylCoAMAT,
    KmmtpC14EnoylCoAMAT, KmmtpC12EnoylCoAMAT, KmmtpC8EnoylCoAMAT,
    KmmtpNADMAT, KmmtpCoAMAT, KmmtpC8AcylCoAMAT, KmmtpC16AcylCoAMAT,
    KmmtpC14AcylCoAMAT, KmmtpC12AcylCoAMAT, KmmtpC10AcylCoAMAT,
    KmmtpC6AcylCoAMAT, KmmtpNADHMAT, KmmtpAcetylCoAMAT, KicrotC4AcetoacylCoA,
    Keqmtp, C10EnoylCoAMAT[t], C16EnoylCoAMAT[t], C14EnoylCoAMAT[t],
    C12EnoylCoAMAT[t], C8EnoylCoAMAT[t], NADtMAT, CoAMAT, C8AcylCoAMAT[t],
    C16AcylCoAMAT[t], C14AcylCoAMAT[t], C12AcylCoAMAT[t], C10AcylCoAMAT[t],
    C6AcylCoAMAT[t], NADHMAT[t], AcetylCoAMAT[t], C4AcetoacylCoAMAT[t]],
  vmtpC8 → MTP[sfmtpC8, Vmtp, KmmtpC8EnoylCoAMAT, KmmtpC16EnoylCoAMAT,
    KmmtpC14EnoylCoAMAT, KmmtpC12EnoylCoAMAT, KmmtpC10EnoylCoAMAT,
    KmmtpNADMAT, KmmtpCoAMAT, KmmtpC6AcylCoAMAT, KmmtpC16AcylCoAMAT,
    KmmtpC14AcylCoAMAT, KmmtpC12AcylCoAMAT, KmmtpC10AcylCoAMAT,
    KmmtpC8AcylCoAMAT, KmmtpNADHMAT, KmmtpAcetylCoAMAT, KicrotC4AcetoacylCoA,
    Keqmtp, C8EnoylCoAMAT[t], C16EnoylCoAMAT[t], C14EnoylCoAMAT[t],
    C12EnoylCoAMAT[t], C10EnoylCoAMAT[t], NADtMAT, CoAMAT, C6AcylCoAMAT[t],
    C16AcylCoAMAT[t], C14AcylCoAMAT[t], C12AcylCoAMAT[t], C10AcylCoAMAT[t],
    C8AcylCoAMAT[t], NADHMAT[t], AcetylCoAMAT[t], C4AcetoacylCoAMAT[t]],
  vacesink → RES[Ksacesink, AcetylCoAMAT[t], K1acesink],
  vfadhsink → RES[Ksfadhsink, FADHMAT[t], K1fadhsink],
  vnadhsink → RES[Ksnadhsink, NADHMAT[t], K1nadhsink]};

CoAMATX = {CoAMAT → CoAMATt - C16AcylCoAMAT[t] - C16EnoylCoAMAT[t] -
    C16HydroxyacylCoAMAT[t] - C16KetoacylCoAMAT[t] - C14AcylCoAMAT[t] -
    C14EnoylCoAMAT[t] - C14HydroxyacylCoAMAT[t] - C14KetoacylCoAMAT[t] -
    C12AcylCoAMAT[t] - C12EnoylCoAMAT[t] - C12HydroxyacylCoAMAT[t] -
    C12KetoacylCoAMAT[t] - C10AcylCoAMAT[t] - C10EnoylCoAMAT[t] -
    C10HydroxyacylCoAMAT[t] - C10KetoacylCoAMAT[t] - C8AcylCoAMAT[t] -
    C8EnoylCoAMAT[t] - C8HydroxyacylCoAMAT[t] - C8KetoacylCoAMAT[t] -
    C6AcylCoAMAT[t] - C6EnoylCoAMAT[t] - C6HydroxyacylCoAMAT[t] -
    C6KetoacylCoAMAT[t] - C4AcylCoAMAT[t] - C4EnoylCoAMAT[t] -
    C4HydroxyacylCoAMAT[t] - C4AcetoacylCoAMAT[t] - AcetylCoAMAT[t]};

Parm = {
```

```
sfcpt1C16 → 1, Vcpt1 → 0.012, Kmcpt1C16AcylCoACYT → 13.8,
Kmcpt1CarCYT → 250, Kmcpt1C16AcylCarCYT → 136, Kmcpt1CoACYT → 40.7,
Kicpt1MalCoACYT → 9.1, Keqcpt1 → 0.45, ncpt1 → 2.4799,
Vfcact → 0.42, Vrcact → 0.42, KmcactC16AcylCarCYT → 15,
KmcactC14AcylCarCYT → 15, KmcactC12AcylCarCYT → 15, KmcactC10AcylCarCYT → 15,
KmcactC8AcylCarCYT → 15, KmcactC6AcylCarCYT → 15, KmcactC4AcylCarCYT → 15,
KmcactCarMAT → 130, KmcactC16AcylCarMAT → 15, KmcactC14AcylCarMAT → 15,
KmcactC12AcylCarMAT → 15, KmcactC10AcylCarMAT → 15, KmcactC8AcylCarMAT → 15,
KmcactC6AcylCarMAT → 15, KmcactC4AcylCarMAT → 15, KmcactCarCYT → 130,
KicactC16AcylCarCYT → 56, KicactC14AcylCarCYT → 56, KicactC12AcylCarCYT → 56,
KicactC10AcylCarCYT → 56, KicactC8AcylCarCYT → 56, KicactC6AcylCarCYT → 56,
KicactC4AcylCarCYT → 56, KicactCarCYT → 200, Keqcact → 1,
sfcpt2C16 → 0.85, sfcpt2C14 → 1, sfcpt2C12 → 0.95, sfcpt2C10 → 0.95,
sfcpt2C8 → 0.35, sfcpt2C6 → 0.15, sfcpt2C4 → 0.01, Vcpt2 → 0.391,
Kmcpt2C16AcylCarMAT → 51, Kmcpt2C14AcylCarMAT → 51, Kmcpt2C12AcylCarMAT → 51,
Kmcpt2C10AcylCarMAT → 51, Kmcpt2C8AcylCarMAT → 51, Kmcpt2C6AcylCarMAT → 51,
Kmcpt2C4AcylCarMAT → 51, Kmcpt2CoAMAT → 30, Kmcpt2C16AcylCoAMAT → 38,
Kmcpt2C14AcylCoAMAT → 38, Kmcpt2C12AcylCoAMAT → 38,
Kmcpt2C10AcylCoAMAT → 38, Kmcpt2C8AcylCoAMAT → 38, Kmcpt2C6AcylCoAMAT → 1000,
Kmcpt2C4AcylCoAMAT → 1 000 000, Kmcpt2CarMAT → 350, Keqcpt2 → 2.22,
sfvlcadC16 → 1, sfvlcadC14 → 0.42, sfvlcadC12 → 0.11, Vvlcad → 0.008,
KmvlcadC16AcylCoAMAT → 6.5, KmvlcadC14AcylCoAMAT → 4,
KmvlcadC12AcylCoAMAT → 2.7, KmvlcadFAD → 0.12,
KmvlcadC16EnoylCoAMAT → 1.08, KmvlcadC14EnoylCoAMAT → 1.08,
KmvlcadC12EnoylCoAMAT → 1.08, KmvlcadFADH → 24.2, Keqvlcad → 6,
sflcadC16 → 0.9, sflcadC14 → 1, sflcadC12 → 0.9, sflcadC10 → 0.75,
sflcadC8 → 0.4, Vlcad → 0.01, KmlcadC16AcylCoAMAT → 2.5,
KmlcadC14AcylCoAMAT → 7.4, KmlcadC12AcylCoAMAT → 9,
KmlcadC10AcylCoAMAT → 24.3, KmlcadC8AcylCoAMAT → 123, KmlcadFAD → 0.12,
KmlcadC16EnoylCoAMAT → 1.08, KmlcadC14EnoylCoAMAT → 1.08,
KmlcadC12EnoylCoAMAT → 1.08, KmlcadC10EnoylCoAMAT → 1.08,
KmlcadC8EnoylCoAMAT → 1.08, KmlcadFADH → 24.2, Keqlcad → 6,
sfmcadC12 → 0.38, sfmcadC10 → 0.8, sfmcadC8 → 0.87, sfmcadC6 → 1,
sfmcadC4 → 0.12, Vmcad → 0.081, KmmcadC12AcylCoAMAT → 5.7,
KmmcadC10AcylCoAMAT → 5.4, KmmcadC8AcylCoAMAT → 4,
KmmcadC6AcylCoAMAT → 9.4, KmmcadC4AcylCoAMAT → 135, KmmcadFAD → 0.12,
KmmcadC12EnoylCoAMAT → 1.08, KmmcadC10EnoylCoAMAT → 1.08,
KmmcadC8EnoylCoAMAT → 1.08, KmmcadC6EnoylCoAMAT → 1.08,
KmmcadC4EnoylCoAMAT → 1.08, KmmcadFADH → 24.2, Keqmcad → 6,
sfscadC6 → 0.3, sfscadC4 → 1, Vscad → 0.081, KmscadC6AcylCoAMAT → 285,
KmscadC4AcylCoAMAT → 10.7, KmscadFAD → 0.12, KmscadC6EnoylCoAMAT → 1.08,
KmscadC4EnoylCoAMAT → 1.08, KmscadFADH → 24.2, Keqscad -> 6,
sfcrotC16 → 0.13, sfcrotC14 → 0.2, sfcrotC12 → 0.25, sfcrotC10 → 0.33,
sfcrotC8 → 0.58, sfcrotC6 → 0.83, sfcrotC4 → 1, Vcrot → 3.6,
KmcrotC16EnoylCoAMAT → 150, KmcrotC14EnoylCoAMAT → 100,
KmcrotC12EnoylCoAMAT → 25, KmcrotC10EnoylCoAMAT → 25,
KmcrotC8EnoylCoAMAT → 25, KmcrotC6EnoylCoAMAT → 25, KmcrotC4EnoylCoAMAT → 40,
KmcrotC16HydroxyacylCoAMAT → 45, KmcrotC14HydroxyacylCoAMAT → 45,
KmcrotC12HydroxyacylCoAMAT → 45, KmcrotC10HydroxyacylCoAMAT → 45,
KmcrotC8HydroxyacylCoAMAT → 45, KmcrotC6HydroxyacylCoAMAT → 45,
KmcrotC4HydroxyacylCoAMAT → 45, KicrotC4AcetoacylCoA → 1.6, Keqcrot → 3.13,
sfmschadC16 → 0.6, sfmschadC14 → 0.5, sfmschadC12 → 0.43,
sfmschadC10 → 0.64, sfmschadC8 → 0.89, sfmschadC6 → 1,
```

```
sfmschadC4 → 0.67, Vmschad → 1, KmmschadC16HydroxyacylCoAMAT → 1.5,
KmmschadC14HydroxyacylCoAMAT → 1.8, KmmschadC12HydroxyacylCoAMAT → 3.7,
KmmschadC10HydroxyacylCoAMAT → 8.8, KmmschadC8HydroxyacylCoAMAT → 16.3,
KmmschadC6HydroxyacylCoAMAT → 28.6, KmmschadC4HydroxyacylCoAMAT → 69.9,
KmmschadNADMAT → 58.5, KmmschadC16KetoacylCoAMAT → 1.4,
KmmschadC14KetoacylCoAMAT → 1.4, KmmschadC12KetoacylCoAMAT → 1.6,
KmmschadC10KetoacylCoAMAT → 2.3, KmmschadC8KetoacylCoAMAT → 4.1,
KmmschadC6KetoacylCoAMAT → 5.8, KmmschadC4AcetoacylCoAMAT → 16.9,
KmmschadNADHMAT → 5.4, Keqmschad → 2.17 * 10^-4,
sfmckatC16 → 0, sfmckatC14 → 0.2, sfmckatC12 → 0.38, sfmckatC10 → 0.65,
sfmckatC8 → 0.81, sfmckatC6 → 1, sfmckatC4 → 0.49, Vmckat → 0.377,
KmmckatC16KetoacylCoAMAT → 1.1, KmmckatC14KetoacylCoAMAT → 1.2,
KmmckatC12KetoacylCoAMAT → 1.3, KmmckatC10KetoacylCoAMAT → 2.1,
KmmckatC8KetoacylCoAMAT → 3.2, KmmckatC6KetoacylCoAMAT → 6.7,
KmmckatC4AcetoacylCoAMAT → 12.4, KmmckatCoAMAT → 26.6,
KmmckatC14AcylCoAMAT → 13.83, KmmckatC16AcylCoAMAT → 13.83,
KmmckatC12AcylCoAMAT → 13.83, KmmckatC10AcylCoAMAT → 13.83,
KmmckatC8AcylCoAMAT → 13.83, KmmckatC6AcylCoAMAT → 13.83,
KmmckatC4AcylCoAMAT → 13.83, KmmckatAcetylCoAMAT → 30, Keqmckat → 1051,
sfmtpC16 → 1, sfmtpC14 → 0.9, sfmtpC12 → 0.81, sfmtpC10 → 0.73,
sfmtpC8 → 0.34, Vmtp → 2.84, KmmtpC16EnoylCoAMAT → 25,
KmmtpC14EnoylCoAMAT → 25, KmmtpC12EnoylCoAMAT → 25,
KmmtpC10EnoylCoAMAT → 25, KmmtpC8EnoylCoAMAT → 25, KmmtpNADMAT → 60,
KmmtpCoAMAT → 30, KmmtpC14AcylCoAMAT → 13.83, KmmtpC16AcylCoAMAT → 13.83,
KmmtpC12AcylCoAMAT → 13.83, KmmtpC10AcylCoAMAT → 13.83,
KmmtpC8AcylCoAMAT → 13.83, KmmtpC6AcylCoAMAT → 13.83,
KmmtpNADHMAT → 50, KmmtpAcetylCoAMAT → 30, Keqmtp → 0.71,
Ksacesink → 6 000 000, K1acesink → 70, Ksfadhsink → 6 000 000,
K1fadhsink → 0.46, Ksnadhsink → 6 000 000, K1nadhsink → 16,
C16AcylCoACYT → 25, CarCYT → 200, CoACYT → 140, MalCoACYT → 0,
CarMAT -> 950, FADtMAT → 0.77, NADtMAT → 250, CoAMATt → 5000,
VCYT → 2.2 * 10^-6, VMAT → 1.8 * 10^-6};

InitialConditions = {
  C16AcylCarCYT[0] == 0, C16AcylCarMAT[0] == 0, C16AcylCoAMAT[0] == 0,
  C16EnoylCoAMAT[0] == 0, C16HydroxyacylCoAMAT[0] == 0, C16KetoacylCoAMAT[0] == 0,
  C14AcylCarCYT[0] == 0, C14AcylCarMAT[0] == 0, C14AcylCoAMAT[0] == 0,
  C14EnoylCoAMAT[0] == 0, C14HydroxyacylCoAMAT[0] == 0, C14KetoacylCoAMAT[0] == 0,
  C12AcylCarCYT[0] == 0, C12AcylCarMAT[0] == 0, C12AcylCoAMAT[0] == 0,
  C12EnoylCoAMAT[0] == 0, C12HydroxyacylCoAMAT[0] == 0, C12KetoacylCoAMAT[0] == 0,
  C10AcylCarCYT[0] == 0, C10AcylCarMAT[0] == 0, C10AcylCoAMAT[0] == 0,
  C10EnoylCoAMAT[0] == 0, C10HydroxyacylCoAMAT[0] == 0, C10KetoacylCoAMAT[0] == 0,
  C8AcylCarCYT[0] == 0, C8AcylCarMAT[0] == 0, C8AcylCoAMAT[0] == 0,
  C8EnoylCoAMAT[0] == 0, C8HydroxyacylCoAMAT[0] == 0, C8KetoacylCoAMAT[0] == 0,
  C6AcylCarCYT[0] == 0, C6AcylCarMAT[0] == 0, C6AcylCoAMAT[0] == 0,
  C6EnoylCoAMAT[0] == 0, C6HydroxyacylCoAMAT[0] == 0, C6KetoacylCoAMAT[0] == 0,
  C4AcylCarCYT[0] == 0, C4AcylCarMAT[0] == 0, C4AcylCoAMAT[0] == 0,
  C4EnoylCoAMAT[0] == 0, C4HydroxyacylCoAMAT[0] == 0, C4AcetoacylCoAMAT[0] == 0,
  AcetylCoAMAT[0] == 70, FADHMAT[0] == 0.46, NADHMAT[0] == 16};

Vars = {
  C16AcylCarCYT, C16AcylCarMAT, C16AcylCoAMAT,
  C16EnoylCoAMAT, C16HydroxyacylCoAMAT, C16KetoacylCoAMAT,
```

```
    C14AcylCarCYT, C14AcylCarMAT, C14AcylCoAMAT, C14EnoylCoAMAT,
    C14HydroxyacylCoAMAT, C14KetoacylCoAMAT,
    C12AcylCarCYT, C12AcylCarMAT, C12AcylCoAMAT, C12EnoylCoAMAT,
    C12HydroxyacylCoAMAT, C12KetoacylCoAMAT,
    C10AcylCarCYT, C10AcylCarMAT, C10AcylCoAMAT, C10EnoylCoAMAT,
    C10HydroxyacylCoAMAT, C10KetoacylCoAMAT,
    C8AcylCarCYT, C8AcylCarMAT, C8AcylCoAMAT, C8EnoylCoAMAT,
    C8HydroxyacylCoAMAT, C8KetoacylCoAMAT,
    C6AcylCarCYT, C6AcylCarMAT, C6AcylCoAMAT, C6EnoylCoAMAT,
    C6HydroxyacylCoAMAT, C6KetoacylCoAMAT,
    C4AcylCarCYT, C4AcylCarMAT, C4AcylCoAMAT, C4EnoylCoAMAT,
    C4HydroxyacylCoAMAT, C4AcetoacylCoAMAT,
    AcetylCoAMAT, FADHMAT, NADHMAT};

TableForm[Odes];
TableForm[RateEqs];
TableForm[Odes /. RateEqs /. CoAMATX /. Parm];
TableForm[RateEqs /. Parm];
TableForm[InitialConditions];

tsol = NDSolve[Join[Odes /. RateEqs /. CoAMATX /. Parm, InitialConditions],
    Vars, {t, 0, 1000000000}];
```

Steady-state values

```
Table[{Vars[[i]][t], (Vars[[i]][900000000] /. tsol)[[1]]}, {i, 1, Length[Vars]}];

ss1 = FindRoot[
   Table[Odes[[i, 2]] == 0, {i, 1, Length[Odes]}] /. RateEqs /. CoAMATX /. Parm, %]

fluxes1 = RateEqs /. CoAMATX /. Parm /. ss1

Metabolites =
  TableForm[Table[{ss1[[i]][[1]], ss1[[i]][[2]]}, {i, 1, Length[ss1]}]]

Flux = TableForm[
   Table[{fluxes1[[i]][[1]], fluxes1[[i]][[2]]}, {i, 1, Length[fluxes1]}]]

CoAMAT1 = CoAMATt - C16AcylCoAMAT[t] - C16EnoylCoAMAT[t] - C16HydroxyacylCoAMAT[t] -
     C16KetoacylCoAMAT[t] - C14AcylCoAMAT[t] - C14EnoylCoAMAT[t] -
     C14HydroxyacylCoAMAT[t] - C14KetoacylCoAMAT[t] - C12AcylCoAMAT[t] -
     C12EnoylCoAMAT[t] - C12HydroxyacylCoAMAT[t] - C12KetoacylCoAMAT[t] -
     C10AcylCoAMAT[t] - C10EnoylCoAMAT[t] - C10HydroxyacylCoAMAT[t] -
     C10KetoacylCoAMAT[t] - C8AcylCoAMAT[t] - C8EnoylCoAMAT[t] -
     C8HydroxyacylCoAMAT[t] - C8KetoacylCoAMAT[t] - C6AcylCoAMAT[t] -
     C6EnoylCoAMAT[t] - C6HydroxyacylCoAMAT[t] - C6KetoacylCoAMAT[t] -
     C4AcylCoAMAT[t] - C4EnoylCoAMAT[t] - C4HydroxyacylCoAMAT[t] -
     C4AcetoacylCoAMAT[t] - AcetylCoAMAT[t] /. RateEqs /. Parm /. ss1
```
