# Supplementary material for: Biochemical Competition Makes Fatty-Acid β-Oxidation Vulnerable to Substrate Overload
Source: PLoS Comput Biol. 2013 Aug 15;9(8):e1003186. doi: 10.1371/journal.pcbi.1003186 (PMC3744394; doi:10.1371/journal.pcbi.1003186)
Supplement: Protocol S2 — Time-simulation model of the FA β-oxidation. (PDF) [file pcbi.1003186.s006.pdf]

Protocol S2: Time-simulation model of the FA β-oxidation.

Kinetic model

Definitions of the various functions

CPT1[sf_, V_, Kms1_, Kms2_, Kmp1_, Kmp2_, Ki1_, Keq_, S1_, S2_, P1_, P2_, I1_, n_] :=

$$\frac{sf * V * \left(\frac{S1*S2}{Kms1*Kms2} - \frac{P1*P2}{Kms1*Kms2*Keq}\right)}{\left(1 + \frac{S1}{Kms1} + \frac{P1}{Kmp1} + \left(\frac{I1}{Ki1}\right)^{n}\right) * \left(1 + \frac{S2}{Kms2} + \frac{P2}{Kmp2}\right)}$$

CACT[Vf_, Vr_, Kms1_, Kms2_, Kmp1_, Kmp2_, Kis1_, Kip2_, Keq_, S1_, S2_, P1_, P2_] :=

$$\frac{Vf * \left(S1 * S2 - \frac{P1*P2}{Keq}\right)}{S1 * S2 + Kms2 * S1 + Kms1 * S2 * \left(1 + \frac{P2}{Kip2}\right) + \frac{Vf}{Vr*Keq} * \left(Kmp2 * P1 * \left(1 + \frac{S1}{Kis1}\right) + P2 * (Kmp1 + P1)\right)}$$

CPT2[sf_, V_, Kms1_, Kms2_, Kms3_, Kms4_, Kms5_, Kms6_, Kms7_, Kms8_, Kmp1_, Kmp2_, Kmp3_, Kmp4_, Kmp5_, Kmp6_, Kmp7_, Kmp8_, Keq_, S1_, S2_, S3_, S4_, S5_, S6_, S7_, S8_, P1_, P2_, P3_, P4_, P5_, P6_, P7_, P8_] :=

$$\left(sf * V * \left(\frac{S1 * S8}{Kms1 * Kms8} - \frac{P1 * P8}{Kms1 * Kms8 * Keq}\right)\right) \Big/ \left(\left(1 + \frac{S1}{Kms1} + \frac{P1}{Kmp1} + \frac{S2}{Kms2} + \frac{P2}{Kmp2} + \frac{S3}{Kms3} + \frac{P3}{Kmp3} + \frac{S4}{Kms4} + \frac{P4}{Kmp4} + \frac{S5}{Kms5} + \frac{P5}{Kmp5} + \frac{S6}{Kms6} + \frac{P6}{Kmp6} + \frac{S7}{Kms7} + \frac{P7}{Kmp7}\right) * \left(1 + \frac{S8}{Kms8} + \frac{P8}{Kmp8}\right)\right)$$

VLCAD[sf_, V_, Kms1_, Kms2_, Kms3_, Kms4_, Kmp1_, Kmp2_, Kmp3_, Kmp4_, Keq_, S1_, S2_, S3_, S4_, P1_, P2_, P3_, P4_] :=

$$\frac{sf * V * \left(\frac{S1*(S4-P4)}{Kms1*Kms4} - \frac{P1*P4}{Kms1*Kms4*Keq}\right)}{\left(1 + \frac{S1}{Kms1} + \frac{P1}{Kmp1} + \frac{S2}{Kms2} + \frac{P2}{Kmp2} + \frac{S3}{Kms3} + \frac{P3}{Kmp3}\right) * \left(1 + \frac{(S4-P4)}{Kms4} + \frac{P4}{Kmp4}\right)}$$

LCAD[sf_, V_, Kms1_, Kms2_, Kms3_, Kms4_, Kms5_, Kms6_, Kmp1_, Kmp2_, Kmp3_, Kmp4_, Kmp5_, Kmp6_, Keq_, S1_, S2_, S3_, S4_, S5_, S6_, P1_, P2_, P3_, P4_, P5_, P6_] :=

$$\frac{sf * V * \left(\frac{S1*(S6-P6)}{Kms1*Kms6} - \frac{P1*P6}{Kms1*Kms6*Keq}\right)}{\left(1 + \frac{S1}{Kms1} + \frac{P1}{Kmp1} + \frac{S2}{Kms2} + \frac{P2}{Kmp2} + \frac{S3}{Kms3} + \frac{P3}{Kmp3} + \frac{S4}{Kms4} + \frac{P4}{Kmp4} + \frac{S5}{Kms5} + \frac{P5}{Kmp5}\right) * \left(1 + \frac{(S6-P6)}{Kms6} + \frac{P6}{Kmp6}\right)}$$

MCAD[sf_, V_, Kms1_, Kms2_, Kms3_, Kms4_, Kms5_, Kms6_, Kmp1_, Kmp2_, Kmp3_, Kmp4_, Kmp5_, Kmp6_, Keq_, S1_, S2_, S3_, S4_, S5_, S6_, P1_, P2_, P3_, P4_, P5_, P6_] :=

$$\frac{sf * V * \left(\frac{S1*(S6-P6)}{Kms1*Kms6} - \frac{P1*P6}{Kms1*Kms6*Keq}\right)}{\left(1 + \frac{S1}{Kms1} + \frac{P1}{Kmp1} + \frac{S2}{Kms2} + \frac{P2}{Kmp2} + \frac{S3}{Kms3} + \frac{P3}{Kmp3} + \frac{S4}{Kms4} + \frac{P4}{Kmp4} + \frac{S5}{Kms5} + \frac{P5}{Kmp5}\right) * \left(1 + \frac{(S6-P6)}{Kms6} + \frac{P6}{Kmp6}\right)}$$

$$\text{SCAD[sf\_, V\_, Kms1\_, Kms2\_, Kms3\_, Kmp1\_, Kmp2\_, Kmp3\_, Keq\_, S1\_, S2\_, S3\_, P1\_, P2\_, P3\_]} := \frac{sf * V * \left(\frac{S1*(S3-P3)}{Kms1*Kms3} - \frac{P1*P3}{Kms1*Kms3*Keq}\right)}{\left(1 + \frac{S1}{Kms1} + \frac{P1}{Kmp1} + \frac{S2}{Kms2} + \frac{P2}{Kmp2}\right) * \left(1 + \frac{(S3-P3)}{Kms3} + \frac{P3}{Kmp3}\right)}$$

$$\text{CROT[sf\_, V\_, Kms1\_, Kms2\_, Kms3\_, Kms4\_, Kms5\_, Kms6\_, Kms7\_, Kmp1\_, Kmp2\_, Kmp3\_, Kmp4\_, Kmp5\_, Kmp6\_, Kmp7\_, Ki1\_, Keq\_, S1\_, S2\_, S3\_, S4\_, S5\_, S6\_, S7\_, P1\_, P2\_, P3\_, P4\_, P5\_, P6\_, P7\_, I1\_]} := \frac{sf * V * \left(\frac{S1}{Kms1} - \frac{P1}{Kms1*Keq}\right)}{1 + \frac{S1}{Kms1} + \frac{P1}{Kmp1} + \frac{S2}{Kms2} + \frac{P2}{Kmp2} + \frac{S3}{Kms3} + \frac{P3}{Kmp3} + \frac{S4}{Kms4} + \frac{P4}{Kmp4} + \frac{S5}{Kms5} + \frac{P5}{Kmp5} + \frac{S6}{Kms6} + \frac{P6}{Kmp6} + \frac{S7}{Kms7} + \frac{P7}{Kmp7} + \frac{I1}{Ki1}}$$

$$\text{MSCHAD[sf\_, V\_, Kms1\_, Kms2\_, Kms3\_, Kms4\_, Kms5\_, Kms6\_, Kms7\_, Kms8\_, Kmp1\_, Kmp2\_, Kmp3\_, Kmp4\_, Kmp5\_, Kmp6\_, Kmp7\_, Kmp8\_, Keq\_, S1\_, S2\_, S3\_, S4\_, S5\_, S6\_, S7\_, S8\_, P1\_, P2\_, P3\_, P4\_, P5\_, P6\_, P7\_, P8\_]} := \left(sf * V * \left(\frac{S1 * (S8 - P8)}{Kms1 * Kms8} - \frac{P1 * P8}{Kms1 * Kms8 * Keq}\right)\right) \Big/ \left(\left(1 + \frac{S1}{Kms1} + \frac{P1}{Kmp1} + \frac{S2}{Kms2} + \frac{P2}{Kmp2} + \frac{S3}{Kms3} + \frac{P3}{Kmp3} + \frac{S4}{Kms4} + \frac{P4}{Kmp4} + \frac{S5}{Kms5} + \frac{P5}{Kmp5} + \frac{S6}{Kms6} + \frac{P6}{Kmp6} + \frac{S7}{Kms7} + \frac{P7}{Kmp7}\right) * \left(1 + \frac{(S8 - P8)}{Kms8} + \frac{P8}{Kmp8}\right)\right)$$

$$\text{MCKATA[sf\_, V\_, Kms1\_, Kms2\_, Kms3\_, Kms4\_, Kms5\_, Kms6\_, Kms7\_, Kms8\_, Kmp1\_, Kmp2\_, Kmp3\_, Kmp4\_, Kmp5\_, Kmp6\_, Kmp7\_, Kmp8\_, Keq\_, S1\_, S2\_, S3\_, S4\_, S5\_, S6\_, S7\_, S8\_, P1\_, P2\_, P3\_, P4\_, P5\_, P6\_, P7\_, P8\_]} := \left(sf * V * \left(\frac{S1 * S8}{Kms1 * Kms8} - \frac{P1 * P8}{Kms1 * Kms8 * Keq}\right)\right) \Big/ \left(\left(1 + \frac{S1}{Kms1} + \frac{P1}{Kmp1} + \frac{S2}{Kms2} + \frac{P2}{Kmp2} + \frac{S3}{Kms3} + \frac{P3}{Kmp3} + \frac{S4}{Kms4} + \frac{P4}{Kmp4} + \frac{S5}{Kms5} + \frac{P5}{Kmp5} + \frac{S6}{Kms6} + \frac{P6}{Kmp6} + \frac{S7}{Kms7} + \frac{P7}{Kmp7} + \frac{P8}{Kmp8}\right) * \left(1 + \frac{S8}{Kms8} + \frac{P8}{Kmp8}\right)\right)$$

$$\text{MCKATB[sf\_, V\_, Kms1\_, Kms2\_, Kms3\_, Kms4\_, Kms5\_, Kms6\_, Kms7\_, Kms8\_, Kmp1\_, Kmp2\_, Kmp3\_, Kmp4\_, Kmp5\_, Kmp6\_, Kmp7\_, Kmp8\_, Keq\_, S1\_, S2\_, S3\_, S4\_, S5\_, S6\_, S7\_, S8\_, P1\_, P2\_, P3\_, P4\_, P5\_, P6\_, P7\_, P8\_]} := \left(sf * V * \left(\frac{S1 * S8}{Kms1 * Kms8} - \frac{P8 * P8}{Kms1 * Kms8 * Keq}\right)\right) \Big/ \left(\left(1 + \frac{S1}{Kms1} + \frac{P1}{Kmp1} + \frac{S2}{Kms2} + \frac{P2}{Kmp2} + \frac{S3}{Kms3} + \frac{P3}{Kmp3} + \frac{S4}{Kms4} + \frac{P4}{Kmp4} + \frac{S5}{Kms5} + \frac{P5}{Kmp5} + \frac{S6}{Kms6} + \frac{P6}{Kmp6} + \frac{S7}{Kms7} + \frac{P7}{Kmp7} + \frac{P8}{Kmp8}\right) * \left(1 + \frac{S8}{Kms8} + \frac{P8}{Kmp8}\right)\right)$$

```
MTP[sf_, V_, Kms1_, Kms2_, Kms3_, Kms4_, Kms5_, Kms7_, Kms8_, Kmp1_,
  Kmp2_, Kmp3_, Kmp4_, Kmp5_, Kmp6_, Kmp7_, Kmp8_, Ki1_, Keq_, S1_, S2_,
  S3_, S4_, S5_, S7_, S8_, P1_, P2_, P3_, P4_, P5_, P6_, P7_, P8_, I1_] :=
```

$$\left(sf * V * \left(\frac{S1 * (S7 - P7) * S8}{Kms1 * Kms7 * Kms8} - \frac{P1 * P7 * P8}{Kms1 * Kms7 * Kms8 * Keq}\right)\right) \Big/ \left(\left(1 + \frac{S1}{Kms1} + \frac{P1}{Kmp1} + \frac{S2}{Kms2} + \frac{P2}{Kmp2} + \frac{S3}{Kms3} + \frac{P3}{Kmp3} + \frac{S4}{Kms4} + \frac{P4}{Kmp4} + \frac{S5}{Kms5} + \frac{P5}{Kmp5} + \frac{P6}{Kmp6} + \frac{I1}{Ki1}\right) * \left(1 + \frac{(S7 - P7)}{Kms7} + \frac{P7}{Kmp7}\right) * \left(1 + \frac{S8}{Kms8} + \frac{P8}{Kmp8}\right)\right)$$

```
RES[Ks_, S_, K1_] := Ks * (S - K1)
```

Define the differential equations

Odes = {

$$C16AcylCarCYT'[t] == \frac{vcpt1C16 - vcactC16}{VCYT},$$

$$C16AcylCarMAT'[t] == \frac{vcactC16 - vcpt2C16}{VMAT},$$

$$C16AcylCoAMAT'[t] == \frac{vcpt2C16 - vvlcadC16 - vlcadC16}{VMAT},$$

$$C16EnoylCoAMAT'[t] == \frac{vvlcadC16 + vlcadC16 - vcrotC16 - vmtpC16}{VMAT},$$

$$C16HydroxyacylCoAMAT'[t] == \frac{vcrotC16 - vmschadC16}{VMAT},$$

$$C16KetoacylCoAMAT'[t] == \frac{vmschadC16 - vmckatC16}{VMAT},$$

$$C14AcylCarCYT'[t] == \frac{-vcactC14}{VCYT},$$

$$C14AcylCarMAT'[t] == \frac{vcactC14 - vcpt2C14}{VMAT},$$

$$C14AcylCoAMAT'[t] == \frac{vcpt2C14 + vmtpC16 + vmckatC16 - vvlcadC14 - vlcadC14}{VMAT},$$

$$C14EnoylCoAMAT'[t] == \frac{vvlcadC14 + vlcadC14 - vcrotC14 - vmtpC14}{VMAT},$$

$$C14HydroxyacylCoAMAT'[t] == \frac{vcrotC14 - vmschadC14}{VMAT},$$

$$C14KetoacylCoAMAT'[t] == \frac{vmschadC14 - vmckatC14}{VMAT},$$

$$C12AcylCarCYT'[t] == \frac{-vcactC12}{VCYT},$$

$$C12AcylCarMAT'[t] == \frac{vcactC12 - vcpt2C12}{VMAT},$$

$$C12AcylCoAMAT'[t] == \frac{1}{VMAT} (vcpt2C12 + vmtpC14 + vmckatC14 - vvlcadC12 - vlcadC12 - vmcadC12),$$

$$C12EnoylCoAMAT'[t] == \frac{vvlcadC12 + vlcadC12 + vmcadC12 - vcrotC12 - vmtpC12}{VMAT},$$

$$C12HydroxyacylCoAMAT'[t] == \frac{vcrotC12 - vmschadC12}{VMAT},$$

$$C12KetoacylCoAMAT'[t] == \frac{vmschadC12 - vmckatC12}{VMAT},$$

$$C10AcylCarCYT'[t] == \frac{-vcactC10}{VCYT},$$

$$C10AcylCarMAT'[t] == \frac{vcactC10 - vcpt2C10}{VMAT},$$

$$C10AcylCoAMAT'[t] == \frac{vcpt2C10 + vmtpC12 + vmckatC12 - vlcadC10 - vmcadC10}{VMAT},$$

$$C10EnoylCoAMAT'[t] == \frac{vlcadC10 + vmcadC10 - vcrotC10 - vmtpC10}{VMAT},$$

$$C10HydroxyacylCoAMAT'[t] == \frac{vcrotC10 - vmschadC10}{VMAT},$$

$$C10KetoacylCoAMAT'[t] == \frac{vmschadC10 - vmckatC10}{VMAT},$$

$$C8AcylCarCYT'[t] == \frac{-vcactC8}{VCYT},$$

$$C8AcylCarMAT'[t] == \frac{vcactC8 - vcpt2C8}{VMAT},$$

$$C8AcylCoAMAT'[t] == \frac{vcpt2C8 + vmtpC10 + vmckatC10 - vlcadC8 - vmcadC8}{VMAT},$$

$$C8EnoylCoAMAT'[t] == \frac{vlcadC8 + vmcadC8 - vcrotC8 - vmtpC8}{VMAT},$$

$$C8HydroxyacylCoAMAT'[t] == \frac{vcrotC8 - vmschadC8}{VMAT},$$

$$C8KetoacylCoAMAT'[t] == \frac{vmschadC8 - vmckatC8}{VMAT},$$

$$C6AcylCarCYT'[t] == \frac{-vcactC6}{VCYT},$$

$$C6AcylCarMAT'[t] == \frac{vcactC6 - vcpt2C6}{VMAT},$$

$$C6AcylCoAMAT'[t] == \frac{vcpt2C6 + vmtpC8 + vmckatC8 - vmcadC6 - vscadC6}{VMAT},$$

$$C6EnoylCoAMAT'[t] == \frac{vmcadC6 + vscadC6 - vcrotC6}{VMAT},$$

$$C6HydroxyacylCoAMAT'[t] == \frac{vcrotC6 - vmschadC6}{VMAT},$$

$$C6KetoacylCoAMAT'[t] == \frac{vmschadC6 - vmckatC6}{VMAT},$$

$$C4AcylCarCYT'[t] == \frac{-vcactC4}{VCYT},$$

$$C4AcylCarMAT'[t] == \frac{vcactC4 - vcpt2C4}{VMAT},$$

$$C4AcylCoAMAT'[t] == \frac{vcpt2C4 + vmckatC6 - vmcadC4 - vscadC4}{VMAT},$$

$$C4EnoylCoAMAT'[t] == \frac{vmcadC4 + vscadC4 - vcrotC4}{VMAT},$$

```
C4HydroxyacylCoAMAT'[t] == (vcrotC4 - vmschadC4)/VMAT,
C4AcetoacylCoAMAT'[t] == (vmschadC4 - vmckatC4)/VMAT,
AcetylCoAMAT'[t] ==
  1/VMAT (vmtpC16 + vmckatC16 + vmtpC14 + vmckatC14 + vmtpC12 + vmckatC12 + vmtpC10 +
     vmckatC10 + vmtpC8 + vmckatC8 + vmckatC6 + 2 * vmckatC4 - vacesink),
FADHMAT'[t] == 1/VMAT (vvlcadC16 + vvlcadC14 + vvlcadC12 + vlcadC16 +
     vlcadC14 + vlcadC12 + vlcadC10 + vlcadC8 + vmcadC12 + vmcadC10 +
     vmcadC8 + vmcadC6 + vmcadC4 + vscadC6 + vscadC4 - vfadhsink),
NADHMAT'[t] == 1/VMAT (vmtpC16 + vmtpC14 + vmtpC12 + vmtpC10 + vmtpC8 +
     vmschadC16 + vmschadC14 + vmschadC12 + vmschadC10 +
     vmschadC8 + vmschadC6 + vmschadC4 - vnadhsink)};

RateEqs = {vcpt1C16 → CPT1[sfcpt1C16, Vcpt1, Kmcpt1C16AcylCoACYT, Kmcpt1CarCYT,
     Kmcpt1C16AcylCarCYT, Kmcpt1CoACYT, Kicpt1MalCoACYT, Keqcpt1,
     C16AcylCoACYT, CarCYT, C16AcylCarCYT[t], CoACYT, MalCoACYT, ncpt1],
   vcactC16 → CACT[Vfcact, Vrcact, KmcactC16AcylCarCYT, KmcactCarMAT,
     KmcactC16AcylCarMAT, KmcactCarCYT, KicactC16AcylCarCYT, KicactCarCYT,
     Keqcact, C16AcylCarCYT[t], CarMAT, C16AcylCarMAT[t], CarCYT],
   vcactC14 → CACT[Vfcact, Vrcact, KmcactC14AcylCarCYT, KmcactCarMAT,
     KmcactC14AcylCarMAT, KmcactCarCYT, KicactC14AcylCarCYT, KicactCarCYT,
     Keqcact, C14AcylCarCYT[t], CarMAT, C14AcylCarMAT[t], CarCYT],
   vcactC12 → CACT[Vfcact, Vrcact, KmcactC12AcylCarCYT, KmcactCarMAT,
     KmcactC12AcylCarMAT, KmcactCarCYT, KicactC12AcylCarCYT, KicactCarCYT,
     Keqcact, C12AcylCarCYT[t], CarMAT, C12AcylCarMAT[t], CarCYT],
   vcactC10 → CACT[Vfcact, Vrcact, KmcactC10AcylCarCYT, KmcactCarMAT,
     KmcactC10AcylCarMAT, KmcactCarCYT, KicactC10AcylCarCYT, KicactCarCYT,
     Keqcact, C10AcylCarCYT[t], CarMAT, C10AcylCarMAT[t], CarCYT],
   vcactC8 → CACT[Vfcact, Vrcact, KmcactC8AcylCarCYT, KmcactCarMAT,
     KmcactC8AcylCarMAT, KmcactCarCYT, KicactC8AcylCarCYT, KicactCarCYT,
     Keqcact, C8AcylCarCYT[t], CarMAT, C8AcylCarMAT[t], CarCYT],
   vcactC6 → CACT[Vfcact, Vrcact, KmcactC6AcylCarCYT, KmcactCarMAT,
     KmcactC6AcylCarMAT, KmcactCarCYT, KicactC6AcylCarCYT, KicactCarCYT,
     Keqcact, C6AcylCarCYT[t], CarMAT, C6AcylCarMAT[t], CarCYT],
   vcactC4 → CACT[Vfcact, Vrcact, KmcactC4AcylCarCYT, KmcactCarMAT,
     KmcactC4AcylCarMAT, KmcactCarCYT, KicactC4AcylCarCYT, KicactCarCYT,
     Keqcact, C4AcylCarCYT[t], CarMAT, C4AcylCarMAT[t], CarCYT],
   vcpt2C16 → CPT2[sfcpt2C16, Vcpt2, Kmcpt2C16AcylCarMAT, Kmcpt2C14AcylCarMAT,
     Kmcpt2C12AcylCarMAT, Kmcpt2C10AcylCarMAT, Kmcpt2C8AcylCarMAT,
     Kmcpt2C6AcylCarMAT, Kmcpt2C4AcylCarMAT, Kmcpt2CoAMAT,
     Kmcpt2C16AcylCoAMAT, Kmcpt2C14AcylCoAMAT, Kmcpt2C12AcylCoAMAT,
     Kmcpt2C10AcylCoAMAT, Kmcpt2C8AcylCoAMAT, Kmcpt2C6AcylCoAMAT,
     Kmcpt2C4AcylCoAMAT, Kmcpt2CarMAT, Keqcpt2, C16AcylCarMAT[t],
     C14AcylCarMAT[t], C12AcylCarMAT[t], C10AcylCarMAT[t], C8AcylCarMAT[t],
     C6AcylCarMAT[t], C4AcylCarMAT[t], CoAMAT, C16AcylCoAMAT[t],
     C14AcylCoAMAT[t], C12AcylCoAMAT[t], C10AcylCoAMAT[t],
     C8AcylCoAMAT[t], C6AcylCoAMAT[t], C4AcylCoAMAT[t], CarMAT],
   vcpt2C14 → CPT2[sfcpt2C14, Vcpt2, Kmcpt2C14AcylCarMAT, Kmcpt2C16AcylCarMAT,
```

```
   Kmcpt2C12AcylCarMAT, Kmcpt2C10AcylCarMAT, Kmcpt2C8AcylCarMAT,
   Kmcpt2C6AcylCarMAT, Kmcpt2C4AcylCoAMAT, Kmcpt2CoAMAT,
   Kmcpt2C14AcylCoAMAT, Kmcpt2C16AcylCoAMAT, Kmcpt2C12AcylCoAMAT,
   Kmcpt2C10AcylCoAMAT, Kmcpt2C8AcylCoAMAT, Kmcpt2C6AcylCoAMAT,
   Kmcpt2C4AcylCoAMAT, Kmcpt2CarMAT, Keqcpt2, C14AcylCarMAT[t],
   C16AcylCarMAT[t], C12AcylCarMAT[t], C10AcylCarMAT[t], C8AcylCarMAT[t],
   C6AcylCarMAT[t], C4AcylCarMAT[t], CoAMAT, C14AcylCoAMAT[t],
   C16AcylCoAMAT[t], C12AcylCoAMAT[t], C10AcylCoAMAT[t],
   C8AcylCoAMAT[t], C6AcylCoAMAT[t], C4AcylCoAMAT[t], CarMAT],
vcpt2C12 → CPT2[sfcpt2C12, Vcpt2, Kmcpt2C12AcylCarMAT, Kmcpt2C16AcylCarMAT,
   Kmcpt2C14AcylCarMAT, Kmcpt2C10AcylCarMAT, Kmcpt2C8AcylCarMAT,
   Kmcpt2C6AcylCarMAT, Kmcpt2C4AcylCarMAT, Kmcpt2CoAMAT,
   Kmcpt2C12AcylCoAMAT, Kmcpt2C16AcylCoAMAT, Kmcpt2C14AcylCoAMAT,
   Kmcpt2C10AcylCoAMAT, Kmcpt2C8AcylCoAMAT, Kmcpt2C6AcylCoAMAT,
   Kmcpt2C4AcylCoAMAT, Kmcpt2CarMAT, Keqcpt2, C12AcylCarMAT[t],
   C16AcylCarMAT[t], C14AcylCarMAT[t], C10AcylCarMAT[t], C8AcylCarMAT[t],
   C6AcylCarMAT[t], C4AcylCarMAT[t], CoAMAT, C12AcylCoAMAT[t],
   C16AcylCoAMAT[t], C14AcylCoAMAT[t], C10AcylCoAMAT[t],
   C8AcylCoAMAT[t], C6AcylCoAMAT[t], C4AcylCoAMAT[t], CarMAT],
vcpt2C10 → CPT2[sfcpt2C10, Vcpt2, Kmcpt2C10AcylCarMAT, Kmcpt2C16AcylCarMAT,
   Kmcpt2C14AcylCarMAT, Kmcpt2C12AcylCarMAT, Kmcpt2C8AcylCarMAT,
   Kmcpt2C6AcylCarMAT, Kmcpt2C4AcylCarMAT, Kmcpt2CoAMAT,
   Kmcpt2C10AcylCoAMAT, Kmcpt2C16AcylCoAMAT, Kmcpt2C14AcylCoAMAT,
   Kmcpt2C12AcylCoAMAT, Kmcpt2C8AcylCoAMAT, Kmcpt2C6AcylCoAMAT,
   Kmcpt2C4AcylCoAMAT, Kmcpt2CarMAT, Keqcpt2, C10AcylCarMAT[t],
   C16AcylCarMAT[t], C14AcylCarMAT[t], C12AcylCarMAT[t], C8AcylCarMAT[t],
   C6AcylCarMAT[t], C4AcylCarMAT[t], CoAMAT, C10AcylCoAMAT[t],
   C16AcylCoAMAT[t], C14AcylCoAMAT[t], C12AcylCoAMAT[t],
   C8AcylCoAMAT[t], C6AcylCoAMAT[t], C4AcylCoAMAT[t], CarMAT],
vcpt2C8 → CPT2[sfcpt2C8, Vcpt2, Kmcpt2C8AcylCarMAT, Kmcpt2C16AcylCarMAT,
   Kmcpt2C14AcylCarMAT, Kmcpt2C12AcylCarMAT, Kmcpt2C10AcylCarMAT,
   Kmcpt2C6AcylCarMAT, Kmcpt2C4AcylCarMAT, Kmcpt2CoAMAT,
   Kmcpt2C8AcylCoAMAT, Kmcpt2C16AcylCoAMAT, Kmcpt2C14AcylCoAMAT,
   Kmcpt2C12AcylCoAMAT, Kmcpt2C10AcylCoAMAT, Kmcpt2C6AcylCoAMAT,
   Kmcpt2C4AcylCoAMAT, Kmcpt2CarMAT, Keqcpt2, C8AcylCarMAT[t],
   C16AcylCarMAT[t], C14AcylCarMAT[t], C12AcylCarMAT[t],
   C10AcylCarMAT[t], C6AcylCarMAT[t], C4AcylCarMAT[t], CoAMAT,
   C8AcylCoAMAT[t], C16AcylCoAMAT[t], C14AcylCoAMAT[t], C12AcylCoAMAT[t],
   C10AcylCoAMAT[t], C6AcylCoAMAT[t], C4AcylCoAMAT[t], CarMAT],
vcpt2C6 → CPT2[sfcpt2C6, Vcpt2, Kmcpt2C6AcylCarMAT, Kmcpt2C16AcylCarMAT,
   Kmcpt2C14AcylCarMAT, Kmcpt2C12AcylCarMAT, Kmcpt2C10AcylCarMAT,
   Kmcpt2C8AcylCarMAT, Kmcpt2C4AcylCarMAT, Kmcpt2CoAMAT,
   Kmcpt2C6AcylCoAMAT, Kmcpt2C16AcylCoAMAT, Kmcpt2C14AcylCoAMAT,
   Kmcpt2C12AcylCoAMAT, Kmcpt2C10AcylCoAMAT, Kmcpt2C8AcylCoAMAT,
   Kmcpt2C4AcylCoAMAT, Kmcpt2CarMAT, Keqcpt2, C6AcylCarMAT[t],
   C16AcylCarMAT[t], C14AcylCarMAT[t], C12AcylCarMAT[t],
   C10AcylCarMAT[t], C8AcylCarMAT[t], C4AcylCarMAT[t], CoAMAT,
   C6AcylCoAMAT[t], C16AcylCoAMAT[t], C14AcylCoAMAT[t], C12AcylCoAMAT[t],
   C10AcylCoAMAT[t], C8AcylCoAMAT[t], C4AcylCoAMAT[t], CarMAT],
vcpt2C4 → CPT2[sfcpt2C4, Vcpt2, Kmcpt2C4AcylCarMAT, Kmcpt2C16AcylCarMAT,
   Kmcpt2C14AcylCarMAT, Kmcpt2C12AcylCarMAT, Kmcpt2C10AcylCarMAT,
   Kmcpt2C8AcylCarMAT, Kmcpt2C6AcylCarMAT, Kmcpt2CoAMAT,
   Kmcpt2C4AcylCoAMAT, Kmcpt2C16AcylCoAMAT, Kmcpt2C14AcylCoAMAT,
```

```
    Kmcpt2C12AcylCoAMAT, Kmcpt2C10AcylCoAMAT, Kmcpt2C8AcylCoAMAT,
    Kmcpt2C6AcylCoAMAT, Kmcpt2CarMAT, Keqcpt2, C4AcylCarMAT[t],
    C16AcylCarMAT[t], C14AcylCarMAT[t], C12AcylCarMAT[t],
    C10AcylCarMAT[t], C8AcylCarMAT[t], C6AcylCarMAT[t], CoAMAT,
    C4AcylCoAMAT[t], C16AcylCoAMAT[t], C14AcylCoAMAT[t], C12AcylCoAMAT[t],
    C10AcylCoAMAT[t], C8AcylCoAMAT[t], C6AcylCoAMAT[t], CarMAT],
  vvlcadC16 → VLCAD[sfvlcadC16, Vvlcad, KmvlcadC16AcylCoAMAT,
    KmvlcadC14AcylCoAMAT, KmvlcadC12AcylCoAMAT, KmvlcadFAD,
    KmvlcadC16EnoylCoAMAT, KmvlcadC14EnoylCoAMAT,
    KmvlcadC12EnoylCoAMAT, KmvlcadFADH, Keqvlcad, C16AcylCoAMAT[t],
    C14AcylCoAMAT[t], C12AcylCoAMAT[t], FADtMAT, C16EnoylCoAMAT[t],
    C14EnoylCoAMAT[t], C12EnoylCoAMAT[t], FADHMAT[t]],
  vvlcadC14 → VLCAD[sfvlcadC14, Vvlcad, KmvlcadC14AcylCoAMAT,
    KmvlcadC16AcylCoAMAT, KmvlcadC12AcylCoAMAT, KmvlcadFAD,
    KmvlcadC14EnoylCoAMAT, KmvlcadC16EnoylCoAMAT,
    KmvlcadC12EnoylCoAMAT, KmvlcadFADH, Keqvlcad, C14AcylCoAMAT[t],
    C16AcylCoAMAT[t], C12AcylCoAMAT[t], FADtMAT, C14EnoylCoAMAT[t],
    C16EnoylCoAMAT[t], C12EnoylCoAMAT[t], FADHMAT[t]],
  vvlcadC12 → VLCAD[sfvlcadC12, Vvlcad, KmvlcadC12AcylCoAMAT,
    KmvlcadC16AcylCoAMAT, KmvlcadC14AcylCoAMAT, KmvlcadFAD,
    KmvlcadC12EnoylCoAMAT, KmvlcadC16EnoylCoAMAT,
    KmvlcadC14EnoylCoAMAT, KmvlcadFADH, Keqvlcad, C12AcylCoAMAT[t],
    C16AcylCoAMAT[t], C14AcylCoAMAT[t], FADtMAT, C12EnoylCoAMAT[t],
    C16EnoylCoAMAT[t], C14EnoylCoAMAT[t], FADHMAT[t]],
  vlcadC16 → LCAD[sflcadC16, Vlcad, KmlcadC16AcylCoAMAT, KmlcadC14AcylCoAMAT,
    KmlcadC12AcylCoAMAT, KmlcadC10AcylCoAMAT, KmlcadC8AcylCoAMAT, KmlcadFAD,
    KmlcadC16EnoylCoAMAT, KmlcadC14EnoylCoAMAT, KmlcadC12EnoylCoAMAT,
    KmlcadC10EnoylCoAMAT, KmlcadC8EnoylCoAMAT, KmlcadFADH, Keqlcad,
    C16AcylCoAMAT[t], C14AcylCoAMAT[t], C12AcylCoAMAT[t], C10AcylCoAMAT[t],
    C8AcylCoAMAT[t], FADtMAT, C16EnoylCoAMAT[t], C14EnoylCoAMAT[t],
    C12EnoylCoAMAT[t], C10EnoylCoAMAT[t], C8EnoylCoAMAT[t], FADHMAT[t]],
  vlcadC14 → LCAD[sflcadC14, Vlcad, KmlcadC14AcylCoAMAT, KmlcadC16AcylCoAMAT,
    KmlcadC12AcylCoAMAT, KmlcadC10AcylCoAMAT, KmlcadC8AcylCoAMAT, KmlcadFAD,
    KmlcadC14EnoylCoAMAT, KmlcadC16EnoylCoAMAT, KmlcadC12EnoylCoAMAT,
    KmlcadC10EnoylCoAMAT, KmlcadC8EnoylCoAMAT, KmlcadFADH, Keqlcad,
    C14AcylCoAMAT[t], C16AcylCoAMAT[t], C12AcylCoAMAT[t], C10AcylCoAMAT[t],
    C8AcylCoAMAT[t], FADtMAT, C14EnoylCoAMAT[t], C16EnoylCoAMAT[t],
    C12EnoylCoAMAT[t], C10EnoylCoAMAT[t], C8EnoylCoAMAT[t], FADHMAT[t]],
  vlcadC12 → LCAD[sflcadC12, Vlcad, KmlcadC12AcylCoAMAT, KmlcadC16AcylCoAMAT,
    KmlcadC14AcylCoAMAT, KmlcadC10AcylCoAMAT, KmlcadC8AcylCoAMAT, KmlcadFAD,
    KmlcadC12EnoylCoAMAT, KmlcadC16EnoylCoAMAT, KmlcadC14EnoylCoAMAT,
    KmlcadC10EnoylCoAMAT, KmlcadC8EnoylCoAMAT, KmlcadFADH, Keqlcad,
    C12AcylCoAMAT[t], C16AcylCoAMAT[t], C14AcylCoAMAT[t], C10AcylCoAMAT[t],
    C8AcylCoAMAT[t], FADtMAT, C14EnoylCoAMAT[t], C16EnoylCoAMAT[t],
    C14EnoylCoAMAT[t], C10EnoylCoAMAT[t], C8EnoylCoAMAT[t], FADHMAT[t]],
  vlcadC10 → LCAD[sflcadC10, Vlcad, KmlcadC10AcylCoAMAT, KmlcadC16AcylCoAMAT,
    KmlcadC14AcylCoAMAT, KmlcadC12AcylCoAMAT, KmlcadC8AcylCoAMAT, KmlcadFAD,
    KmlcadC10EnoylCoAMAT, KmlcadC16EnoylCoAMAT, KmlcadC14EnoylCoAMAT,
    KmlcadC12EnoylCoAMAT, KmlcadC8EnoylCoAMAT, KmlcadFADH, Keqlcad,
    C10AcylCoAMAT[t], C16AcylCoAMAT[t], C14AcylCoAMAT[t], C12AcylCoAMAT[t],
    C8AcylCoAMAT[t], FADtMAT, C10EnoylCoAMAT[t], C16EnoylCoAMAT[t],
    C14EnoylCoAMAT[t], C12EnoylCoAMAT[t], C8EnoylCoAMAT[t], FADHMAT[t]],
  vlcadC8 → LCAD[sflcadC8, Vlcad, KmlcadC8AcylCoAMAT, KmlcadC16AcylCoAMAT,
```

```
    KmlcadC14AcylCoAMAT, KmlcadC12AcylCoAMAT, KmlcadC10AcylCoAMAT, KmlcadFAD,
    KmlcadC8EnoylCoAMAT, KmlcadC16EnoylCoAMAT, KmlcadC14EnoylCoAMAT,
    KmlcadC12EnoylCoAMAT, KmlcadC10EnoylCoAMAT, KmlcadFADH, Keqlcad,
    C8AcylCoAMAT[t], C16AcylCoAMAT[t], C14AcylCoAMAT[t], C12AcylCoAMAT[t],
    C10AcylCoAMAT[t], FADtMAT, C8EnoylCoAMAT[t], C16EnoylCoAMAT[t],
    C14EnoylCoAMAT[t], C12EnoylCoAMAT[t], C10EnoylCoAMAT[t], FADHMAT[t]],
  vmcadC12 → MCAD[sfmcadC12, Vmcad, KmmcadC12AcylCoAMAT, KmmcadC10AcylCoAMAT,
    KmmcadC8AcylCoAMAT, KmmcadC6AcylCoAMAT, KmmcadC4AcylCoAMAT, KmmcadFAD,
    KmmcadC12EnoylCoAMAT, KmmcadC10EnoylCoAMAT, KmmcadC8EnoylCoAMAT,
    KmmcadC6EnoylCoAMAT, KmmcadC4EnoylCoAMAT, KmmcadFADH, Keqmcad,
    C12AcylCoAMAT[t], C10AcylCoAMAT[t], C8AcylCoAMAT[t], C6AcylCoAMAT[t],
    C4AcylCoAMAT[t], FADtMAT, C12EnoylCoAMAT[t], C10EnoylCoAMAT[t],
    C8EnoylCoAMAT[t], C6EnoylCoAMAT[t], C4EnoylCoAMAT[t], FADHMAT[t]],
  vmcadC10 → MCAD[sfmcadC10, Vmcad, KmmcadC10AcylCoAMAT, KmmcadC12AcylCoAMAT,
    KmmcadC8AcylCoAMAT, KmmcadC6AcylCoAMAT, KmmcadC4AcylCoAMAT, KmmcadFAD,
    KmmcadC10EnoylCoAMAT, KmmcadC12EnoylCoAMAT, KmmcadC8EnoylCoAMAT,
    KmmcadC6EnoylCoAMAT, KmmcadC4EnoylCoAMAT, KmmcadFADH, Keqmcad,
    C10AcylCoAMAT[t], C12AcylCoAMAT[t], C8AcylCoAMAT[t], C6AcylCoAMAT[t],
    C4AcylCoAMAT[t], FADtMAT, C10EnoylCoAMAT[t], C12EnoylCoAMAT[t],
    C8EnoylCoAMAT[t], C6EnoylCoAMAT[t], C4EnoylCoAMAT[t], FADHMAT[t]],
  vmcadC8 → MCAD[sfmcadC8, Vmcad, KmmcadC8AcylCoAMAT, KmmcadC12AcylCoAMAT,
    KmmcadC10AcylCoAMAT, KmmcadC6AcylCoAMAT, KmmcadC4AcylCoAMAT, KmmcadFAD,
    KmmcadC8EnoylCoAMAT, KmmcadC12EnoylCoAMAT, KmmcadC10EnoylCoAMAT,
    KmmcadC6EnoylCoAMAT, KmmcadC4EnoylCoAMAT, KmmcadFADH, Keqmcad,
    C8AcylCoAMAT[t], C12AcylCoAMAT[t], C10AcylCoAMAT[t], C6AcylCoAMAT[t],
    C4AcylCoAMAT[t], FADtMAT, C8EnoylCoAMAT[t], C12EnoylCoAMAT[t],
    C10EnoylCoAMAT[t], C6EnoylCoAMAT[t], C4EnoylCoAMAT[t], FADHMAT[t]],
  vmcadC6 → MCAD[sfmcadC6, Vmcad, KmmcadC6AcylCoAMAT, KmmcadC12AcylCoAMAT,
    KmmcadC10AcylCoAMAT, KmmcadC8AcylCoAMAT, KmmcadC4AcylCoAMAT, KmmcadFAD,
    KmmcadC6EnoylCoAMAT, KmmcadC12EnoylCoAMAT, KmmcadC10EnoylCoAMAT,
    KmmcadC8EnoylCoAMAT, KmmcadC4EnoylCoAMAT, KmmcadFADH, Keqmcad,
    C6AcylCoAMAT[t], C12AcylCoAMAT[t], C10AcylCoAMAT[t], C8AcylCoAMAT[t],
    C4AcylCoAMAT[t], FADtMAT, C6EnoylCoAMAT[t], C12EnoylCoAMAT[t],
    C10EnoylCoAMAT[t], C8EnoylCoAMAT[t], C4EnoylCoAMAT[t], FADHMAT[t]],
  vmcadC4 → MCAD[sfmcadC4, Vmcad, KmmcadC4AcylCoAMAT, KmmcadC12AcylCoAMAT,
    KmmcadC10AcylCoAMAT, KmmcadC8AcylCoAMAT, KmmcadC6AcylCoAMAT, KmmcadFAD,
    KmmcadC4EnoylCoAMAT, KmmcadC12EnoylCoAMAT, KmmcadC10EnoylCoAMAT,
    KmmcadC8EnoylCoAMAT, KmmcadC6EnoylCoAMAT, KmmcadFADH, Keqmcad,
    C4AcylCoAMAT[t], C12AcylCoAMAT[t], C10AcylCoAMAT[t], C8AcylCoAMAT[t],
    C6AcylCoAMAT[t], FADtMAT, C4EnoylCoAMAT[t], C12EnoylCoAMAT[t],
    C10EnoylCoAMAT[t], C8EnoylCoAMAT[t], C6EnoylCoAMAT[t], FADHMAT[t]],
  vscadC6 → SCAD[sfscadC6, Vscad, KmscadC6AcylCoAMAT, KmscadC4AcylCoAMAT,
    KmscadFAD, KmscadC6EnoylCoAMAT, KmscadC4EnoylCoAMAT,
    KmscadFADH, Keqscad, C6AcylCoAMAT[t], C4AcylCoAMAT[t],
    FADtMAT, C6EnoylCoAMAT[t], C4EnoylCoAMAT[t], FADHMAT[t]],
  vscadC4 → SCAD[sfscadC4, Vscad, KmscadC4AcylCoAMAT, KmscadC6AcylCoAMAT,
    KmscadFAD, KmscadC4EnoylCoAMAT, KmscadC6EnoylCoAMAT,
    KmscadFADH, Keqscad, C4AcylCoAMAT[t], C6AcylCoAMAT[t],
    FADtMAT, C4EnoylCoAMAT[t], C6EnoylCoAMAT[t], FADHMAT[t]],
  vcrotC16 → CROT[sfcrotC16, Vcrot, KmcrotC16EnoylCoAMAT, KmcrotC14EnoylCoAMAT,
    KmcrotC12EnoylCoAMAT, KmcrotC10EnoylCoAMAT, KmcrotC8EnoylCoAMAT,
    KmcrotC6EnoylCoAMAT, KmcrotC4EnoylCoAMAT, KmcrotC16HydroxyacylCoAMAT,
    KmcrotC14HydroxyacylCoAMAT, KmcrotC12HydroxyacylCoAMAT,
```

```
   KmcrotC10HydroxyacylCoAMAT, KmcrotC8HydroxyacylCoAMAT,
   KmcrotC6HydroxyacylCoAMAT, KmcrotC4HydroxyacylCoAMAT,
   KicrotC4AcetoacylCoA, Keqcrot, C16EnoylCoAMAT[t], C14EnoylCoAMAT[t],
   C12EnoylCoAMAT[t], C10EnoylCoAMAT[t], C8EnoylCoAMAT[t], C6EnoylCoAMAT[t],
   C4EnoylCoAMAT[t], C16HydroxyacylCoAMAT[t], C14HydroxyacylCoAMAT[t],
   C12HydroxyacylCoAMAT[t], C10HydroxyacylCoAMAT[t], C8HydroxyacylCoAMAT[t],
   C6HydroxyacylCoAMAT[t], C4HydroxyacylCoAMAT[t], C4AcetoacylCoAMAT[t]],
vcrotC14 → CROT[sfcrotC14, Vcrot, KmcrotC14EnoylCoAMAT, KmcrotC16EnoylCoAMAT,
   KmcrotC12EnoylCoAMAT, KmcrotC10EnoylCoAMAT, KmcrotC8EnoylCoAMAT,
   KmcrotC6EnoylCoAMAT, KmcrotC4EnoylCoAMAT, KmcrotC14HydroxyacylCoAMAT,
   KmcrotC16HydroxyacylCoAMAT, KmcrotC12HydroxyacylCoAMAT,
   KmcrotC10HydroxyacylCoAMAT, KmcrotC8HydroxyacylCoAMAT,
   KmcrotC6HydroxyacylCoAMAT, KmcrotC4HydroxyacylCoAMAT,
   KicrotC4AcetoacylCoA, Keqcrot, C14EnoylCoAMAT[t], C16EnoylCoAMAT[t],
   C12EnoylCoAMAT[t], C10EnoylCoAMAT[t], C8EnoylCoAMAT[t], C6EnoylCoAMAT[t],
   C4EnoylCoAMAT[t], C14HydroxyacylCoAMAT[t], C16HydroxyacylCoAMAT[t],
   C12HydroxyacylCoAMAT[t], C10HydroxyacylCoAMAT[t], C8HydroxyacylCoAMAT[t],
   C6HydroxyacylCoAMAT[t], C4HydroxyacylCoAMAT[t], C4AcetoacylCoAMAT[t]],
vcrotC12 → CROT[sfcrotC12, Vcrot, KmcrotC12EnoylCoAMAT, KmcrotC16EnoylCoAMAT,
   KmcrotC14EnoylCoAMAT, KmcrotC10EnoylCoAMAT, KmcrotC8EnoylCoAMAT,
   KmcrotC6EnoylCoAMAT, KmcrotC4EnoylCoAMAT, KmcrotC12HydroxyacylCoAMAT,
   KmcrotC16HydroxyacylCoAMAT, KmcrotC14HydroxyacylCoAMAT,
   KmcrotC10HydroxyacylCoAMAT, KmcrotC8HydroxyacylCoAMAT,
   KmcrotC6HydroxyacylCoAMAT, KmcrotC4HydroxyacylCoAMAT,
   KicrotC4AcetoacylCoA, Keqcrot, C12EnoylCoAMAT[t], C16EnoylCoAMAT[t],
   C14EnoylCoAMAT[t], C10EnoylCoAMAT[t], C8EnoylCoAMAT[t], C6EnoylCoAMAT[t],
   C4EnoylCoAMAT[t], C12HydroxyacylCoAMAT[t], C16HydroxyacylCoAMAT[t],
   C14HydroxyacylCoAMAT[t], C10HydroxyacylCoAMAT[t], C8HydroxyacylCoAMAT[t],
   C6HydroxyacylCoAMAT[t], C4HydroxyacylCoAMAT[t], C4AcetoacylCoAMAT[t]],
vcrotC10 → CROT[sfcrotC10, Vcrot, KmcrotC10EnoylCoAMAT, KmcrotC16EnoylCoAMAT,
   KmcrotC14EnoylCoAMAT, KmcrotC12EnoylCoAMAT, KmcrotC8EnoylCoAMAT,
   KmcrotC6EnoylCoAMAT, KmcrotC4EnoylCoAMAT, KmcrotC10HydroxyacylCoAMAT,
   KmcrotC16HydroxyacylCoAMAT, KmcrotC14HydroxyacylCoAMAT,
   KmcrotC12HydroxyacylCoAMAT, KmcrotC8HydroxyacylCoAMAT,
   KmcrotC6HydroxyacylCoAMAT, KmcrotC4HydroxyacylCoAMAT,
   KicrotC4AcetoacylCoA, Keqcrot, C10EnoylCoAMAT[t], C16EnoylCoAMAT[t],
   C14EnoylCoAMAT[t], C12EnoylCoAMAT[t], C8EnoylCoAMAT[t], C6EnoylCoAMAT[t],
   C4EnoylCoAMAT[t], C10HydroxyacylCoAMAT[t], C16HydroxyacylCoAMAT[t],
   C14HydroxyacylCoAMAT[t], C12HydroxyacylCoAMAT[t], C8HydroxyacylCoAMAT[t],
   C6HydroxyacylCoAMAT[t], C4HydroxyacylCoAMAT[t], C4AcetoacylCoAMAT[t]],
vcrotC8 → CROT[sfcrotC8, Vcrot, KmcrotC8EnoylCoAMAT, KmcrotC16EnoylCoAMAT,
   KmcrotC14EnoylCoAMAT, KmcrotC12EnoylCoAMAT, KmcrotC10EnoylCoAMAT,
   KmcrotC6EnoylCoAMAT, KmcrotC4EnoylCoAMAT, KmcrotC8HydroxyacylCoAMAT,
   KmcrotC16HydroxyacylCoAMAT, KmcrotC14HydroxyacylCoAMAT,
   KmcrotC12HydroxyacylCoAMAT, KmcrotC10HydroxyacylCoAMAT,
   KmcrotC6HydroxyacylCoAMAT, KmcrotC4HydroxyacylCoAMAT,
   KicrotC4AcetoacylCoA, Keqcrot, C8EnoylCoAMAT[t], C16EnoylCoAMAT[t],
   C14EnoylCoAMAT[t], C12EnoylCoAMAT[t], C10EnoylCoAMAT[t], C6EnoylCoAMAT[t],
   C4EnoylCoAMAT[t], C8HydroxyacylCoAMAT[t], C16HydroxyacylCoAMAT[t],
   C14HydroxyacylCoAMAT[t], C12HydroxyacylCoAMAT[t], C10HydroxyacylCoAMAT[t],
   C6HydroxyacylCoAMAT[t], C4HydroxyacylCoAMAT[t], C4AcetoacylCoAMAT[t]],
vcrotC6 → CROT[sfcrotC6, Vcrot, KmcrotC6EnoylCoAMAT, KmcrotC16EnoylCoAMAT,
   KmcrotC14EnoylCoAMAT, KmcrotC12EnoylCoAMAT, KmcrotC10EnoylCoAMAT,
```

```
    KmcrotC8EnoylCoAMAT, KmcrotC4EnoylCoAMAT, KmcrotC6HydroxyacylCoAMAT,
    KmcrotC16HydroxyacylCoAMAT, KmcrotC14HydroxyacylCoAMAT,
    KmcrotC12HydroxyacylCoAMAT, KmcrotC10HydroxyacylCoAMAT,
    KmcrotC8HydroxyacylCoAMAT, KmcrotC4HydroxyacylCoAMAT,
    KicrotC4AcetoacylCoA, Keqcrot, C6EnoylCoAMAT[t], C16EnoylCoAMAT[t],
    C14EnoylCoAMAT[t], C12EnoylCoAMAT[t], C10EnoylCoAMAT[t], C8EnoylCoAMAT[t],
    C4EnoylCoAMAT[t], C6HydroxyacylCoAMAT[t], C16HydroxyacylCoAMAT[t],
    C14HydroxyacylCoAMAT[t], C12HydroxyacylCoAMAT[t], C10HydroxyacylCoAMAT[t],
    C8HydroxyacylCoAMAT[t], C4HydroxyacylCoAMAT[t], C4AcetoacylCoAMAT[t]],
  vcrotC4 → CROT[sfcrotC4, Vcrot, KmcrotC4EnoylCoAMAT, KmcrotC16EnoylCoAMAT,
    KmcrotC14EnoylCoAMAT, KmcrotC12EnoylCoAMAT, KmcrotC10EnoylCoAMAT,
    KmcrotC8EnoylCoAMAT, KmcrotC6EnoylCoAMAT, KmcrotC4HydroxyacylCoAMAT,
    KmcrotC16HydroxyacylCoAMAT, KmcrotC14HydroxyacylCoAMAT,
    KmcrotC12HydroxyacylCoAMAT, KmcrotC10HydroxyacylCoAMAT,
    KmcrotC8HydroxyacylCoAMAT, KmcrotC6HydroxyacylCoAMAT,
    KicrotC4AcetoacylCoA, Keqcrot, C4EnoylCoAMAT[t], C16EnoylCoAMAT[t],
    C14EnoylCoAMAT[t], C12EnoylCoAMAT[t], C10EnoylCoAMAT[t], C8EnoylCoAMAT[t],
    C6EnoylCoAMAT[t], C4HydroxyacylCoAMAT[t], C16HydroxyacylCoAMAT[t],
    C14HydroxyacylCoAMAT[t], C12HydroxyacylCoAMAT[t], C10HydroxyacylCoAMAT[t],
    C8HydroxyacylCoAMAT[t], C6HydroxyacylCoAMAT[t], C4AcetoacylCoAMAT[t]],
  vmschadC16 → MSCHAD[sfmschadC16, Vmschad, KmmschadC16HydroxyacylCoAMAT,
    KmmschadC14HydroxyacylCoAMAT, KmmschadC12HydroxyacylCoAMAT,
    KmmschadC10HydroxyacylCoAMAT, KmmschadC8HydroxyacylCoAMAT,
    KmmschadC6HydroxyacylCoAMAT, KmmschadC4HydroxyacylCoAMAT,
    KmmschadNADMAT, KmmschadC16KetoacylCoAMAT, KmmschadC14KetoacylCoAMAT,
    KmmschadC12KetoacylCoAMAT, KmmschadC10KetoacylCoAMAT,
    KmmschadC8KetoacylCoAMAT, KmmschadC6KetoacylCoAMAT,
    KmmschadC4AcetoacylCoAMAT, KmmschadNADHMAT, Keqmschad,
    C16HydroxyacylCoAMAT[t], C14HydroxyacylCoAMAT[t],
    C12HydroxyacylCoAMAT[t], C10HydroxyacylCoAMAT[t],
    C8HydroxyacylCoAMAT[t], C6HydroxyacylCoAMAT[t], C4HydroxyacylCoAMAT[t],
    NADtMAT, C16KetoacylCoAMAT[t], C14KetoacylCoAMAT[t],
    C12KetoacylCoAMAT[t], C10KetoacylCoAMAT[t], C8KetoacylCoAMAT[t],
    C6KetoacylCoAMAT[t], C4AcetoacylCoAMAT[t], NADHMAT[t]],
  vmschadC14 → MSCHAD[sfmschadC14, Vmschad, KmmschadC14HydroxyacylCoAMAT,
    KmmschadC16HydroxyacylCoAMAT, KmmschadC12HydroxyacylCoAMAT,
    KmmschadC10HydroxyacylCoAMAT, KmmschadC8HydroxyacylCoAMAT,
    KmmschadC6HydroxyacylCoAMAT, KmmschadC4HydroxyacylCoAMAT,
    KmmschadNADMAT, KmmschadC14KetoacylCoAMAT, KmmschadC16KetoacylCoAMAT,
    KmmschadC12KetoacylCoAMAT, KmmschadC10KetoacylCoAMAT,
    KmmschadC8KetoacylCoAMAT, KmmschadC6KetoacylCoAMAT,
    KmmschadC4AcetoacylCoAMAT, KmmschadNADHMAT, Keqmschad,
    C14HydroxyacylCoAMAT[t], C16HydroxyacylCoAMAT[t],
    C12HydroxyacylCoAMAT[t], C10HydroxyacylCoAMAT[t],
    C8HydroxyacylCoAMAT[t], C6HydroxyacylCoAMAT[t], C4HydroxyacylCoAMAT[t],
    NADtMAT, C14KetoacylCoAMAT[t], C16KetoacylCoAMAT[t],
    C12KetoacylCoAMAT[t], C10KetoacylCoAMAT[t], C8KetoacylCoAMAT[t],
    C6KetoacylCoAMAT[t], C4AcetoacylCoAMAT[t], NADHMAT[t]],
  vmschadC12 → MSCHAD[sfmschadC12, Vmschad, KmmschadC12HydroxyacylCoAMAT,
    KmmschadC16HydroxyacylCoAMAT, KmmschadC14HydroxyacylCoAMAT,
    KmmschadC10HydroxyacylCoAMAT, KmmschadC8HydroxyacylCoAMAT,
    KmmschadC6HydroxyacylCoAMAT, KmmschadC4HydroxyacylCoAMAT,
    KmmschadNADMAT, KmmschadC12KetoacylCoAMAT, KmmschadC16KetoacylCoAMAT,
```

```
    KmmschadC14KetoacylCoAMAT, KmmschadC10KetoacylCoAMAT,
    KmmschadC8KetoacylCoAMAT, KmmschadC6KetoacylCoAMAT,
    KmmschadC4AcetoacylCoAMAT, KmmschadNADHMAT, Keqmschad,
    C12HydroxyacylCoAMAT[t], C16HydroxyacylCoAMAT[t],
    C14HydroxyacylCoAMAT[t], C10HydroxyacylCoAMAT[t],
    C8HydroxyacylCoAMAT[t], C6HydroxyacylCoAMAT[t], C4HydroxyacylCoAMAT[t],
    NADtMAT, C12KetoacylCoAMAT[t], C16KetoacylCoAMAT[t],
    C14KetoacylCoAMAT[t], C10KetoacylCoAMAT[t], C8KetoacylCoAMAT[t],
    C6KetoacylCoAMAT[t], C4AcetoacylCoAMAT[t], NADHMAT[t]],
vmschadC10 → MSCHAD[sfmschadC10, Vmschad, KmmschadC10HydroxyacylCoAMAT,
    KmmschadC16HydroxyacylCoAMAT, KmmschadC14HydroxyacylCoAMAT,
    KmmschadC12HydroxyacylCoAMAT, KmmschadC8HydroxyacylCoAMAT,
    KmmschadC6HydroxyacylCoAMAT, KmmschadC4HydroxyacylCoAMAT,
    KmmschadNADMAT, KmmschadC10KetoacylCoAMAT, KmmschadC16KetoacylCoAMAT,
    KmmschadC14KetoacylCoAMAT, KmmschadC12KetoacylCoAMAT,
    KmmschadC8KetoacylCoAMAT, KmmschadC6KetoacylCoAMAT,
    KmmschadC4AcetoacylCoAMAT, KmmschadNADHMAT, Keqmschad,
    C10HydroxyacylCoAMAT[t], C16HydroxyacylCoAMAT[t],
    C14HydroxyacylCoAMAT[t], C12HydroxyacylCoAMAT[t],
    C8HydroxyacylCoAMAT[t], C6HydroxyacylCoAMAT[t], C4HydroxyacylCoAMAT[t],
    NADtMAT, C10KetoacylCoAMAT[t], C16KetoacylCoAMAT[t],
    C14KetoacylCoAMAT[t], C12KetoacylCoAMAT[t], C8KetoacylCoAMAT[t],
    C6KetoacylCoAMAT[t], C4AcetoacylCoAMAT[t], NADHMAT[t]],
vmschadC8 → MSCHAD[sfmschadC8, Vmschad, KmmschadC8HydroxyacylCoAMAT,
    KmmschadC16HydroxyacylCoAMAT, KmmschadC14HydroxyacylCoAMAT,
    KmmschadC12HydroxyacylCoAMAT, KmmschadC10HydroxyacylCoAMAT,
    KmmschadC6HydroxyacylCoAMAT, KmmschadC4HydroxyacylCoAMAT,
    KmmschadNADMAT, KmmschadC8KetoacylCoAMAT, KmmschadC16KetoacylCoAMAT,
    KmmschadC14KetoacylCoAMAT, KmmschadC12KetoacylCoAMAT,
    KmmschadC10KetoacylCoAMAT, KmmschadC6KetoacylCoAMAT,
    KmmschadC4AcetoacylCoAMAT, KmmschadNADHMAT, Keqmschad,
    C8HydroxyacylCoAMAT[t], C16HydroxyacylCoAMAT[t],
    C14HydroxyacylCoAMAT[t], C12HydroxyacylCoAMAT[t],
    C10HydroxyacylCoAMAT[t], C6HydroxyacylCoAMAT[t], C4HydroxyacylCoAMAT[t],
    NADtMAT, C8KetoacylCoAMAT[t], C16KetoacylCoAMAT[t],
    C14KetoacylCoAMAT[t], C12KetoacylCoAMAT[t], C10KetoacylCoAMAT[t],
    C6KetoacylCoAMAT[t], C4AcetoacylCoAMAT[t], NADHMAT[t]],
vmschadC6 → MSCHAD[sfmschadC6, Vmschad, KmmschadC6HydroxyacylCoAMAT,
    KmmschadC16HydroxyacylCoAMAT, KmmschadC14HydroxyacylCoAMAT,
    KmmschadC12HydroxyacylCoAMAT, KmmschadC10HydroxyacylCoAMAT,
    KmmschadC8HydroxyacylCoAMAT, KmmschadC4HydroxyacylCoAMAT,
    KmmschadNADMAT, KmmschadC6KetoacylCoAMAT, KmmschadC16KetoacylCoAMAT,
    KmmschadC14KetoacylCoAMAT, KmmschadC12KetoacylCoAMAT,
    KmmschadC10KetoacylCoAMAT, KmmschadC8KetoacylCoAMAT,
    KmmschadC4AcetoacylCoAMAT, KmmschadNADHMAT, Keqmschad,
    C6HydroxyacylCoAMAT[t], C16HydroxyacylCoAMAT[t],
    C14HydroxyacylCoAMAT[t], C12HydroxyacylCoAMAT[t],
    C10HydroxyacylCoAMAT[t], C8HydroxyacylCoAMAT[t], C4HydroxyacylCoAMAT[t],
    NADtMAT, C6KetoacylCoAMAT[t], C16KetoacylCoAMAT[t],
    C14KetoacylCoAMAT[t], C12KetoacylCoAMAT[t], C10KetoacylCoAMAT[t],
    C8KetoacylCoAMAT[t], C4AcetoacylCoAMAT[t], NADHMAT[t]],
vmschadC4 → MSCHAD[sfmschadC4, Vmschad, KmmschadC4HydroxyacylCoAMAT,
    KmmschadC16HydroxyacylCoAMAT, KmmschadC14HydroxyacylCoAMAT,
```

```
KmmschadC12HydroxyacylCoAMAT, KmmschadC10HydroxyacylCoAMAT,
  KmmschadC8HydroxyacylCoAMAT, KmmschadC6HydroxyacylCoAMAT,
  KmmschadNADMAT, KmmschadC4AcetoacylCoAMAT, KmmschadC16KetoacylCoAMAT,
  KmmschadC14KetoacylCoAMAT, KmmschadC12KetoacylCoAMAT,
  KmmschadC10KetoacylCoAMAT, KmmschadC8KetoacylCoAMAT,
  KmmschadC6KetoacylCoAMAT, KmmschadNADHMAT, Keqmschad,
  C4HydroxyacylCoAMAT[t], C16HydroxyacylCoAMAT[t],
  C14HydroxyacylCoAMAT[t], C12HydroxyacylCoAMAT[t],
  C10HydroxyacylCoAMAT[t], C8HydroxyacylCoAMAT[t], C6HydroxyacylCoAMAT[t],
  NADtMAT, C4AcetoacylCoAMAT[t], C16KetoacylCoAMAT[t],
  C14KetoacylCoAMAT[t], C12KetoacylCoAMAT[t], C10KetoacylCoAMAT[t],
  C8KetoacylCoAMAT[t], C6KetoacylCoAMAT[t], NADHMAT[t]],
vmckatC16 → MCKATA[sfmckatC16, Vmckat, KmmckatC16KetoacylCoAMAT,
  KmmckatC14KetoacylCoAMAT, KmmckatC12KetoacylCoAMAT,
  KmmckatC10KetoacylCoAMAT, KmmckatC8KetoacylCoAMAT,
  KmmckatC6KetoacylCoAMAT, KmmckatC4AcetoacylCoAMAT, KmmckatCoAMAT,
  KmmckatC14AcylCoAMAT, KmmckatC16AcylCoAMAT, KmmckatC12AcylCoAMAT,
  KmmckatC10AcylCoAMAT, KmmckatC8AcylCoAMAT, KmmckatC6AcylCoAMAT,
  KmmckatC4AcylCoAMAT, KmmckatAcetylCoAMAT, Keqmckat, C16KetoacylCoAMAT[t],
  C14KetoacylCoAMAT[t], C12KetoacylCoAMAT[t], C10KetoacylCoAMAT[t],
  C8KetoacylCoAMAT[t], C6KetoacylCoAMAT[t], C4AcetoacylCoAMAT[t], CoAMAT,
  C14AcylCoAMAT[t], C16AcylCoAMAT[t], C12AcylCoAMAT[t], C10AcylCoAMAT[t],
  C8AcylCoAMAT[t], C6AcylCoAMAT[t], C4AcylCoAMAT[t], AcetylCoAMAT[t]],
vmckatC14 → MCKATA[sfmckatC14, Vmckat, KmmckatC14KetoacylCoAMAT,
  KmmckatC16KetoacylCoAMAT, KmmckatC12KetoacylCoAMAT,
  KmmckatC10KetoacylCoAMAT, KmmckatC8KetoacylCoAMAT,
  KmmckatC6KetoacylCoAMAT, KmmckatC4AcetoacylCoAMAT, KmmckatCoAMAT,
  KmmckatC12AcylCoAMAT, KmmckatC16AcylCoAMAT, KmmckatC14AcylCoAMAT,
  KmmckatC10AcylCoAMAT, KmmckatC8AcylCoAMAT, KmmckatC6AcylCoAMAT,
  KmmckatC4AcylCoAMAT, KmmckatAcetylCoAMAT, Keqmckat, C14KetoacylCoAMAT[t],
  C16KetoacylCoAMAT[t], C12KetoacylCoAMAT[t], C10KetoacylCoAMAT[t],
  C8KetoacylCoAMAT[t], C6KetoacylCoAMAT[t], C4AcetoacylCoAMAT[t], CoAMAT,
  C12AcylCoAMAT[t], C16AcylCoAMAT[t], C14AcylCoAMAT[t], C10AcylCoAMAT[t],
  C8AcylCoAMAT[t], C6AcylCoAMAT[t], C4AcylCoAMAT[t], AcetylCoAMAT[t]],
vmckatC12 → MCKATA[sfmckatC12, Vmckat, KmmckatC12KetoacylCoAMAT,
  KmmckatC16KetoacylCoAMAT, KmmckatC14KetoacylCoAMAT,
  KmmckatC10KetoacylCoAMAT, KmmckatC8KetoacylCoAMAT,
  KmmckatC6KetoacylCoAMAT, KmmckatC4AcetoacylCoAMAT, KmmckatCoAMAT,
  KmmckatC10AcylCoAMAT, KmmckatC16AcylCoAMAT, KmmckatC14AcylCoAMAT,
  KmmckatC12AcylCoAMAT, KmmckatC8AcylCoAMAT, KmmckatC6AcylCoAMAT,
  KmmckatC4AcylCoAMAT, KmmckatAcetylCoAMAT, Keqmckat, C12KetoacylCoAMAT[t],
  C16KetoacylCoAMAT[t], C14KetoacylCoAMAT[t], C10KetoacylCoAMAT[t],
  C8KetoacylCoAMAT[t], C6KetoacylCoAMAT[t], C4AcetoacylCoAMAT[t], CoAMAT,
  C10AcylCoAMAT[t], C16AcylCoAMAT[t], C14AcylCoAMAT[t], C12AcylCoAMAT[t],
  C8AcylCoAMAT[t], C6AcylCoAMAT[t], C4AcylCoAMAT[t], AcetylCoAMAT[t]],
vmckatC10 → MCKATA[sfmckatC10, Vmckat, KmmckatC10KetoacylCoAMAT,
  KmmckatC16KetoacylCoAMAT, KmmckatC14KetoacylCoAMAT,
  KmmckatC12KetoacylCoAMAT, KmmckatC8KetoacylCoAMAT,
  KmmckatC6KetoacylCoAMAT, KmmckatC4AcetoacylCoAMAT, KmmckatCoAMAT,
  KmmckatC8AcylCoAMAT, KmmckatC16AcylCoAMAT, KmmckatC14AcylCoAMAT,
  KmmckatC12AcylCoAMAT, KmmckatC10AcylCoAMAT, KmmckatC6AcylCoAMAT,
  KmmckatC4AcylCoAMAT, KmmckatAcetylCoAMAT, Keqmckat, C10KetoacylCoAMAT[t],
  C16KetoacylCoAMAT[t], C14KetoacylCoAMAT[t], C12KetoacylCoAMAT[t],
```

```
   C8KetoacylCoAMAT[t], C6KetoacylCoAMAT[t], C4AcetoacylCoAMAT[t], CoAMAT,
   C8AcylCoAMAT[t], C16AcylCoAMAT[t], C14AcylCoAMAT[t], C12AcylCoAMAT[t],
   C10AcylCoAMAT[t], C6AcylCoAMAT[t], C4AcylCoAMAT[t], AcetylCoAMAT[t]],
vmckatC8 → MCKATA[sfmckatC8, Vmckat, KmmckatC8KetoacylCoAMAT,
   KmmckatC16KetoacylCoAMAT, KmmckatC14KetoacylCoAMAT,
   KmmckatC12KetoacylCoAMAT, KmmckatC10KetoacylCoAMAT,
   KmmckatC6KetoacylCoAMAT, KmmckatC4AcetoacylCoAMAT, KmmckatCoAMAT,
   KmmckatC6AcylCoAMAT, KmmckatC16AcylCoAMAT, KmmckatC14AcylCoAMAT,
   KmmckatC12AcylCoAMAT, KmmckatC10AcylCoAMAT, KmmckatC8AcylCoAMAT,
   KmmckatC4AcylCoAMAT, KmmckatAcetylCoAMAT, Keqmckat, C8KetoacylCoAMAT[t],
   C16KetoacylCoAMAT[t], C14KetoacylCoAMAT[t], C12KetoacylCoAMAT[t],
   C10KetoacylCoAMAT[t], C6KetoacylCoAMAT[t], C4AcetoacylCoAMAT[t], CoAMAT,
   C6AcylCoAMAT[t], C16AcylCoAMAT[t], C14AcylCoAMAT[t], C12AcylCoAMAT[t],
   C10AcylCoAMAT[t], C8AcylCoAMAT[t], C4AcylCoAMAT[t], AcetylCoAMAT[t]],
vmckatC6 → MCKATA[sfmckatC6, Vmckat, KmmckatC6KetoacylCoAMAT,
   KmmckatC16KetoacylCoAMAT, KmmckatC14KetoacylCoAMAT,
   KmmckatC12KetoacylCoAMAT, KmmckatC10KetoacylCoAMAT,
   KmmckatC8KetoacylCoAMAT, KmmckatC4AcetoacylCoAMAT, KmmckatCoAMAT,
   KmmckatC4AcylCoAMAT, KmmckatC16AcylCoAMAT, KmmckatC14AcylCoAMAT,
   KmmckatC12AcylCoAMAT, KmmckatC10AcylCoAMAT, KmmckatC8AcylCoAMAT,
   KmmckatC6AcylCoAMAT, KmmckatAcetylCoAMAT, Keqmckat, C6KetoacylCoAMAT[t],
   C16KetoacylCoAMAT[t], C14KetoacylCoAMAT[t], C12KetoacylCoAMAT[t],
   C10KetoacylCoAMAT[t], C8KetoacylCoAMAT[t], C4AcetoacylCoAMAT[t], CoAMAT,
   C4AcylCoAMAT[t], C16AcylCoAMAT[t], C14AcylCoAMAT[t], C12AcylCoAMAT[t],
   C10AcylCoAMAT[t], C8AcylCoAMAT[t], C6AcylCoAMAT[t], AcetylCoAMAT[t]],
vmckatC4 → MCKATB[sfmckatC4, Vmckat, KmmckatC4AcetoacylCoAMAT,
   KmmckatC16KetoacylCoAMAT, KmmckatC14KetoacylCoAMAT,
   KmmckatC12KetoacylCoAMAT, KmmckatC10KetoacylCoAMAT,
   KmmckatC8KetoacylCoAMAT, KmmckatC6KetoacylCoAMAT, KmmckatCoAMAT,
   KmmckatC4AcylCoAMAT, KmmckatC16AcylCoAMAT, KmmckatC14AcylCoAMAT,
   KmmckatC12AcylCoAMAT, KmmckatC10AcylCoAMAT, KmmckatC8AcylCoAMAT,
   KmmckatC6AcylCoAMAT, KmmckatAcetylCoAMAT, Keqmckat, C4AcetoacylCoAMAT[t],
   C16KetoacylCoAMAT[t], C14KetoacylCoAMAT[t], C12KetoacylCoAMAT[t],
   C10KetoacylCoAMAT[t], C8KetoacylCoAMAT[t], C6KetoacylCoAMAT[t], CoAMAT,
   C4AcylCoAMAT[t], C16AcylCoAMAT[t], C14AcylCoAMAT[t], C12AcylCoAMAT[t],
   C10AcylCoAMAT[t], C8AcylCoAMAT[t], C6AcylCoAMAT[t], AcetylCoAMAT[t]],
vmtpC16 → MTP[sfmtpC16, Vmtp, KmmtpC16EnoylCoAMAT, KmmtpC14EnoylCoAMAT,
   KmmtpC12EnoylCoAMAT, KmmtpC10EnoylCoAMAT, KmmtpC8EnoylCoAMAT,
   KmmtpNADMAT, KmmtpCoAMAT, KmmtpC14AcylCoAMAT, KmmtpC16AcylCoAMAT,
   KmmtpC12AcylCoAMAT, KmmtpC10AcylCoAMAT, KmmtpC8AcylCoAMAT,
   KmmtpC6AcylCoAMAT, KmmtpNADHMAT, KmmtpAcetylCoAMAT, KicrotC4AcetoacylCoA,
   Keqmtp, C16EnoylCoAMAT[t], C14EnoylCoAMAT[t], C12EnoylCoAMAT[t],
   C10EnoylCoAMAT[t], C8EnoylCoAMAT[t], NADtMAT, CoAMAT, C14AcylCoAMAT[t],
   C16AcylCoAMAT[t], C12AcylCoAMAT[t], C10AcylCoAMAT[t], C8AcylCoAMAT[t],
   C6AcylCoAMAT[t], NADHMAT[t], AcetylCoAMAT[t], C4AcetoacylCoAMAT[t]],
vmtpC14 → MTP[sfmtpC14, Vmtp, KmmtpC14EnoylCoAMAT, KmmtpC16EnoylCoAMAT,
   KmmtpC12EnoylCoAMAT, KmmtpC10EnoylCoAMAT, KmmtpC8EnoylCoAMAT,
   KmmtpNADMAT, KmmtpCoAMAT, KmmtpC12AcylCoAMAT, KmmtpC16AcylCoAMAT,
   KmmtpC14AcylCoAMAT, KmmtpC10AcylCoAMAT, KmmtpC8AcylCoAMAT,
   KmmtpC6AcylCoAMAT, KmmtpNADHMAT, KmmtpAcetylCoAMAT, KicrotC4AcetoacylCoA,
   Keqmtp, C14EnoylCoAMAT[t], C16EnoylCoAMAT[t], C12EnoylCoAMAT[t],
   C10EnoylCoAMAT[t], C8EnoylCoAMAT[t], NADtMAT, CoAMAT, C12AcylCoAMAT[t],
   C16AcylCoAMAT[t], C14AcylCoAMAT[t], C10AcylCoAMAT[t], C8AcylCoAMAT[t],
```

```
      C6AcylCoAMAT[t], NADHMAT[t], AcetylCoAMAT[t], C4AcetoacylCoAMAT[t]],
    vmtpC12 → MTP[sfmtpC12, Vmtp, KmmtpC12EnoylCoAMAT, KmmtpC16EnoylCoAMAT,
      KmmtpC14EnoylCoAMAT, KmmtpC10EnoylCoAMAT, KmmtpC8EnoylCoAMAT,
      KmmtpNADMAT, KmmtpCoAMAT, KmmtpC10AcylCoAMAT, KmmtpC16AcylCoAMAT,
      KmmtpC14AcylCoAMAT, KmmtpC12AcylCoAMAT, KmmtpC8AcylCoAMAT,
      KmmtpC6AcylCoAMAT, KmmtpNADHMAT, KmmtpAcetylCoAMAT, KicrotC4AcetoacylCoA,
      Keqmtp, C12EnoylCoAMAT[t], C16EnoylCoAMAT[t], C14EnoylCoAMAT[t],
      C10EnoylCoAMAT[t], C8EnoylCoAMAT[t], NADtMAT, CoAMAT, C10AcylCoAMAT[t],
      C16AcylCoAMAT[t], C14AcylCoAMAT[t], C12AcylCoAMAT[t], C8AcylCoAMAT[t],
      C6AcylCoAMAT[t], NADHMAT[t], AcetylCoAMAT[t], C4AcetoacylCoAMAT[t]],
    vmtpC10 → MTP[sfmtpC10, Vmtp, KmmtpC10EnoylCoAMAT, KmmtpC16EnoylCoAMAT,
      KmmtpC14EnoylCoAMAT, KmmtpC12EnoylCoAMAT, KmmtpC8EnoylCoAMAT,
      KmmtpNADMAT, KmmtpCoAMAT, KmmtpC8AcylCoAMAT, KmmtpC16AcylCoAMAT,
      KmmtpC14AcylCoAMAT, KmmtpC12AcylCoAMAT, KmmtpC10AcylCoAMAT,
      KmmtpC6AcylCoAMAT, KmmtpNADHMAT, KmmtpAcetylCoAMAT, KicrotC4AcetoacylCoA,
      Keqmtp, C10EnoylCoAMAT[t], C16EnoylCoAMAT[t], C14EnoylCoAMAT[t],
      C12EnoylCoAMAT[t], C8EnoylCoAMAT[t], NADtMAT, CoAMAT, C8AcylCoAMAT[t],
      C16AcylCoAMAT[t], C14AcylCoAMAT[t], C12AcylCoAMAT[t], C10AcylCoAMAT[t],
      C6AcylCoAMAT[t], NADHMAT[t], AcetylCoAMAT[t], C4AcetoacylCoAMAT[t]],
    vmtpC8 → MTP[sfmtpC8, Vmtp, KmmtpC8EnoylCoAMAT, KmmtpC16EnoylCoAMAT,
      KmmtpC14EnoylCoAMAT, KmmtpC12EnoylCoAMAT, KmmtpC10EnoylCoAMAT,
      KmmtpNADMAT, KmmtpCoAMAT, KmmtpC6AcylCoAMAT, KmmtpC16AcylCoAMAT,
      KmmtpC14AcylCoAMAT, KmmtpC12AcylCoAMAT, KmmtpC10AcylCoAMAT,
      KmmtpC8AcylCoAMAT, KmmtpNADHMAT, KmmtpAcetylCoAMAT, KicrotC4AcetoacylCoA,
      Keqmtp, C8EnoylCoAMAT[t], C16EnoylCoAMAT[t], C14EnoylCoAMAT[t],
      C12EnoylCoAMAT[t], C10EnoylCoAMAT[t], NADtMAT, CoAMAT, C6AcylCoAMAT[t],
      C16AcylCoAMAT[t], C14AcylCoAMAT[t], C12AcylCoAMAT[t], C10AcylCoAMAT[t],
      C8AcylCoAMAT[t], NADHMAT[t], AcetylCoAMAT[t], C4AcetoacylCoAMAT[t]],
    vacesink → RES[Ksacesink, AcetylCoAMAT[t], K1acesink],
    vfadhsink → RES[Ksfadhsink, FADHMAT[t], K1fadhsink],
    vnadhsink → RES[Ksnadhsink, NADHMAT[t], K1nadhsink]};

CoAMATX = {CoAMAT → CoAMATt - C16AcylCoAMAT[t] - C16EnoylCoAMAT[t] -
      C16HydroxyacylCoAMAT[t] - C16KetoacylCoAMAT[t] - C14AcylCoAMAT[t] -
      C14EnoylCoAMAT[t] - C14HydroxyacylCoAMAT[t] - C14KetoacylCoAMAT[t] -
      C12AcylCoAMAT[t] - C12EnoylCoAMAT[t] - C12HydroxyacylCoAMAT[t] -
      C12KetoacylCoAMAT[t] - C10AcylCoAMAT[t] - C10EnoylCoAMAT[t] -
      C10HydroxyacylCoAMAT[t] - C10KetoacylCoAMAT[t] - C8AcylCoAMAT[t] -
      C8EnoylCoAMAT[t] - C8HydroxyacylCoAMAT[t] - C8KetoacylCoAMAT[t] -
      C6AcylCoAMAT[t] - C6EnoylCoAMAT[t] - C6HydroxyacylCoAMAT[t] -
      C6KetoacylCoAMAT[t] - C4AcylCoAMAT[t] - C4EnoylCoAMAT[t] -
      C4HydroxyacylCoAMAT[t] - C4AcetoacylCoAMAT[t] - AcetylCoAMAT[t]};

Parm = {
    sfcpt1C16 → 1, Vcpt1 → 0.012, Kmcpt1C16AcylCoACYT → 13.8,
    Kmcpt1CarCYT → 125, Kmcpt1C16AcylCarCYT → 136, Kmcpt1CoACYT → 40.7,
    Kicpt1MalCoACYT → 9.1, Keqcpt1 → 0.45, ncpt1 → 2.4799,
    Vfcact → 0.42, Vrcact → 0.42, KmcactC16AcylCarCYT → 15,
    KmcactC14AcylCarCYT → 15, KmcactC12AcylCarCYT → 15, KmcactC10AcylCarCYT → 15,
    KmcactC8AcylCarCYT → 15, KmcactC6AcylCarCYT → 15, KmcactC4AcylCarCYT → 15,
    KmcactCarMAT → 130, KmcactC16AcylCarMAT → 15, KmcactC14AcylCarMAT → 15,
    KmcactC12AcylCarMAT → 15, KmcactC10AcylCarMAT → 15, KmcactC8AcylCarMAT → 15,
    KmcactC6AcylCarMAT → 15, KmcactC4AcylCarMAT → 15, KmcactCarCYT → 130,
```

```
KicactC16AcylCarCYT → 56, KicactC14AcylCarCYT → 56, KicactC12AcylCarCYT → 56,
KicactC10AcylCarCYT → 56, KicactC8AcylCarCYT → 56, KicactC6AcylCarCYT → 56,
KicactC4AcylCarCYT → 56, KicactCarCYT → 200, Keqcact → 1,
sfcpt2C16 → 0.85, sfcpt2C14 → 1, sfcpt2C12 → 0.95, sfcpt2C10 → 0.95,
sfcpt2C8 → 0.35, sfcpt2C6 → 0.15, sfcpt2C4 → 0.01, Vcpt2 → 0.391,
Kmcpt2C16AcylCarMAT → 51, Kmcpt2C14AcylCarMAT → 51, Kmcpt2C12AcylCarMAT → 51,
Kmcpt2C10AcylCarMAT → 51, Kmcpt2C8AcylCarMAT → 51, Kmcpt2C6AcylCarMAT → 51,
Kmcpt2C4AcylCarMAT → 51, Kmcpt2CoAMAT → 30, Kmcpt2C16AcylCoAMAT → 38,
Kmcpt2C14AcylCoAMAT → 38, Kmcpt2C12AcylCoAMAT → 38,
Kmcpt2C10AcylCoAMAT → 38, Kmcpt2C8AcylCoAMAT → 38, Kmcpt2C6AcylCoAMAT → 1000,
Kmcpt2C4AcylCoAMAT → 1 000 000, Kmcpt2CarMAT → 350, Keqcpt2 → 2.22,
sfvlcadC16 → 1, sfvlcadC14 → 0.42, sfvlcadC12 → 0.11, Vvlcad → 0.008,
KmvlcadC16AcylCoAMAT → 6.5, KmvlcadC14AcylCoAMAT → 4,
KmvlcadC12AcylCoAMAT → 2.7, KmvlcadFAD → 0.12,
KmvlcadC16EnoylCoAMAT → 1.08, KmvlcadC14EnoylCoAMAT → 1.08,
KmvlcadC12EnoylCoAMAT → 1.08, KmvlcadFADH → 24.2, Keqvlcad → 6,
sflcadC16 → 0.9, sflcadC14 → 1, sflcadC12 → 0.9, sflcadC10 → 0.75,
sflcadC8 → 0.4, Vlcad → 0.01, KmlcadC16AcylCoAMAT → 2.5,
KmlcadC14AcylCoAMAT → 7.4, KmlcadC12AcylCoAMAT → 9,
KmlcadC10AcylCoAMAT → 24.3, KmlcadC8AcylCoAMAT → 123, KmlcadFAD → 0.12,
KmlcadC16EnoylCoAMAT → 1.08, KmlcadC14EnoylCoAMAT → 1.08,
KmlcadC12EnoylCoAMAT → 1.08, KmlcadC10EnoylCoAMAT → 1.08,
KmlcadC8EnoylCoAMAT → 1.08, KmlcadFADH → 24.2, Keqlcad → 6,
sfmcadC12 → 0.38, sfmcadC10 → 0.8, sfmcadC8 → 0.87, sfmcadC6 → 1,
sfmcadC4 → 0.12, Vmcad → 0.081, KmmcadC12AcylCoAMAT → 5.7,
KmmcadC10AcylCoAMAT → 5.4, KmmcadC8AcylCoAMAT → 4,
KmmcadC6AcylCoAMAT → 9.4, KmmcadC4AcylCoAMAT → 135, KmmcadFAD → 0.12,
KmmcadC12EnoylCoAMAT → 1.08, KmmcadC10EnoylCoAMAT → 1.08,
KmmcadC8EnoylCoAMAT → 1.08, KmmcadC6EnoylCoAMAT → 1.08,
KmmcadC4EnoylCoAMAT → 1.08, KmmcadFADH → 24.2, Keqmcad → 6,
sfscadC6 → 0.3, sfscadC4 → 1, Vscad → 0.081, KmscadC6AcylCoAMAT → 285,
KmscadC4AcylCoAMAT → 10.7, KmscadFAD → 0.12, KmscadC6EnoylCoAMAT → 1.08,
KmscadC4EnoylCoAMAT → 1.08, KmscadFADH → 24.2, Keqscad -> 6,
sfcrotC16 → 0.13, sfcrotC14 → 0.2, sfcrotC12 → 0.25, sfcrotC10 → 0.33,
sfcrotC8 → 0.58, sfcrotC6 → 0.83, sfcrotC4 → 1, Vcrot → 3.6,
KmcrotC16EnoylCoAMAT → 150, KmcrotC14EnoylCoAMAT → 100,
KmcrotC12EnoylCoAMAT → 25, KmcrotC10EnoylCoAMAT → 25,
KmcrotC8EnoylCoAMAT → 25, KmcrotC6EnoylCoAMAT → 25, KmcrotC4EnoylCoAMAT → 40,
KmcrotC16HydroxyacylCoAMAT → 45, KmcrotC14HydroxyacylCoAMAT → 45,
KmcrotC12HydroxyacylCoAMAT → 45, KmcrotC10HydroxyacylCoAMAT → 45,
KmcrotC8HydroxyacylCoAMAT → 45, KmcrotC6HydroxyacylCoAMAT → 45,
KmcrotC4HydroxyacylCoAMAT → 45, KicrotC4AcetoacylCoA → 1.6, Keqcrot → 3.13,
sfmschadC16 → 0.6, sfmschadC14 → 0.5, sfmschadC12 → 0.43,
sfmschadC10 → 0.64, sfmschadC8 → 0.89, sfmschadC6 → 1,
sfmschadC4 → 0.67, Vmschad → 1, KmmschadC16HydroxyacylCoAMAT → 1.5,
KmmschadC14HydroxyacylCoAMAT → 1.8, KmmschadC12HydroxyacylCoAMAT → 3.7,
KmmschadC10HydroxyacylCoAMAT → 8.8, KmmschadC8HydroxyacylCoAMAT → 16.3,
KmmschadC6HydroxyacylCoAMAT → 28.6, KmmschadC4HydroxyacylCoAMAT → 69.9,
KmmschadNADMAT → 58.5, KmmschadC16KetoacylCoAMAT → 1.4,
KmmschadC14KetoacylCoAMAT → 1.4, KmmschadC12KetoacylCoAMAT → 1.6,
KmmschadC10KetoacylCoAMAT → 2.3, KmmschadC8KetoacylCoAMAT → 4.1,
KmmschadC6KetoacylCoAMAT → 5.8, KmmschadC4AcetoacylCoAMAT → 16.9,
KmmschadNADHMAT → 5.4, Keqmschad → 2.17 * 10^-4,
```

```
    sfmckatC16 → 0, sfmckatC14 → 0.2, sfmckatC12 → 0.38, sfmckatC10 → 0.65,
    sfmckatC8 → 0.81, sfmckatC6 → 1, sfmckatC4 → 0.49, Vmckat → 0.377,
    KmmckatC16KetoacylCoAMAT → 1.1, KmmckatC14KetoacylCoAMAT → 1.2,
    KmmckatC12KetoacylCoAMAT → 1.3, KmmckatC10KetoacylCoAMAT → 2.1,
    KmmckatC8KetoacylCoAMAT → 3.2, KmmckatC6KetoacylCoAMAT → 6.7,
    KmmckatC4AcetoacylCoAMAT → 12.4, KmmckatCoAMAT → 26.6,
    KmmckatC14AcylCoAMAT → 13.83, KmmckatC16AcylCoAMAT → 13.83,
    KmmckatC12AcylCoAMAT → 13.83, KmmckatC10AcylCoAMAT → 13.83,
    KmmckatC8AcylCoAMAT → 13.83, KmmckatC6AcylCoAMAT → 13.83,
    KmmckatC4AcylCoAMAT → 13.83, KmmckatAcetylCoAMAT → 30, Keqmckat → 1051,
    sfmtpC16 → 1, sfmtpC14 → 0.9, sfmtpC12 → 0.81, sfmtpC10 → 0.73,
    sfmtpC8 → 0.34, Vmtp → 2.84, KmmtpC16EnoylCoAMAT → 25,
    KmmtpC14EnoylCoAMAT → 25, KmmtpC12EnoylCoAMAT → 25,
    KmmtpC10EnoylCoAMAT → 25, KmmtpC8EnoylCoAMAT → 25, KmmtpNADMAT → 60,
    KmmtpCoAMAT → 30, KmmtpC14AcylCoAMAT → 13.83, KmmtpC16AcylCoAMAT → 13.83,
    KmmtpC12AcylCoAMAT → 13.83, KmmtpC10AcylCoAMAT → 13.83,
    KmmtpC8AcylCoAMAT → 13.83, KmmtpC6AcylCoAMAT → 13.83,
    KmmtpNADHMAT → 50, KmmtpAcetylCoAMAT → 30, Keqmtp → 0.71,
    Ksacesink → 6 000 000, K1acesink → 30, Ksfadhsink → 6 000 000,
    K1fadhsink → 0.46, Ksnadhsink → 6 000 000, K1nadhsink → 16,
    C16AcylCoACYT → x, CarCYT → 400, CoACYT → 140, MalCoACYT → 0,
    CarMAT -> 950, FADtMAT → 0.77, NADtMAT → 250, CoAMATt → 5000,
    VCYT → 10 * 10^-3, VMAT → 1.8 * 10^-6};

InitialConditions = {
    C16AcylCarCYT[0] == 0.171, C16AcylCarMAT[0] == 0, C16AcylCoAMAT[0] == 0,
    C16EnoylCoAMAT[0] == 0, C16HydroxyacylCoAMAT[0] == 0, C16KetoacylCoAMAT[0] == 0,
    C14AcylCarCYT[0] == 0.023, C14AcylCarMAT[0] == 0, C14AcylCoAMAT[0] == 0,
    C14EnoylCoAMAT[0] == 0, C14HydroxyacylCoAMAT[0] == 0, C14KetoacylCoAMAT[0] == 0,
    C12AcylCarCYT[0] == 0.110, C12AcylCarMAT[0] == 0, C12AcylCoAMAT[0] == 0,
    C12EnoylCoAMAT[0] == 0, C12HydroxyacylCoAMAT[0] == 0, C12KetoacylCoAMAT[0] == 0,
    C10AcylCarCYT[0] == 0.019, C10AcylCarMAT[0] == 0, C10AcylCoAMAT[0] == 0,
    C10EnoylCoAMAT[0] == 0, C10HydroxyacylCoAMAT[0] == 0, C10KetoacylCoAMAT[0] == 0,
    C8AcylCarCYT[0] == 0.052, C8AcylCarMAT[0] == 0, C8AcylCoAMAT[0] == 0,
    C8EnoylCoAMAT[0] == 0, C8HydroxyacylCoAMAT[0] == 0, C8KetoacylCoAMAT[0] == 0,
    C6AcylCarCYT[0] == 0.017, C6AcylCarMAT[0] == 0, C6AcylCoAMAT[0] == 0,
    C6EnoylCoAMAT[0] == 0, C6HydroxyacylCoAMAT[0] == 0, C6KetoacylCoAMAT[0] == 0,
    C4AcylCarCYT[0] == 0.008, C4AcylCarMAT[0] == 0, C4AcylCoAMAT[0] == 0,
    C4EnoylCoAMAT[0] == 0, C4HydroxyacylCoAMAT[0] == 0, C4AcetoacylCoAMAT[0] == 0,
    AcetylCoAMAT[0] == 30, FADHMAT[0] == 0.46, NADHMAT[0] == 16};

Vars = {
    C16AcylCarCYT, C16AcylCarMAT, C16AcylCoAMAT,
    C16EnoylCoAMAT, C16HydroxyacylCoAMAT, C16KetoacylCoAMAT,
    C14AcylCarCYT, C14AcylCarMAT, C14AcylCoAMAT, C14EnoylCoAMAT,
    C14HydroxyacylCoAMAT, C14KetoacylCoAMAT,
    C12AcylCarCYT, C12AcylCarMAT, C12AcylCoAMAT, C12EnoylCoAMAT,
    C12HydroxyacylCoAMAT, C12KetoacylCoAMAT,
    C10AcylCarCYT, C10AcylCarMAT, C10AcylCoAMAT, C10EnoylCoAMAT,
    C10HydroxyacylCoAMAT, C10KetoacylCoAMAT,
    C8AcylCarCYT, C8AcylCarMAT, C8AcylCoAMAT, C8EnoylCoAMAT,
    C8HydroxyacylCoAMAT, C8KetoacylCoAMAT,
    C6AcylCarCYT, C6AcylCarMAT, C6AcylCoAMAT, C6EnoylCoAMAT,
```

```
    C6HydroxyacylCoAMAT, C6KetoacylCoAMAT,
    C4AcylCarCYT, C4AcylCarMAT, C4AcylCoAMAT, C4EnoylCoAMAT,
    C4HydroxyacylCoAMAT, C4AcetoacylCoAMAT,
    AcetylCoAMAT, FADHMAT, NADHMAT};

TableForm[Odes];
TableForm[RateEqs];
TableForm[Odes /. RateEqs /. CoAMATX /. Parm];
TableForm[RateEqs /. Parm];
TableForm[InitialConditions];

tsol = NDSolve[Join[Odes /. RateEqs /. CoAMATX /. Parm, InitialConditions] /.
     x → 26.8 * ⅇ^(-0.18*t), Vars, {t, 0, 30}];
```

Graphs metabolite concentrations

```
<< PlotLegends`

Plot1 = Plot[
  Evaluate[
   {C16AcylCarCYT[t], C14AcylCarCYT[t], C12AcylCarCYT[t], C10AcylCarCYT[t],
     C8AcylCarCYT[t], C6AcylCarCYT[t], C4AcylCarCYT[t]} /. tsol],
  {t, 0, 25}, PlotStyle → {{Red, Thick}, {Green, Thick}, {Blue, Thick},
    {Magenta, Thick}, {Cyan, Thick}, {Orange, Thick}, {Purple, Thick}},
  PlotLegend → {"C16AcylCarCYT", "C14AcylCarCYT", "C12AcylCarCYT",
    "C10AcylCarCYT", "C8AcylCarCYT", "C6AcylCarCYT",
    "C4AcylCarCYT"}, LegendPosition → {1, -0.5},
  PlotRange → All, Frame → True, FrameLabel →
   {"Time (min)", "Concentrations (μM)"},
  BaseStyle → {FontSize → 14, FontFamily → "Helvetica", FontWeight → "Bold"},
  FrameStyle → Thickness[0.005], ImageSize → Scaled[0.5]]

Plot2 = Plot[
  Evaluate[
   {CoAMATt - C16AcylCoAMAT[t] - C16EnoylCoAMAT[t] - C16HydroxyacylCoAMAT[t] -
        C16KetoacylCoAMAT[t] - C14AcylCoAMAT[t] - C14EnoylCoAMAT[t] -
        C14HydroxyacylCoAMAT[t] - C14KetoacylCoAMAT[t] - C12AcylCoAMAT[t] -
        C12EnoylCoAMAT[t] - C12HydroxyacylCoAMAT[t] - C12KetoacylCoAMAT[t] -
        C10AcylCoAMAT[t] - C10EnoylCoAMAT[t] - C10HydroxyacylCoAMAT[t] -
        C10KetoacylCoAMAT[t] - C8AcylCoAMAT[t] - C8EnoylCoAMAT[t] -
        C8HydroxyacylCoAMAT[t] - C8KetoacylCoAMAT[t] - C6AcylCoAMAT[t] -
        C6EnoylCoAMAT[t] - C6HydroxyacylCoAMAT[t] - C6KetoacylCoAMAT[t] -
        C4AcylCoAMAT[t] - C4EnoylCoAMAT[t] - C4HydroxyacylCoAMAT[t] -
        C4AcetoacylCoAMAT[t] - AcetylCoAMAT[t] /. RateEqs /. Parm} /. tsol],
  {t, 0, 25}, PlotStyle → {{Black, Thick}},
  PlotRange → {All, {0, 5000}}, Frame → True,
  FrameLabel → {"Time (min)", "Concentration (μM)"},
  BaseStyle → {FontSize → 14, FontFamily → "Helvetica", FontWeight → "Bold"},
  FrameStyle → Thickness[0.005], ImageSize → Scaled[0.5]]
```

Graphs rates

```
Plot11 = Plot[
  Evaluate[{(vnadhsink + vfadhsink) / 2 * 1000} /. RateEqs /. CoAMATX /. Parm /. tsol],
  {t, 0, 8}, PlotStyle → {{Black, Thick}},
  PlotLegend → {"vnadhsink"}, LegendPosition → {1, -0.5},
  PlotRange → {All, {0, 50}}, Frame → True,
  FrameLabel → {"Time (min)", "v (μmol/min/gProtein)"},
  BaseStyle → {FontSize → 14, FontFamily → "Helvetica", FontWeight → "Bold"},
  FrameStyle → Thickness[0.005], ImageSize → Scaled[0.5]]
```

```
TableForm[
 Table[{(vnadhsink + vfadhsink) / 2 * 1000 /. RateEqs /. CoAMATX /. Parm /. tsol},
  {t, 0, 8.24, 0.01}]]
```

```
TableForm[Table[{C16AcylCarCYT[t] /. tsol}, {t, 0, 24, 0.1}]]
```

```
TableForm[Table[{C14AcylCarCYT[t] /. tsol}, {t, 0, 24, 0.1}]]
```

```
TableForm[Table[{C12AcylCarCYT[t] /. tsol}, {t, 0, 24, 0.1}]]
```

```
TableForm[Table[{C10AcylCarCYT[t] /. tsol}, {t, 0, 24, 0.1}]]
```

```
TableForm[Table[{C8AcylCarCYT[t] /. tsol}, {t, 0, 24, 0.1}]]
```

```
TableForm[Table[{C6AcylCarCYT[t] /. tsol}, {t, 0, 24, 0.1}]]
```

```
TableForm[Table[{C4AcylCarCYT[t] /. tsol}, {t, 0, 24, 0.1}]]
```
