# Supplementary material for: Biochemical Competition Makes Fatty-Acid β-Oxidation Vulnerable to Substrate Overload
Source: PLoS Comput Biol. 2013 Aug 15;9(8):e1003186. doi: 10.1371/journal.pcbi.1003186 (PMC3744394; doi:10.1371/journal.pcbi.1003186)
Supplement: Table S1 — Sensitivity analysis of the standard model. Response coefficients of the concentration of CoASH towards the model parameters p. The response coefficient was approximated by increasing the parameter of interest by 1%. Response coefficients are ranked from the highest to the lowest absolute value (positive coefficients in the left-hand column; negative coefficients in the right-hand column). Response coefficients were calculated for the steady-state standard model with 25 µM palmitoyl CoA as substrate. (PDF) [file pcbi.1003186.s007.pdf]

Table S1: Sensitivity analysis of the standard model

Response coefficients of the concentration of CoASH towards the model parameters *p*. The response coefficient $R_{p_i}^{j} = \frac{d\ln[\text{CoASH}]}{d\ln p_i}$ was approximated by increasing the parameter of interest by 1%. Response coefficients are ranked from the highest to the lowest absolute value (positive coefficients in the left-hand column; negative coefficients in the right-hand column). Response coefficients were calculated for the steady-state standard model with 25µM palmitoyl CoA as substrate.

Parameter	$R_{p_i}^{CoASH}$	Parameter	$R_{p_i}^{CoASH}$
Kmcpt1CarCYT	0.422	sfcpt1C16	-0.534
CoACYT	0.335	Vcpt1	-0.534
NADtMAT	0.323	CarCYT	-0.450
Vmckat	0.302	Kmcpt1CoACYT	-0.345
FADtMAT	0.302	K1nadhsink	-0.335
Keqmschad	0.299	K1fadhsink	-0.271
sfmckatC4	0.238	KmmckatC4AcetoacylCoAMAT	-0.245
Keqcrot	0.225	C16AcylCoACYT	-0.191
Kmcpt1C16AcylCoACYT	0.181	K1acesink	-0.093
KmmckatC4AcylCoAMAT	0.096	KmmckatC6KetoacylCoAMAT	-0.065
Vmcad	0.091	KmscadC4AcylCoAMAT	-0.058
KmmckatAcetylCoAMAT	0.074	KmmcadC6AcylCoAMAT	-0.050
KmmcadC4EnoylCoAMAT	0.066	KmmcadFAD	-0.026
sfmckatC6	0.065	KmmcadC8AcylCoAMAT	-0.023
Vscad	0.059	KmscadFAD	-0.021
sfscadC4	0.059	KmmcadC10AcylCoAMAT	-0.012
KmmckatC6AcylCoAMAT	0.055	KmlcadFAD	-0.011
sfmcadC6	0.051	KmlcadC10AcylCoAMAT	-0.008
Keqscad	0.050	KmlcadC12AcylCoAMAT	-0.007
KmscadC4EnoylCoAMAT	0.045	KmmckatCoAMAT	-0.006
Vlcad	0.038	Keqcpt1	-0.006
sfmcadC8	0.024	Keqcact	-0.003
KmmckatC8AcylCoAMAT	0.020	KmlcadC14AcylCoAMAT	-0.003
KmmcadC6EnoylCoAMAT	0.018	KmmtpC8EnoylCoAMAT	-0.003
KmmckatC10AcylCoAMAT	0.017	Vfcact	-0.003
Keqmcad	0.015	KmvlcadFAD	-0.003
sfmcadC10	0.013	KmvlcadC14AcylCoAMAT	-0.003
sflcadC12	0.012	CarMAT	-0.002
KmscadC6EnoylCoAMAT	0.012	sfcpt2C16	-0.002
sflcadC10	0.011	Vcpt2	-0.002
Vvlcad	0.010	KmmtpNADMAT	-0.002
Vmtp	0.008	KmmtpC14EnoylCoAMAT	-0.002
CoAMATt	0.008	KicactCarCYT	-0.001
KmmckatC12AcylCoAMAT	0.007	KmmtpC10EnoylCoAMAT	-0.001
sflcadC14	0.007	KmmcadC12AcylCoAMAT	-0.001

Parameter	$R_{p_i}^{CoASH}$	Parameter	$R_{p_i}^{CoASH}$
KmscadFADH	0.006	KmvlcadC16AcylCoAMAT	-0.001
sflcadC16	0.006	KmmtpC16EnoylCoAMAT	-0.001
KmmckatC14AcylCoAMAT	0.005	KmmtpC12EnoylCoAMAT	-0.001
sfvlcadC14	0.004	KmmschadNADMAT	-0.001
sfvlcadC12	0.004	Keqcpt2	-0.001
KmmtpC6AcylCoAMAT	0.003	KmmschadC4HydroxyacylCoAMAT	-0.001
sfmtpC8	0.003	KmlcadC8AcylCoAMAT	-0.001
KmcactC16AcylCarCYT	0.003	KmlcadC16AcylCoAMAT	0.000
KmlcadC8EnoylCoAMAT	0.003	KmscadC6AcylCoAMAT	0.000
Kmcpt2C16AcylCarMAT	0.002	Kmcpt2C8AcylCoAMAT	0.000
KmmckatC16AcylCoAMAT	0.002	KmmcadC4AcylCoAMAT	0.000
Vmschad	0.002	Vrcact	0.000
sfmcadC12	0.002	Kmcpt2C10AcylCoAMAT	0.000
sfvlcadC16	0.002	Kmcpt1C16AcylCarCYT	0.000
sfmschadC4	0.002	KmcrotC4EnoylCoAMAT	0.000
sfmtpC14	0.002	KmcrotC6EnoylCoAMAT	0.000
sflcadC8	0.002	KmmtpCoAMAT	0.000
sfmtpC10	0.001	Kmcpt2C12AcylCoAMAT	0.000
sfmtpC16	0.001	Kmcpt2C4AcylCarMAT	0.000
KmlcadC10EnoylCoAMAT	0.001	Kmcpt2C14AcylCoAMAT	0.000
sfmtpC12	0.001	Kmcpt2C6AcylCarMAT	0.000
KmmtpC8AcylCoAMAT	0.001	Kmcpt2CarMAT	0.000
KmlcadC12EnoylCoAMAT	0.001	Kmcpt2C16AcylCoAMAT	0.000
Keqmtp	0.001	Kmcpt2C6AcylCoAMAT	0.000
KmlcadC14EnoylCoAMAT	0.001	KmmckatC8KetoacylCoAMAT	0.000
KmmtpC10AcylCoAMAT	0.001	Kmcpt2C8AcylCarMAT	0.000
KicrotC4AcetoacylCoA	0.001	Kmcpt2C10AcylCarMAT	0.000
KmlcadC16EnoylCoAMAT	0.001	sfcrotC14	0.000
KmmschadNADHMAT	0.001	Kmcpt2C12AcylCarMAT	0.000
sfmcadC4	0.001	Kmcpt2C14AcylCarMAT	0.000
Vcrot	0.001	sfcrotC12	0.000
Keqmckat	0.001	sfcrotC10	0.000
KmmtpNADHMAT	0.001	sfcrotC8	0.000
KmmcadFADH	0.000	Kmcpt2C4AcylCoAMAT	0.000
sfmschadC6	0.000	sfmschadC12	0.000
sfscadC6	0.000	KicactC16AcylCarCYT	0.000
KmmtpC12AcylCoAMAT	0.000	sfmschadC14	0.000
sfcrotC4	0.000		
KmcactC16AcylCarMAT	0.000		
Keqlcad	0.000		
KmmtpC14AcylCoAMAT	0.000		
KmcrotC4HydroxyacylCoAMAT	0.000		
KmvlcadC12EnoylCoAMAT	0.000		
sfcrotC6	0.000		
KmvlcadC14EnoylCoAMAT	0.000		

Parameter	$R_{p_i}^{CoASH}$	Parameter	$R_{p_i}^{CoASH}$
KmvlcadC16EnoylCoAMAT	0.000		
KmmschadC6HydroxyacylCoAMAT	0.000		
KmlcadFADH	0.000		
KmmcadC8EnoylCoAMAT	0.000		
KmvlcadC12AcylCoAMAT	0.000		
KmmtpAcetylCoAMAT	0.000		
KmmtpC16AcylCoAMAT	0.000		
Keqvlcad	0.000		
KmmcadC10EnoylCoAMAT	0.000		
KmmcadC12EnoylCoAMAT	0.000		
KmcrotC6HydroxyacylCoAMAT	0.000		
Kmcpt2CoAMAT	0.000		
KmvlcadFADH	0.000		
sfmckatC8	0.000		
KmmschadC16HydroxyacylCoAMAT	0.000		
KmmschadC14HydroxyacylCoAMAT	0.000		
KmmschadC12HydroxyacylCoAMAT	0.000		
KmmckatC16KetoacylCoAMAT	0.000		
sfmckatC14	0.000		
sfmckatC10	0.000		
KmmschadC4AcetoacylCoAMAT	0.000		
KmmschadC8HydroxyacylCoAMAT	0.000		
sfmckatC12	0.000		
KmmschadC10HydroxyacylCoAMAT	0.000		
KmmschadC6KetoacylCoAMAT	0.000		
KmmckatC12KetoacylCoAMAT	0.000		
KmcrotC14EnoylCoAMAT	0.000		
KmcactCarCYT	0.000		
KmmckatC14KetoacylCoAMAT	0.000		
KmcrotC12EnoylCoAMAT	0.000		
KmcactCarMAT	0.000		
KmcrotC10EnoylCoAMAT	0.000		
KmcrotC8EnoylCoAMAT	0.000		
KmmckatC10KetoacylCoAMAT	0.000		
KmcrotC8HydroxyacylCoAMAT	0.000		
KmcrotC10HydroxyacylCoAMAT	0.000		
KmcrotC12HydroxyacylCoAMAT	0.000		
KmcrotC14HydroxyacylCoAMAT	0.000		
KmcrotC16HydroxyacylCoAMAT	0.000		
sfmschadC8	0.000		
KmmschadC12KetoacylCoAMAT	0.000		
KmmschadC8KetoacylCoAMAT	0.000		
KmmschadC14KetoacylCoAMAT	0.000		
KmmschadC16KetoacylCoAMAT	0.000		
KmmschadC10KetoacylCoAMAT	0.000		

**Parameter**	$R_{p_i}^{CoASH}$	**Parameter**	$R_{p_i}^{CoASH}$
KmcrotC16EnoylCoAMAT	0.000		
sfmschadC10	0.000		
Ksfadhsink	0.000		
Ksnadhsink	0.000		
Ksacesink	0.000		
KmcactC8AcylCarMAT	0.000		
KicactC12AcylCarCYT	0.000		
Kicpt1MalCoACYT	0.000		
ncpt1	0.000		
KmcactC14AcylCarCYT	0.000		
KmcactC12AcylCarCYT	0.000		
KmcactC10AcylCarCYT	0.000		
KmcactC8AcylCarCYT	0.000		
KmcactC6AcylCarCYT	0.000		
KmcactC4AcylCarCYT	0.000		
KmcactC14AcylCarMAT	0.000		
KmcactC12AcylCarMAT	0.000		
KmcactC10AcylCarMAT	0.000		
KmcactC6AcylCarMAT	0.000		
KmcactC4AcylCarMAT	0.000		
KicactC14AcylCarCYT	0.000		
KicactC10AcylCarCYT	0.000		
KicactC8AcylCarCYT	0.000		
KicactC6AcylCarCYT	0.000		
KicactC4AcylCarCYT	0.000		
sfcpt2C14	0.000		
sfcpt2C12	0.000		
sfcpt2C10	0.000		
sfcpt2C8	0.000		
sfcpt2C6	0.000		
sfcpt2C4	0.000		
sfcrotC16	0.000		
sfmschadC16	0.000		
sfmckatC16	0.000		
MalCoACYT	0.000		
VCYT	0.000		
VMAT	0.000		
