# Supplementary material for: Biochemical Competition Makes Fatty-Acid β-Oxidation Vulnerable to Substrate Overload
Source: PLoS Comput Biol. 2013 Aug 15;9(8):e1003186. doi: 10.1371/journal.pcbi.1003186 (PMC3744394; doi:10.1371/journal.pcbi.1003186)
Supplement: Text S1 — Model description. (PDF) [file pcbi.1003186.s008.pdf]

Text S1: Model description

1 Ordinary differential equations

Based on the reaction scheme in Figure 1, a set of ordinary differential equations was constructed. As indicated in the figure, most of the enzymes catalyze multiple reactions, *i.e.* with substrates of different chain length, and many substrates can be converted by different enzymes. For instance v_{cpt1C16} is the rate of conversion of C16 (palmitoyl) CoA by CPT1. In the abbreviations CYT indicates the cytosolic metabolite pool and MAT the metabolite pool in the mitochondrial matrix.

$$\frac{d\mathrm{C16AcylCarCYT}}{dt} = \frac{v_{\mathrm{cpt1C16}} - v_{\mathrm{cactC16}}}{V_{\mathrm{CYT}}} \quad (1)$$

$$\frac{d\mathrm{C16AcylCarMAT}}{dt} = \frac{v_{\mathrm{cactC16}} - v_{\mathrm{cpt2C16}}}{V_{\mathrm{MAT}}} \quad (2)$$

$$\frac{d\mathrm{C16AcylCoAMAT}}{dt} = \frac{v_{\mathrm{cpt2C16}} - v_{\mathrm{vlcadC16}} - v_{\mathrm{lcadC16}}}{V_{\mathrm{MAT}}} \quad (3)$$

$$\frac{d\mathrm{C16EnoylCoAMAT}}{dt} = \frac{v_{\mathrm{vlcadC16}} + v_{\mathrm{lcadC16}} - v_{\mathrm{crotC16}} - v_{\mathrm{mtpC16}}}{V_{\mathrm{MAT}}} \quad (4)$$

$$\frac{d\mathrm{C16HydroxyacylCoAMAT}}{dt} = \frac{v_{\mathrm{crotC16}} - v_{\mathrm{mschadC16}}}{V_{\mathrm{MAT}}} \quad (5)$$

$$\frac{d\mathrm{C16KetoacylCoAMAT}}{dt} = \frac{v_{\mathrm{mschadC16}} - v_{\mathrm{mckatC16}}}{V_{\mathrm{MAT}}} \quad (6)$$

$$\frac{d\mathrm{C14AcylCarCYT}}{dt} = \frac{-v_{\mathrm{cactC14}}}{V_{\mathrm{CYT}}} \quad (7)$$

$$\frac{d\mathrm{C14AcylCarMAT}}{dt} = \frac{v_{\mathrm{cactC14}} - v_{\mathrm{cpt2C14}}}{V_{\mathrm{MAT}}} \quad (8)$$

$$\frac{d\mathrm{C14AcylCoAMAT}}{dt} = \frac{v_{\mathrm{cpt2C14}} + v_{\mathrm{mtpC16}} + v_{\mathrm{mckatC16}} - v_{\mathrm{vlcadC14}} - v_{\mathrm{lcadC14}}}{V_{\mathrm{MAT}}} \quad (9)$$

$$\frac{d\mathrm{C14EnoylCoAMAT}}{dt} = \frac{v_{\mathrm{vlcadC14}} + v_{\mathrm{lcadC14}} - v_{\mathrm{crotC14}} - v_{\mathrm{mtpC14}}}{V_{\mathrm{MAT}}} \quad (10)$$

$$\frac{d\mathrm{C14HydroxyacylCoAMAT}}{dt} = \frac{v_{\mathrm{crotC14}} - v_{\mathrm{mschadC14}}}{V_{\mathrm{MAT}}} \quad (11)$$

$$\frac{d\mathrm{C14KetoacylCoAMAT}}{dt} = \frac{v_{\mathrm{mschadC14}} - v_{\mathrm{mckatC14}}}{V_{\mathrm{MAT}}} \quad (12)$$

$$\frac{d\mathrm{C12AcylCarCYT}}{dt} = \frac{-v_{\mathrm{cactC12}}}{V_{\mathrm{CYT}}} \quad (13)$$

$$\frac{d\mathrm{C12AcylCarMAT}}{dt} = \frac{v_{\mathrm{cactC12}} - v_{\mathrm{cpt2C12}}}{V_{\mathrm{MAT}}} \quad (14)$$

$$\frac{d\mathrm{C12AcylCoAMAT}}{dt} = \frac{v_{\mathrm{cpt2C12}} + v_{\mathrm{mtpC14}} + v_{\mathrm{mckatC14}} - v_{\mathrm{vlcadC12}} - v_{\mathrm{lcadC12}} - v_{\mathrm{mcadC12}}}{V_{\mathrm{MAT}}} \quad (15)$$

$$\frac{d\mathrm{C12EnoylCoAMAT}}{dt} = \frac{v_{\mathrm{vlcadC12}} + v_{\mathrm{lcadC12}} + v_{\mathrm{mcadC12}} - v_{\mathrm{crotC12}} - v_{\mathrm{mtpC12}}}{V_{\mathrm{MAT}}} \quad (16)$$

$$\frac{d\mathrm{C12HydroxyacylCoAMAT}}{dt} = \frac{v_{\mathrm{crotC12}} - v_{\mathrm{mschadC12}}}{V_{\mathrm{MAT}}} \quad (17)$$

$$\frac{d\text{C12KetoacylCoAMAT}}{dt} = \frac{v_{\text{mschadC12}} - v_{\text{mckatC12}}}{V_{\text{MAT}}} \tag{18}$$

$$\frac{d\text{C10AcylCarCYT}}{dt} = \frac{-v_{\text{cactC10}}}{V_{\text{CYT}}} \tag{19}$$

$$\frac{d\text{C10AcylCarMAT}}{dt} = \frac{v_{\text{cactC10}} - v_{\text{cpt2C10}}}{V_{\text{MAT}}} \tag{20}$$

$$\frac{d\text{C10AcylCoAMAT}}{dt} = \frac{v_{\text{cpt2C10}} + v_{\text{mtpC12}} + v_{\text{mckatC12}} - v_{\text{lcadC10}} - v_{\text{mcadC10}}}{V_{\text{MAT}}} \tag{21}$$

$$\frac{d\text{C10EnoylCoAMAT}}{dt} = \frac{v_{\text{lcadC10}} + v_{\text{mcadC10}} - v_{\text{crotC10}} - v_{\text{mtpC10}}}{V_{\text{MAT}}} \tag{22}$$

$$\frac{d\text{C10HydroxyacylCoAMAT}}{dt} = \frac{v_{\text{crotC10}} - v_{\text{mschadC10}}}{V_{\text{MAT}}} \tag{23}$$

$$\frac{d\text{C10KetoacylCoAMAT}}{dt} = \frac{v_{\text{mschadC10}} - v_{\text{mckatC10}}}{V_{\text{MAT}}} \tag{24}$$

$$\frac{d\text{C8AcylCarCYT}}{dt} = \frac{-v_{\text{cactC8}}}{V_{\text{CYT}}} \tag{25}$$

$$\frac{d\text{C8AcylCarMAT}}{dt} = \frac{v_{\text{cactC8}} - v_{\text{cpt2C8}}}{V_{\text{MAT}}} \tag{26}$$

$$\frac{d\text{C8AcylCoAMAT}}{dt} = \frac{v_{\text{cpt2C8}} + v_{\text{mtpC10}} + v_{\text{mckatC10}} - v_{\text{lcadC8}} - v_{\text{mcadC8}}}{V_{\text{MAT}}} \tag{27}$$

$$\frac{d\text{C8EnoylCoAMAT}}{dt} = \frac{v_{\text{lcadC8}} + v_{\text{mcadC8}} - v_{\text{crotC8}} - v_{\text{mtpC8}}}{V_{\text{MAT}}} \tag{28}$$

$$\frac{d\text{C8HydroxyacylCoAMAT}}{dt} = \frac{v_{\text{crotC8}} - v_{\text{mschadC8}}}{V_{\text{MAT}}} \tag{29}$$

$$\frac{d\text{C8KetoacylCoAMAT}}{dt} = \frac{v_{\text{mschadC8}} - v_{\text{mckatC8}}}{V_{\text{MAT}}} \tag{30}$$

$$\frac{d\text{C6AcylCarCYT}}{dt} = \frac{-v_{\text{cactC6}}}{V_{\text{CYT}}} \tag{31}$$

$$\frac{d\text{C6AcylCarMAT}}{dt} = \frac{v_{\text{cactC6}} - v_{\text{cpt2C6}}}{V_{\text{MAT}}} \tag{32}$$

$$\frac{d\text{C6AcylCoAMAT}}{dt} = \frac{v_{\text{cpt2C6}} + v_{\text{mtpC8}} + v_{\text{mckatC8}} - v_{\text{mcadC6}} - v_{\text{scadC6}}}{V_{\text{MAT}}} \tag{33}$$

$$\frac{d\text{C6EnoylCoAMAT}}{dt} = \frac{v_{\text{mcadC6}} + v_{\text{scadC6}} - v_{\text{crotC6}}}{V_{\text{MAT}}} \tag{34}$$

$$\frac{d\text{C6HydroxyacylCoAMAT}}{dt} = \frac{v_{\text{crotC6}} - v_{\text{mschadC6}}}{V_{\text{MAT}}} \tag{35}$$

$$\frac{d\text{C6KetoacylCoAMAT}}{dt} = \frac{v_{\text{mschadC6}} - v_{\text{mckatC6}}}{V_{\text{MAT}}} \tag{36}$$

$$\frac{d\text{C4AcylCarCYT}}{dt} = \frac{-v_{\text{cactC4}}}{V_{\text{CYT}}} \tag{37}$$

$$\frac{d\text{C4AcylCarMAT}}{dt} = \frac{v_{\text{cactC4}} - v_{\text{cpt2C4}}}{V_{\text{MAT}}} \tag{38}$$

$$\frac{d\text{C4AcylCoAMAT}}{dt} = \frac{v_{\text{cpt2C4}} + v_{\text{mckatC6}} - v_{\text{mcadC4}} - v_{\text{scadC4}}}{V_{\text{MAT}}} \tag{39}$$

$$\frac{d\text{C4EnoylCoAMAT}}{dt} = \frac{v_{\text{mcadC4}} + v_{\text{scadC4}} - v_{\text{crotC4}}}{V_{\text{MAT}}} \tag{40}$$

$$\frac{d\text{C4HydroxyacylCoAMAT}}{dt} = \frac{v_{\text{crotC4}} - v_{\text{mschadC4}}}{V_{\text{MAT}}} \tag{41}$$

$$\frac{d\text{C4AcetoacetylCoAMAT}}{dt} = \frac{v_{\text{mschadC4}} - v_{\text{mckatC4}}}{V_{\text{MAT}}} \tag{42}$$

$$\frac{d\mathrm{AcetylCoAMAT}}{d\mathrm{t}} = \frac{\begin{array}{c} v_{\mathrm{mtpC16}} + v_{\mathrm{mckatC16}} + v_{\mathrm{mtpC14}} + v_{\mathrm{mckatC14}} + v_{\mathrm{mtpC12}} + v_{\mathrm{mckatC12}} \\ + v_{\mathrm{mtpC10}} + v_{\mathrm{mckatC10}} + v_{\mathrm{mtpC8}} + v_{\mathrm{mckatC8}} + v_{\mathrm{mckatC6}} + 2 \cdot v_{\mathrm{mckatC4}} - v_{\mathrm{acesink}} \end{array}}{V_{\mathbf{MAT}}} \quad (43)$$

$$\frac{d\mathrm{FADHMAT}}{d\mathrm{t}} = \frac{\begin{array}{c} v_{\mathrm{vlcadC16}} + v_{\mathrm{vlcadC14}} + v_{\mathrm{vlcadC12}} + v_{\mathrm{lcadC16}} + v_{\mathrm{lcadC14}} + v_{\mathrm{lcadC12}} \\ + v_{\mathrm{lcadC10}} + v_{\mathrm{lcadC8}} + v_{\mathrm{mcadC12}} + v_{\mathrm{mcadC10}} + v_{\mathrm{mcadC8}} + v_{\mathrm{mcadC6}} \\ + v_{\mathrm{mcadC4}} + v_{\mathrm{scadC6}} + v_{\mathrm{scadC4}} - v_{\mathrm{fadhsink}} \end{array}}{V_{\mathbf{MAT}}} \quad (44)$$

$$\frac{d\mathrm{NADHMAT}}{d\mathrm{t}} = \frac{\begin{array}{c} v_{\mathrm{mtpC16}} + v_{\mathrm{mtpC14}} + v_{\mathrm{mtpC12}} + v_{\mathrm{mtpC10}} + v_{\mathrm{mtpC8}} + v_{\mathrm{mschadC16}} + v_{\mathrm{mschadC14}} + v_{\mathrm{mschadC12}} \\ + v_{\mathrm{mschadC10}} + v_{\mathrm{mschadC8}} + v_{\mathrm{mschadC6}} + v_{\mathrm{mschadC4}} - v_{\mathrm{nadhsink}} \end{array}}{V_{\mathbf{MAT}}} \quad (45)$$

2 Kinetic rate equations

When an enzyme catalyzes the conversion of multiple substrates, the same rate equation applies, but many of the rate constants are chain-length-specific, as indicated by the subscript n. Most equations are of the reversible Michaelis-Menten type (based on random binding of substrates). The only exception is the rate equations for the transporter CACT. In the model description the rates are given in $\mu mol.min^{-1}.mgProtein^{-1}$, while the rates of change in the differential equations are in $\mu M.min^{-1}$. In the presentation of the results the fluxes were converted to $\mu mol.min^{-1}.gProtein^{-1}$. As in the Modre-Osprian model (1), the rate equations for the consumption of the end products NADH, $FADH_2$ and acetyl CoA were made up such that *i)* the sink reactions do not control the flux; and *ii)* the concentrations of these metabolites equal the constant $K1_{xsink}$. For the computational outcome this is equivalent to fixing the concentrations of NADH, $FADH_2$ and acetyl CoA as external parameters. The advantage of the formulation used here, is that it allows to directly monitor the fluxes of end product removal.

$$v_{\mathrm{cpt1C16}} = \frac{sf_{\mathrm{cpt1C16}} \cdot V_{\mathrm{cpt1}} \cdot \left(\frac{\mathrm{C16AcylCoACYT} \cdot \mathrm{CarCYT}}{Km_{\mathrm{C16AcylCoACYT}} \cdot Km_{\mathrm{CarCYT}}} - \frac{\mathrm{C16AcylCarCYT[t]} \cdot \mathrm{CoACYT}}{Km_{\mathrm{C16AcylCoACYT}} \cdot Km_{\mathrm{CarCYT}} \cdot Keq_{\mathrm{cpt1}}} \right)}{\left(1 + \frac{\mathrm{C16AcylCoACYT}}{Km_{\mathrm{C16AcylCoACYT}}} + \frac{\mathrm{C16AcylCarCYT[t]}}{Km_{\mathrm{C16AcylCarCYT}}} + \left(\frac{\mathrm{MalCoACYT}}{Ki_{\mathrm{MalCoACYT}}} \right)^{n_{\mathrm{cpt1}}} \right) \cdot \left(1 + \frac{\mathrm{CarCYT}}{Km_{\mathrm{CarCYT}}} + \frac{\mathrm{CoACYT}}{Km_{\mathrm{CoACYT}}} \right)} \tag{46}$$

$$v_{\mathrm{cactCn}}(\mathrm{n} \rightarrow 4, 6, 8, 10, 12, 14\ or\ 16) = \frac{Vf_{\mathrm{cact}} \cdot \left(\mathrm{CnAcylCarCYT[t]} \cdot \mathrm{CarMAT} - \frac{\mathrm{CnAcylCarMAT[t]} \cdot \mathrm{CarCYT}}{Keq_{\mathrm{cact}}} \right)}{\begin{array}{c} \mathrm{CnAcylCarCYT[t]} \cdot \mathrm{CarMAT} + Km_{\mathrm{CarMAT}} \cdot \mathrm{CnAcylCarCYT[t]} + Km_{\mathrm{CnAcylCarCYT}} \cdot \mathrm{CarMAT} \cdot \left(1 + \frac{\mathrm{CarCYT}}{Ki_{\mathrm{CatCYT}}} \right) \\ + \frac{Vf_{\mathrm{cact}}}{Vr_{\mathrm{cact}} \cdot Keq_{\mathrm{cact}}} \cdot \left(Km_{\mathrm{CarCYT}} \cdot \mathrm{CnAcylCarMAT[t]} \cdot \left(1 + \frac{\mathrm{CnAcylCarCYT[t]}}{Ki_{\mathrm{CnAcylCarCYT[t]}}} \right) + \mathrm{CarCYT} \cdot \left(Km_{\mathrm{CnAcylCarMAT[t]}} + \mathrm{CncylCarMAT[t]} \right) \right) \end{array}} \tag{47}$$

$$v_{\mathrm{cpt2Cn}}(\mathrm{n} \rightarrow 4, 6, 8, 10, 12, 14\ or\ 16) = \frac{sf_{\mathrm{cpt2Cn}} \cdot V_{\mathrm{cpt2}} \cdot \left(\frac{\mathrm{CnAcylCarMAT[t]} \cdot \mathrm{CoAMAT}}{Km_{\mathrm{CnAcylCarMAT[t]}} \cdot Km_{\mathrm{CoAMAT}}} - \frac{\mathrm{CnAcylCoAMAT[t]} \cdot \mathrm{CarMAT}}{Km_{\mathrm{CnAcylCarMAT[t]}} \cdot Km_{\mathrm{CoAMAT}} \cdot Keq_{\mathrm{cpt2}}} \right)}{\left(1 + \sum_{\mathrm{n} \rightarrow 4,6,8,10,12,14\ and\ 16}^{\mathrm{Cn}} \left(\frac{\mathrm{CnAcylCarMAT[t]}}{Km_{\mathrm{CnAcylCarMAT[t]}}} + \frac{\mathrm{CnAcylCoAMAT[t]}}{Km_{\mathrm{CnAcylCoAMAT[t]}}} \right) \right) \cdot \left(1 + \frac{\mathrm{CoAMAT}}{Km_{\mathrm{CoAMAT}}} + \frac{\mathrm{CarMAT}}{Km_{\mathrm{CarMAT}}} \right)} \tag{48}$$

$$v_{\mathrm{vlcadCn}}(\mathrm{n} \rightarrow 12, 14\ or\ 16) = \frac{sf_{\mathrm{vlcadCn}} \cdot V_{\mathrm{vlcad}} \cdot \left(\frac{\mathrm{CnAcylCoAMAT[t]} \cdot (\mathrm{FADtMAT\text{-}FADHMAT[t]})}{Km_{\mathrm{CnAcylCoAMAT[t]}} \cdot Km_{\mathrm{FADMAT}}} - \frac{\mathrm{CnEnoylCoAMAT[t]} \cdot \mathrm{FADHMAT[t]}}{Km_{\mathrm{CnAcylCoAMAT[t]}} \cdot Km_{\mathrm{FADMAT}} \cdot Keq_{\mathrm{vlcad}}} \right)}{\left(1 + \sum_{\mathrm{n} \rightarrow 12,14\ and\ 16)}^{\mathrm{Cn}} \left(\frac{\mathrm{CnAcylCoAMAT[t]}}{Km_{\mathrm{CnAcylCoAMAT[t]}}} + \frac{\mathrm{CnEnoylCoAMAT[t]}}{Km_{\mathrm{CnEnoylCoAMAT[t]}}} \right) \right) \cdot \left(1 + \frac{\mathrm{FADtMAT\text{-}FADHMAT[t]}}{Km_{\mathrm{FADMAT}}} + \frac{\mathrm{FADHMAT[t]}}{Km_{\mathrm{FADHMAT}}} \right)} \tag{49}$$

$$v_{\mathrm{lcadCn}}(\mathrm{n}\rightarrow 8,10,12,14\ or\ 16)=\frac{sf_{\mathrm{lcadCn}}\cdot V_{\mathrm{lcad}}\cdot\left(\frac{\mathrm{CnAcylCoAMAT[t]}\cdot(\mathrm{FADtMAT\text{-}FADHMAT[t]})}{Km_{\mathrm{CnAcylCoAMAT[t]}}\cdot Km_{\mathrm{FADMAT}}}-\frac{\mathrm{CnEnoylCoAMAT[t]}\cdot\mathrm{FADHMAT[t]}}{Km_{\mathrm{CnAcylCoAMAT[t]}}\cdot Km_{\mathrm{FADMAT}}\cdot Keq_{\mathrm{lcad}}}\right)}{\left(1+\sum_{\mathrm{n}\rightarrow 8,10,12,14\ and\ 16)}^{\mathrm{Cn}}\left(\frac{\mathrm{CnAcylCoAMAT[t]}}{Km_{\mathrm{CnAcylCoAMAT[t]}}}+\frac{\mathrm{CnEnoylCoAMAT[t]}}{Km_{\mathrm{CnEnoylCoAMAT[t]}}}\right)\right)\cdot\left(1+\frac{\mathrm{FADtMAT\text{-}FADHMAT[t]}}{Km_{\mathrm{FADMAT}}}+\frac{\mathrm{FADHMAT[t]}}{Km_{\mathrm{FADHMAT}}}\right)} \tag{50}$$

$$v_{\mathrm{mcadCn}}(\mathrm{n}\rightarrow 4,6,8,10\ or\ 12)=\frac{sf_{\mathrm{mcadCn}}\cdot V_{\mathrm{mcad}}\cdot\left(\frac{\mathrm{CnAcylCoAMAT[t]}\cdot(\mathrm{FADtMAT\text{-}FADHMAT[t]})}{Km_{\mathrm{CnAcylCoAMAT[t]}}\cdot Km_{\mathrm{FADMAT}}}-\frac{\mathrm{CnEnoylCoAMAT[t]}\cdot\mathrm{FADHMAT[t]}}{Km_{\mathrm{CnAcylCoAMAT[t]}}\cdot Km_{\mathrm{FADMAT}}\cdot Keq_{\mathrm{mcad}}}\right)}{\left(1+\sum_{\mathrm{n}\rightarrow 4,6,8,10\ and\ 12}^{\mathrm{Cn}}\left(\frac{\mathrm{CnAcylCoAMAT[t]}}{Km_{\mathrm{CnAcylCoAMAT[t]}}}+\frac{\mathrm{CnEnoylCoAMAT[t]}}{Km_{\mathrm{CnEnoylCoAMAT[t]}}}\right)\right)\cdot\left(1+\frac{\mathrm{FADtMAT\text{-}FADHMAT[t]}}{Km_{\mathrm{FADMAT}}}+\frac{\mathrm{FADHMAT[t]}}{Km_{\mathrm{FADHMAT}}}\right)} \tag{51}$$

$$v_{\mathrm{scadCn}}(\mathrm{n}\rightarrow 4\ or\ 6)=\frac{sf_{\mathrm{scadCn}}\cdot V_{\mathrm{scad}}\cdot\left(\frac{\mathrm{CnAcylCoAMAT[t]}\cdot(\mathrm{FADtMAT\text{-}FADHMAT[t]})}{Km_{\mathrm{C4\text{-}C6AcylCoAMAT[t]}}\cdot Km_{\mathrm{FADMAT}}}-\frac{\mathrm{CnEnoylCoAMAT[t]}\cdot\mathrm{FADHMAT[t]}}{Km_{\mathrm{CnAcylCoAMAT[t]}}\cdot Km_{\mathrm{FADMAT}}\cdot Keq_{\mathrm{scad}}}\right)}{\left(1+\sum_{\mathrm{n}\rightarrow 4\ and\ 6}^{\mathrm{Cn}}\left(\frac{\mathrm{CnAcylCoAMAT[t]}}{Km_{\mathrm{CnAcylCoAMAT[t]}}}+\frac{\mathrm{CnEnoylCoAMAT[t]}}{Km_{\mathrm{CnEnoylCoAMAT[t]}}}\right)\right)\cdot\left(1+\frac{\mathrm{FADtMAT\text{-}FADHMAT[t]}}{Km_{\mathrm{FADMAT}}}+\frac{\mathrm{FADHMAT[t]}}{Km_{\mathrm{FADHMAT}}}\right)} \tag{52}$$

$$v_{\mathrm{crotCn}}(\mathrm{n}\rightarrow 4,6,8,10,12,14\ or\ 16)=\frac{sf_{\mathrm{crotCn}}\cdot V_{\mathrm{crot}}\cdot\left(\frac{\mathrm{CnEnoylCoAMAT[t]}}{Km_{\mathrm{CnEnoylCoAMAT[t]}}}-\frac{\mathrm{CnHydroxyacylCoAMAT[t]}}{Km_{\mathrm{CnEnoylCoAMAT[t]}}\cdot Keq_{\mathrm{crot}}}\right)}{1+\sum_{\mathrm{n}\rightarrow 4,6,8,10,12,14\ and\ 16}^{\mathrm{Cn}}\left(\frac{\mathrm{CnEnoylCoAMAT[t]}}{Km_{\mathrm{CnEnoylCoAMAT[t]}}}+\frac{\mathrm{CnHydroxyacylCoAMAT[t]}}{Km_{\mathrm{CnHydroxyacylCoAMAT[t]}}}\right)+\frac{\mathrm{AcetoacetylCoAMAT[t]}}{Ki_{\mathrm{AcetoacetylCoAMAT}}}} \tag{53}$$

$$v_{\mathrm{mschadCn}}(\mathrm{n}\rightarrow 4,6,8,10,12,14\ or\ 16)=\frac{sf_{\mathrm{mschadCn}}\cdot V_{\mathrm{mschad}}\cdot\left(\frac{\mathrm{CnHydroxyacylCoAMAT[t]}\cdot(\mathrm{NADtMAT\text{-}NADHMAT[t]})}{Km_{\mathrm{CnHydroxyacylCoAMAT[t]}}\cdot Km_{\mathrm{NADMAT}}}-\frac{\mathrm{CnketoacylCoAMAT[t]}\cdot\mathrm{NADHMAT[t]}}{Km_{\mathrm{CnHydroxyacylCoAMAT[t]}}\cdot Km_{\mathrm{NADMAT}}\cdot Keq_{\mathrm{mschad}}}\right)}{\left(1+\sum_{\mathrm{n}\rightarrow 4,6,8,10,12,14\ and\ 16}^{\mathrm{Cn}}\left(\frac{\mathrm{CnHydroxyacylCoAMAT[t]}}{Km_{\mathrm{CnHydroxyacylCoAMAT[t]}}}+\frac{\mathrm{CnKetoacylCoAMAT[t]}}{Km_{\mathrm{CnKetoacylCoAMAT[t]}}}\right)\right)\cdot\left(1+\frac{\mathrm{NADtMAT\text{-}NADHMAT[t]}}{Km_{\mathrm{NADMAT}}}+\frac{\mathrm{NADHMAT[t]}}{Km_{\mathrm{NADHMAT}}}\right)} \tag{54}$$

$$v_{\mathrm{mckatCn}}(\mathrm{n}\rightarrow 4,6,8,10,12,14\ or\ 16)=\frac{sf_{\mathrm{mckatCn}}\cdot V_{\mathrm{mckat}}\cdot\left(\frac{\mathrm{CnKetoacylCoAMAT[t]}\cdot\mathrm{CoAMAT}}{Km_{\mathrm{CnKetoacylCoAMAT[t]}}\cdot Km_{\mathrm{CoAMAT}}}-\frac{\mathrm{Cn\text{-}2AcylCoAMAT[t]}\cdot\mathrm{AcetylCoAMAT[t]}}{Km_{\mathrm{CnKetoacylCoAMAT[t]}}\cdot Km_{\mathrm{CoAMAT}}\cdot Keq_{\mathrm{mckat}}}\right)}{\left(1+\sum_{\mathrm{n}\rightarrow 4,6,8,10,12,14\ and\ 16}^{\mathrm{Cn}}\left(\frac{\mathrm{CnKetoacylCoAMAT[t]}}{Km_{\mathrm{CnKetoacylCoAMAT[t]}}}+\frac{\mathrm{CnAcylCoAMAT[t]}}{Km_{\mathrm{CnAcylCoAMAT[t]}}}\right)+\frac{\mathrm{AcetylCoAMAT[t]}}{Km_{\mathrm{AcetylCoAMAT[t]}}}\right)\cdot\left(1+\frac{\mathrm{CoAMAT}}{Km_{\mathrm{CoAMAT}}}+\frac{\mathrm{AcetylCoAMAT[t]}}{Km_{\mathrm{AcetylCoAMAT[t]}}}\right)} \tag{55}$$

$$v_{\mathrm{mtpCn}}(\mathrm{n}\rightarrow 8,10,12,14\ or\ 16)=\frac{sf_{\mathrm{mtpCn}}\cdot V_{\mathrm{mtp}}\cdot\left(\frac{\mathrm{CnEnoylCoAMAT[t]}\cdot(\mathrm{NADtMAT\text{-}NADHMAT[t]})\cdot\mathrm{CoAMAT}}{Km_{\mathrm{CnEnoylCoAMAT[t]}}\cdot Km_{\mathrm{NADMAT}}\cdot Km_{\mathrm{CoAMAT}}}-\frac{\mathrm{Cn\text{-}2AcylCoAMAT[t]}\cdot\mathrm{NADHMAT[t]}\cdot\mathrm{AcetylCoAMAT[t]}}{Km_{\mathrm{CnEnoylCoAMAT[t]}}\cdot Km_{\mathrm{NADMAT}}\cdot Km_{\mathrm{CoAMAT}}\cdot Keq_{\mathrm{mtp}}}\right)}{\left(1+\sum_{\mathrm{n}\rightarrow 8,10,12,14\ and\ 16}^{\mathrm{Cn}}\left(\frac{\mathrm{CnEnoylCoAMAT[t]}}{Km_{\mathrm{CnEnoylCoAMAT[t]}}}+\frac{\mathrm{CnAcylCoAMAT[t]}}{Km_{\mathrm{CnAcylCoAMAT[t]}}}\right)+\frac{\mathrm{C6AcylCoAMAT[t]}}{Km_{\mathrm{C6AcylCoAMAT[t]}}}+\frac{\mathrm{AcetoacetylCoAMAT[t]}}{Ki_{\mathrm{AcetoacetylCoAMAT}}}\right)\cdot\left(1+\frac{\mathrm{NADtMAT\text{-}NADHMAT[t]}}{Km_{\mathrm{NADMAT}}}+\frac{\mathrm{NADHMAT[t]}}{Km_{\mathrm{NADHMAT}}}\right)\cdot\left(1+\frac{\mathrm{CoAMAT}}{Km_{\mathrm{CoAMAT}}}+\frac{\mathrm{AcetylCoAMAT[t]}}{Km_{\mathrm{AcetylCoAMAT[t]}}}\right)} \tag{56}$$

$$v_{\mathrm{acesink}}=Ks_{\mathrm{acesink}}\cdot(\mathrm{AcetylCoAMAT[t]}-K1_{\mathrm{acesink}}) \tag{57}$$

$$v_{\mathrm{fadhsink}}=Ks_{\mathrm{fadhsink}}\cdot(\mathrm{FADHMAT[t]}-K1_{\mathrm{fadhsink}}) \tag{58}$$

$$v_{\mathrm{nadhsink}} = Ks_{\mathrm{nadhsink}} \cdot (\mathrm{NADHMAT[t]} - K1_{\mathrm{nadhsink}}) \tag{59}$$

$$\mathrm{CoAMAT} = \mathrm{CoAMATt} - \sum_{\mathrm{n} \to 8,10,12,14\ and\ 16}^{\mathrm{Cn}} (\mathrm{CnAcylCoAMAT[t]} + \mathrm{CnEnoylCoAMAT[t]} + \mathrm{CnHydroxyacylCoAMAT[t]} + \mathrm{CnKetoacylCoAMAT[t]}) - \mathrm{AcetylCoAMAT[t]} \tag{60}$$

3 Simulations for model validation

The parameters in Table 1 below were used for steady-state calculations, unless parameter variations are indicated in the Figure. These steady-state calculations represent the functioning of mitochondria in the cell. To produce Figure 2 (the *in vitro* experiment with isolated mitochondria), the computer model was slightly adapted to match the conditions used in this experiment. In the experiment the supplied palmitoyl-CoA or palmitoyl-carnitine substrate decreased with time and this time course was imposed on the model, instead of the constant concentration above, which was used for steady state calculations. The substrate consumption dynamics was fitted to the concentration of palmitoyl carnitine over time, which resulted in the following equation:

$$[substrate] = 26.8 \cdot e^{-0.18 \cdot t} \quad (61)$$

Here the substrate concentration is in µM and time *t* in minutes. As we could not measure the time course for palmitoyl CoA, we used the same time course for palmitoyl CoA when it was given as a substrate. In the latter case palmitoyl carnitine was a free variable, predicted by the model and validated independently in the experiment.

The concentrations of CarCYT was set to 400 µM, which was the average value measured over time. For VCYT we took 10^{-2} $L.mgProtein^{-1}$, which here represents the extramitochondrial volume in the reaction vessel rather than the cytosolic volume. The remaining parameters were not changed. The initial concentrations of the acyl carnitines were set to the measured concentrations at time point 0.

Table 1: Kinetic parameters

Parameter	Value		Reference
CPT1			
$sf_{cpt1C16}$	1		
V_{cpt1}	0.012	µmol.min^{-1}.mgProtein^{-1}	Fitted to experimental data
$Km_{cpt1C16AcylCoACYT}$	13.8	µM	(2)
$Km_{cpt1CarCYT}$	250	µM	(2)
$Km_{cpt1C16AcylCarCYT}$	136	µM	(2)
$Km_{cpt1CoACYT}$	40.7	µM	(2)
$Ki_{cpt1MalCoACYT}$	9.1	µM	(3)
Keq_{cpt1}	0.45		(4)
n_{cpt1}	2.4799		Estimated based on data of (5)
CACT			
Vf_{cact}	0.42	µmol.min^{-1}.mgProtein^{-1}	
Vr_{cact}	0.42	µmol.min^{-1}.mgProtein^{-1}	
$Km_{C16AcylCarCYT}$	15	µM	(2)
Km_{CarMAT}	130	µM	(2)
$Km_{C16AcylCarMAT}$	15	µM	(2)
Km_{CarCYT}	130	µM	(2)
$Ki_{C16AcylCarCYT}$	56	µM	(2)
Ki_{CarCYT}	200	µM	(2)
Keq_{cact}	1		Based on passive transport
CPT2			
$sf_{cpt2C16}$	0.85		(6)
$sf_{cpt2C14}$	1		(6)
$sf_{cpt2C12}$	0.95		(6)
$sf_{cpt2C10}$	0.95		(6)
sf_{cpt2C8}	0.35		(6)
sf_{cpt2C6}	0.15		(6)
sf_{cpt2C4}	0.01		(6)
V_{cpt2}	0.391	µmol.min^{-1}.mgProtein^{-1}	(6)
$Km_{CnAcylCarMAT}$	51	µM	(2)
Km_{CoAMAT}	30	µM	(2)
$Km_{CnAcylCoAMAT}$	38	µM	(2)
Km_{CarMAT}	350	µM	(2)
Keq_{cpt2}	2.22		(4)
VLCAD			
$sf_{vlcadC16}$	1		(7)
$sf_{vlcadC14}$	0.42		(7)
$sf_{vlcadC12}$	0.11		(7)
V_{vlcad}	0.008	µmol.min^{-1}.mgProtein^{-1}	Fitted to experimental data
$Km_{C16AcylCoAMAT}$	6.5	µM	(7)
$Km_{C14AcylCoAMAT}$	4	µM	(7)
$Km_{C12AcylCoAMAT}$	2.7	µM	(7)
Km_{FAD}	0.12	µM	(1)
$Km_{C16EnoylCoAMAT}$	1.08	µM	(1)
$Km_{C14EnoylCoAMAT}$	1.08	µM	(1)
$Km_{C12EnoylCoAMAT}$	1.08	µM	(1)
Km_{FADH}	24.2	µM	(1)
Keq_{vlcad}	6		(2)

Parameter	Value		Reference
LCAD			
$sf_{lcadC16}$	0.9		(8)
$sf_{lcadC14}$	1		(8)
$sf_{lcadC12}$	0.9		(8)
$sf_{lcadC10}$	0.75		(8)
sf_{lcadC8}	0.4		(8)
V_{lcad}	0.01	$\mu mol.min^{-1}.mgProtein^{-1}$	Fitted to experimental data
$Km_{C16AcylCoAMAT}$	2.5	µM	(8)
$Km_{C14AcylCoAMAT}$	7.4	µM	(8)
$Km_{C12AcylCoAMAT}$	9	µM	(8)
$Km_{C10AcylCoAMAT}$	24.3	µM	(8)
$Km_{C8AcylCoAMAT}$	123	µM	(8)
Km_{FAD}	0.12	µM	(1)
$Km_{C16EnoylCoAMAT}$	1.08	µM	(1)
$Km_{C14EnoylCoAMAT}$	1.08	µM	(1)
$Km_{C12EnoylCoAMAT}$	1.08	µM	(1)
$Km_{C10EnoylCoAMAT}$	1.08	µM	(1)
$Km_{C8EnoylCoAMAT}$	1.08	µM	(1)
Km_{FADH}	24.2	µM	(1)
Keq_{lcad}	6		(2)
MCAD			
$sf_{mcadC12}$	0.38		(8)
$sf_{mcadC10}$	0.8		(8)
sf_{mcadC8}	0.87		(8)
sf_{mcadC6}	1		(8)
sf_{mcadC4}	0.12		(8)
V_{mcad}	0.081	$\mu mol.min^{-1}.mgProtein^{-1}$	(8)
$Km_{C12AcylCoAMAT}$	5.7	µM	(8)
$Km_{C10AcylCoAMAT}$	5.4	µM	(8)
$Km_{C8AcylCoAMAT}$	4	µM	(8)
$Km_{C6AcylCoAMAT}$	9.4	µM	(8)
$Km_{C4AcylCoAMAT}$	135	µM	(8)
Km_{FAD}	0.12	µM	(1)
$Km_{C12EnoylCoAMAT}$	1.08	µM	(1)
$Km_{C10EnoylCoAMAT}$	1.08	µM	(1)
$Km_{C8EnoylCoAMAT}$	1.08	µM	(1)
$Km_{C6EnoylCoAMAT}$	1.08	µM	(1)
$Km_{C4EnoylCoAMAT}$	1.08	µM	(1)
Km_{FADH}	24.2	µM	(1)
Keq_{mcad}	6		(2)
SCAD			
sf_{scadC6}	0.3		(8)
sf_{scadC4}	1		(8)
V_{scad}	0.081	$\mu mol.min^{-1}.mgProtein^{-1}$	(8)
$Km_{C6AcylCoAMAT}$	285	µM	(8)
$Km_{C4AcylCoAMAT}$	10.7	µM	(8)
Km_{FAD}	0.12	µM	(1)
$Km_{C6EnoylCoAMAT}$	1.08	µM	(1)
$Km_{C4EnoylCoAMAT}$	1.08	µM	(1)
Km_{FADH}	24.2	µM	(1)
Keq_{scad}	6		(2)

Parameter	Value		Reference
CROT			
$sf_{crotC16}$	0.13		(9)
$sf_{crotC14}$	0.2		(9)
$sf_{crotC12}$	0.25		(9)
$sf_{crotC10}$	0.33		(9)
sf_{crotC8}	0.58		(9)
sf_{crotC6}	0.8		(9)
sf_{crotC4}	1		(9)
V_{crot}	3.6	µmol.min^{-1}.mgProtein^{-1}	(9)
$Km_{C16EnoylCoAMAT}$	150	µM	(9)
$Km_{C14EnoylCoAMAT}$	100	µM	(9)
$Km_{C12EnoylCoAMAT}$	25	µM	(9)
$Km_{C10EnoylCoAMAT}$	25	µM	(9)
$Km_{C8EnoylCoAMAT}$	25	µM	(9)
$Km_{C6EnoylCoAMAT}$	25	µM	(9)
$Km_{C4EnoylCoAMAT}$	40	µM	(9)
$Km_{C16HydroxyacylCoAMAT}$	45	µM	(2)
$Km_{C14HydroxyacylCoAMAT}$	45	µM	(2)
$Km_{C12HydroxyacylCoAMAT}$	45	µM	(2)
$Km_{C10HydroxyacylCoAMAT}$	45	µM	(2)
$Km_{C8HydroxyacylCoAMAT}$	45	µM	(2)
$Km_{C6HydroxyacylCoAMAT}$	45	µM	(2)
$Km_{C4HydroxyacylCoAMAT}$	45	µM	(2)
$Ki_{acetoacetylCoAMAT}$	1.6	µM	(10)
Keq_{crot}	3.13		(2)
M/SCHAD			
$sf_{mschadC16}$	0.6		(11)
$sf_{mschadC14}$	0.5		Estimated based on (11)
$sf_{mschadC12}$	0.43		(11)
$sf_{mschadC10}$	0.64		(11)
$sf_{mschadC8}$	0.89		(11)
$sf_{mschadC6}$	1		(11)
$sf_{mschadC4}$	0.67		(11)
V_{mschad}	1	µmol.min^{-1}.mgProtein^{-1}	(12)
$Km_{C16HydroxyacylCoAMAT}$	1.5	µM	(13)
$Km_{C14HydroxyacylCoAMAT}$	1.8	µM	(13)
$\mathrm{Km}_{C12HydroxyacylCoAMAT}$	3.7	µM	(13)
$Km_{C10HydroxyacylCoAMAT}$	8.8	µM	(11)
$Km_{C8HydroxyacylCoAMAT}$	16.3	µM	(11)
$Km_{C6HydroxyacylCoAMAT}$	28.6	µM	(11)
$Km_{C4HydroxyacylCoAMAT}$	69.9	µM	(11)
Km_{NADMAT}	58.5	µM	(11)
$Km_{C16KetoacylCoAMAT}$	1.4	µM	(13)
$Km_{C14KetoacylCoAMAT}$	1.4	µM	(13)
$Km_{C12KetoacylCoAMAT}$	1.6	µM	(13)
$Km_{C10KetoacylCoAMAT}$	2.3	µM	(13)
$Km_{C8KetoacylCoAMAT}$	4.1	µM	(13)
$Km_{C6KetoacylCoAMAT}$	5.8	µM	(13)
$Km_{C4AcetoacylCoAMAT}$	16.9	µM	(11)
$Km_{NADHMAT}$	5.4	µM	(11)
Keq_{mschad}	$2.17 \cdot 10^{-4}$		(2)

Parameter	Value		Reference
MCKAT			
sf_{mckatC16}	0		(11)
sf_{mckatC14}	0.2		Estimated based on (11)
sf_{mckatC12}	0.38		(11)
sf_{mckatC10}	0.65		(11)
sf_{mckatC8}	0.81		(11)
sf_{mckatC6}	1		(11)
sf_{mckatC4}	0.49		(11)
V_{mckat}	0.377	$\mu mol.min^{-1}.mgProtein^{-1}$	(14)
$Km_{\text{C16KetoacylCoAMAT}}$	1.1	µM	(15)
$Km_{\text{C14KetoacylCoAMAT}}$	1.2	µM	Estimated based on (15)
$Km_{\text{C12KetoacylCoAMAT}}$	1.3	µM	(15)
$Km_{\text{C10KetoacylCoAMAT}}$	2.1	µM	(15)
$Km_{\text{C8KetoacylCoAMAT}}$	3.2	µM	(15)
$Km_{\text{C6KetoacylCoAMAT}}$	6.7	µM	(15)
$Km_{\text{C4AcetoacylCoAMAT}}$	12.4	µM	(15)
Km_{CoAMAT}	26.6	µM	Estimated based on (15)
$Km_{\text{C16AcylCoAMAT}}$	13.83	µM	(2)
$Km_{\text{C14AcylCoAMAT}}$	13.83	µM	(2)
$Km_{\text{C12AcylCoAMAT}}$	13.83	µM	(2)
$Km_{\text{C10AcylCoAMAT}}$	13.83	µM	(2)
$Km_{\text{C8AcylCoAMAT}}$	13.83	µM	(2)
$Km_{\text{C6AcylCoAMAT}}$	13.83	µM	(2)
$Km_{\text{C4AcylCoAMAT}}$	13.83	µM	(2)
$Km_{\text{AcetylCoAMAT}}$	30	µM	(2)
Keq_{mckat}	1051		(2)
MTP			
sf_{mtpC16}	1		(11)
sf_{mtpC14}	0.9		(11)
sf_{mtpC12}	0.81		(11)
sf_{mtpC10}	0.73		(11)
sf_{mtpC8}	0.34		(11)
V_{mtp}	2.84	$\mu mol.min^{-1}.mgProtein^{-1}$	(11)
$Km_{\text{C16EnoylCoAMAT}}$	25	µM	(9)
$Km_{\text{C14EnoylCoAMAT}}$	25	µM	(9)
$Km_{\text{C12EnoylCoAMAT}}$	25	µM	(9)
$Km_{\text{C10EnoylCoAMAT}}$	25	µM	(9)
$Km_{\text{C8EnoylCoAMAT}}$	25	µM	(9)
Km_{NADMAT}	60	µM	(11)
Km_{CoAMAT}	30	µM	(15)
$Km_{\text{C16AcylCoAMAT}}$	13.83	µM	(2)
$Km_{\text{C14AcylCoAMAT}}$	13.83	µM	(2)
$Km_{\text{C12AcylCoAMAT}}$	13.83	µM	(2)
$Km_{\text{C10AcylCoAMAT}}$	13.83	µM	(2)
$Km_{\text{C8AcylCoAMAT}}$	13.83	µM	(2)
$Km_{\text{C6AcylCoAMAT}}$	13.83	µM	(2)
Km_{NADHMAT}	50	µM	(11)
$Km_{\text{AcetylCoAMAT}}$	30	µM	(2)
Keq_{mtp}	0.71		Calculated by multiplying Keq_{crot}, Keq_{mschad} and Keq_{mckat}

Parameter	Value		Reference
ACESINK			
$Ks_{acesink}$	6000000	µmol.min^{-1}.mgProtein^{-1}	(1)
$K1_{acesink}$	70	µM	(5)
FADHSINK			
$Ks_{fadhsink}$	6000000	µmol.min^{-1}.mgProtein^{-1}	(1)
$K1_{fadhsink}$	0.46	µM	(1)
NADHSINK			
$Ks_{nadhsink}$	6000000	µmol.min^{-1}.mgProtein^{-1}	(1)
$K1_{nadhsink}$	16	µM	
Total concentrations of the conserved moieties			
FADtMAT	0.77	µM	(1)
NADtMAT	250	µM	
CoAMATt	5000	µM	(16)
Fixed concentrations of metabolites			
CarCYT	200	µM	(1)
CoACYT	140	µM	(16)
CarMAT	950	µM	(1)
Volumes of various compartments			
VCYT	$2.2x10^{-6}$	L.mgProtein^{-1}	(17)
VMAT	$1.8x10^{-6}$	L.mgProtein^{-1}	(18)

Glossary

$v_{enzymeCn}$	Rate for a particular enzyme and carbon-chain length. For instance, $v_{cpt1C16}$ is the rate at which the substrate with 16 C-atoms is converted by CPT1
$Sf_{enzymeCn}$	Specificity factor that determines the enzyme activity for the substrate with a specific chain length as a percentage of the V_{max}. The multiplication of this factor with V_{max} will give the maximum enzyme rate for the substrate with *n* C-atoms.
V_{enzyme}	The V_{max} of a particular enzyme.
$Km_{Cnmetabolite}$	The affinity constant (*Km*) of an enzyme for the metabolite with a specific chain length, *e.g.* $Km_{\mathrm{C16AcylCarCYT}}$ is the affinity constant of an enzyme for the acyl carnitine in the cytosol with 16 C-atoms.
Keq_{enzyme}	The equilibrium constant (*Keq*) for a particular enzyme reaction.
**(Cn)MetaboliteMAT[t]**	Concentration of the metabolite in the mitochondrial matrix cytosol. When it starts with Cn, this denotes the chain length of the metabolite. The t in between brackets at the end depicts that the metabolite is a time-dependent variable.
**(Cn)MetaboliteCYT[t]**	Concentration of the metabolite in the cytosol. When it starts with Cn, this denotes the chain length of the metabolite. The t in between brackets at the end depicts that the metabolite is a time-dependent variable.
$Ki_{Metabolite}$	Inhibition constant of an enzyme with respect to the metabolite. If the metabolite starts with Cn, this denotes the chain length of the metabolite.
n_{cpt1}	Hill coefficient of for the cooperative inhibition of CPT1 by malonyl-CoA.
FADtMAT	Total concentration of oxidized and reduced FAD in the mitochondrial matrix.
NADtMAT	Total concentration of oxidized and reduced NAD in the mitochondrial matrix.
CoAMATt	Total concentration of all CoA-containing species in the mitochondrial matrix.
Car	Carnitine.
CPT1	Carnitine-palmitoyl transferase 1.
CACT	Carnitine-acyl-carnitine translocase.
CPT2	Carnitine-palmitoyl transferase 2.
SCAD	Short-chain acyl-CoA dehydrogenase.
MCAD	Medium-chain acyl-CoA dehydrogenase.
LCAD	Long-chain acyl-CoA dehydrogenase.
VLCAD	Very-long-chain acyl-CoA dehydrogenase.
CROT	Crotonase.
M/SCHAD	Medium/short-chain hydroxyacyl-CoA dehydrogenase.
MCKAT	Medium-chain ketoacyl-CoA thiolase.
MTP	Mitochondrial trifunctional protein.
**VCYT**	Volume of the cytosol.
**VMAT**	Volume of the mitochondrial matrix.
v_{xsink}	Rate of the sink reaction of metabolite x (x is either acetyl-CoA, NADH or $FADH_2$).
Ks_{xsink}	Rate constant of the sink reaction of metabolite x (x is either acetyl-CoA, NADH or $FADH_2$).
$K1_{xsink}$	Constant in the sink reactions that determines the concentration of x (x is either acetyl-CoA, NADH or $FADH_2$).

References

1. Modre-Osprian R, *et al* (2009) Dynamic simulations on the mitochondrial fatty acid beta-oxidation network. *BMC Syst Biol* 3: 2.

2. Kohn MC & Garfinkel D (1983) Computer simulation of metabolism in palmitate-perfused rat heart. I. palmitate oxidation. *Ann Biomed Eng* 11: 361-384.

3. Fraser F, Padovese R & Zammit VA (2001) Distinct kinetics of carnitine palmitoyltransferase i in contact sites and outer membranes of rat liver mitochondria. *The Journal of Biological Chemistry* 276: 20182-20185.

4. Norum KR (1964) Palmityl-CoA:Carnitine palmityltransferase. purification from calf-liver mitochondria and some properties of the enzyme. *Biochimica Et Biophysica Acta* 89: 95-108.

5. van Vlies N, Ruiter JPN, Doolaard M, Wanders RJA & Vaz FM (2007) An improved enzyme assay for carnitine palmitoyl transferase I in fibroblasts using tandem mass spectrometry. *Molecular Genetics and Metabolism* 90: 24-29.

6. Miyazawa S, Ozasa H, Osumi T & Hashimoto T (1983) Purification and properties of carnitine octanoyltransferase and carnitine palmitoyltransferase from rat liver. *Journal of Biochemistry* 94: 529-542.

7. Izai K, Uchida Y, Orii T, Yamamoto S & Hashimoto T (1992) Novel fatty acid beta-oxidation enzymes in rat liver mitochondria. I. purification and properties of very-long-chain acyl-coenzyme A dehydrogenase. *The Journal of Biological Chemistry* 267: 1027-1033.

8. Ikeda Y, Okamura-Ikeda K & Tanaka K (1985) Purification and characterization of short-chain, medium-chain, and long-chain acyl-CoA dehydrogenases from rat liver mitochondria. isolation of the holo- and apoenzymes and conversion of the apoenzyme to the holoenzyme. *J Biol Chem* 260: 1311-1325.

9. Furuta S, Miyazawa S, Osumi T, Hashimoto T & Ui N (1980) Properties of mitochondria and peroxisomal enoyl-CoA hydratases from rat liver. *Journal of Biochemistry* 88: 1059-1070.

10. Waterson RM & Hill RL (1972) Enoyl coenzyme A hydratase (crotonase). catalytic properties of crotonase and its possible regulatory role in fatty acid oxidation. *The Journal of Biological Chemistry* 247: 5258-5265.

11. Uchida Y, Izai K, Orii T & Hashimoto T (1992) Novel fatty acid beta-oxidation enzymes in rat liver mitochondria. II. purification and properties of enoyl-coenzyme A (CoA) hydratase/3-hydroxyacyl-CoA dehydrogenase/3-ketoacyl-CoA thiolase trifunctional protein. *The Journal of Biological Chemistry* 267: 1034-1041.

12. Osumi T & Hashimoto T (1980) Purification and properties of mitochondrial and peroxisomal 3-hydroxyacyl-CoA dehydrogenase from rat liver. *Archives of Biochemistry and Biophysics* 203: 372-383.

13. Kobayashi A, Jiang LL & Hashimoto T (1996) Two mitochondrial 3-hydroxyacyl-CoA dehydrogenases in bovine liver. *Journal of Biochemistry* 119: 775-782.

14. Middleton B (1973) The oxoacyl-coenzyme A thiolases of animal tissues. *Biochemical Journal* 132: 717.

15. Miyazawa S, Furuta S, Osumi T, Hashimoto T & Ui N (1981) Properties of peroxisomal 3-ketoacyl-coA thiolase from rat liver. *Journal of Biochemistry* 90: 511-519.

16. Horie S, Isobe M & Suga T (1986) Changes in CoA pools in hepatic peroxisomes of the rat under various conditions. *Journal of Biochemistry* 99: 1345-1352.

17. Stoll B, Gerok W, Lang F & Häussinger D (1992) Liver cell volume and protein synthesis. *The Biochemical Journal* 287 (Pt 1: 217-222.

18. Gear AR & Bednarek JM (1972) Direct counting and sizing of mitochondria in solution. *The Journal of Cell Biology* 54: 325-345.
